# Halogen-Dependent Diversity and Weak Interactions in the Heterometallic Ni/Cd Complex Solids: Structural and Theoretical Investigation

**DOI:** 10.3390/molecules28227652

**Published:** 2023-11-18

**Authors:** Oksana V. Nesterova, Svitlana R. Petrusenko, Brian W. Skelton, Dmytro S. Nesterov

**Affiliations:** 1 Centro de Química Estrutural, Institute of Molecular Sciences, Instituto Superior Técnico, Universidade de Lisboa, Av. Rovisco Pais, 1049-001 Lisbon, Portugal; dmytro.nesterov@tecnico.ulisboa.pt; 2 Department of Chemistry, Taras Shevchenko National University of Kyiv, 64/13 Volodymyrska Str., 01601 Kyiv, Ukraine; spetrusenko@yahoo.com; 3 Department of Inorganic Chemistry and Technology, Jožef Stefan Institute, Jamova 39, SI-1000 Ljubljana, Slovenia; 4 School of Molecular Sciences, M310, University of Western Australia, 35 Stirling Hwy, Perth, WA 6009, Australia; brian.skelton@uwa.edu.au

**Keywords:** nickel, cadmium, heterometallic complexes, ethylenediamine, DFT calculations, reaction mechanisms

## Abstract

Three novel heterometallic Ni/Cd coordination compounds [Ni(en)_3_][CdCl_4_]∙3dmso (**1**), [Ni(en)_2_(dmf)_2_][CdBr_4_] (**2**), and [Ni(en)_3_]_2_[CdI_4_](I)_2_ (**3**) have been synthesized through the self-assembly process in a one-pot reaction of cadmium oxide, nickel salt (or nickel powder), NH_4_X (X = Cl, Br, I), and ethylenediamine in non-aqueous solvents dmso (for **1**) or dmf (for **2** and **3**). Formation of the one- (**1**) or three-dimensional (**2** and **3**) hydrogen-bonded frameworks has been observed depending on the nature of the [CdX_4_]^2−^ counter-anion, as well as on the nature of the solvent. The electronic structures of [Ni(en)_3_]^2+^ and [Ni(en)_2_(dmf)_2_]^2+^ cations were studied at the DFT and CASSCF levels, including the ab initio ligand field theory (AILFT) calculations. The non-covalent intermolecular contacts between the cationic nickel and anionic cadmium blocks in the solid state were investigated by the QTAIM analysis. The mechanism of ligand substitution at the nickel center in [Ni(en)_2_(dmf)_2_]^2+^ was theoretically investigated at the ωB97X-D4/ma-def2-TZVP//DLPNO-CCSD(T)/ma-def2-TZVPP level. The results demonstrate that thermodynamic factors are structure-determining ones due to low energy barriers of the rotation of dmf ligands in [Ni(en)_2_(dmf)_2_]^2+^ (below 3 kcal mol^−1^) and the reversible transformation of [Ni(en)_2_(dmf)_2_]^2+^ into [Ni(en)_3_]^2+^ (below 20 kcal mol^−1^).

## 1. Introduction

Supramolecular bonding is a trending investigation topic in current chemical and biological research since it is involved in natural processes as well as in the design of novel materials [1,2]. Non-covalent interactions, such as hydrogen bonding and π···π stacking, are responsible for the construction of complex supramolecular multidimensional networks [1,3,4]. The design of solid architectures comprising the metal-based coordination blocks bridged by weak interactions is of special importance for the creation of next-generation materials for heterogeneous catalysis, gas storage, and other applications [5,6,7]. This approach allows fine-tuning of magnetic, spectroscopic, and catalytic properties of materials by altering the coordination environments of metal centers, while joining the complex molecules through the weak interactions may lead to the generation of topologies that are inaccessible for “classic” coordination-bonded metal–organic frameworks [1]. Heterometallic coordination compounds of transition metals attract special attention due to the fascinating properties (e.g., catalytic and magnetic ones) arising from the synergic interaction of several dissimilar metals located in close proximity [8,9,10].

Previously, we reported a series of homo- and heterometallic coordination compounds of various compositions and structures that have been synthesized through the synthetic approach called “direct synthesis of coordination compounds” [9,11]. Within the framework of this strategy, the coordination compounds are formed by the self-assembly reaction of building blocks, formed in situ, starting from elemental metals or their metal oxides and simple, conformationally flexible ligands. This strategy proceeds in a single-pot reaction, avoiding the multiple and individual steps of building block construction [11]. The direct synthesis approach has been widely applied to the synthesis of heterometallic 3d/3d solids, resulting in uncommon product compositions, network topologies, and properties [12,13,14], while the 3d/4d combinations are much less explored. The use of the 3d/4d metal pairs under the conditions of direct synthesis allows to obtain multidimensional materials where the 4d metal exhibits a strong influence on the magnetic [15,16,17] and catalytic performance [18]. Moreover, the direct synthesis approach is advantageous for the preparation of heterotrimetallic compounds [19,20]. 

High-spin d^8^ coordination compounds of nickel(II) with the *S* = 1 triplet spin state are objects of spectroscopic studies [21,22]. The Ni^2+^/Ni^3+^ redox potential is in a range suitable for the generation of high-valent metal–oxo species in oxidative catalysis [23,24], such as hydroxylation and epoxidation of C–H bonds with peroxides [25]. The triplet and singlet ground spin states of the high-spin Ni^2+^ and Ni^3+^ species, respectively, are easily distinguished using EPR spectroscopy, thus providing a convenient method for in situ study of catalytic intermediates [26,27]. The properties of the nickel cationic blocks in the solid state can be efficiently influenced by the catalytically or spectroscopically inert counterions. Despite the large number of the structures of Ni/Cd compounds reported in the Cambridge Structural Database (293 hits for version 5.43) [28], only 18 of them belong to the class of supramolecular frameworks where {Ni}^n+^ and {Cd}^n−^ coordination blocks are covalently separated. 

Herein, in continuation of our research line, we describe the synthesis, structural features, and characterization of three Ni/Cd halide organoamine solids based on ethylenediamine ligand and featuring supramolecular 1D and 3D architectures. The electronic structures, properties, and specific non-covalent interactions between the complex blocks were studied through the DFT and ab initio calculations.

## 2. Results

### 2.1. Synthesis and Spectroscopic Characterization

The complexes were synthesized through one-step self-assembly reactions from cadmium oxide, nickel salt (chloride for **1** or bromide for **2**) or nickel powder (for **3**), and NH_4_X (X = Cl (**1**), Br(**2**), I(**3**)) in dmso (**1**) or dmf (**2** and **3**) solution of ethylenediamine, using molar ratios CdO:NiX_2_:NH_4_X:en = 1:1:2:3 in case of **1** and **2** and CdO:Ni:NH_4_X:en = 1:2:6:6 in case of **3**. The light-violet solutions obtained at the completion of the reactions afforded the light-violet crystals of the heterometallic complexes upon the addition of Pr^i^OH (for **1**) or diethyl ether (for **3**) to the reaction mixtures, while the light-blue crystals of **2** were obtained after repeated addition of Pr^i^OH to the solution obtained after addition of Pr^i^OH to the filtrate. Based on our previous investigations, the interactions proceeding in the reaction systems can be described by the following schemes:

CdO + NiCl_2_ + 2NH_4_Cl + 3en + 3dmso → [Ni(en)_3_][CdCl_4_]·3dmso + 2NH_3_ + H_2_O (1)

2CdO + 2NiBr_2_ + 4NH_4_Br + 5en + 2dmf → [Ni(en)_3_][CdBr_4_] + [Ni(en)_2_(dmf)_2_][CdBr_4_] + 4NH_3_ + 2H_2_O (2)

2Ni^0^ + CdO + 6NH_4_I + 6en + O_2_ → [Ni(en)_3_]_2_[CdI_4_](I)_2_ + 6NH_3_ + 3H_2_O (3)

In the case of the system containing the bromide anion, the two heterometallic compounds were isolated: [Ni(en)_3_][CdBr_4_] from the mother liquor and [Ni(en)_2_(dmf)_2_][CdBr_4_] from the filtrate, after addition of Pr^i^OH to the respective solution. The complexes **1** and **2** could be obtained not only from a system with a CdO:NiX_2_:en = 1:1:2 molar ratio, but also with CdO:NiX_2_:en = 1:1:3. The investigation of the influence of different solvents on the final product composition showed the formation of the complex [Ni(en)_3_][CdCl_4_] in the case of using dmf, CH_3_OH, and CH_3_CN solvents, and the compound [Ni(en)_3_][CdBr_4_] in the case of CH_3_OH and CH_3_CN. The molar ratio of the initial reagents as well as the use of dmf solvent is crucial for the isolation of **3**.

The IR spectra of **1**, **2**, and **3** in the range 4000–400 cm^−1^ are similar and show all the characteristic ethylenediamine frequencies: ν(N–H), ν(C–H), δ(NH_2_), ν(C–N), and ν(C–C) in the ranges 3340–3250, 2990–2890, 1590–1580, 1030–1020, and 980–950 cm^−1^, respectively (Figures S1–S3). Analysis of the spectra of **1** does not indicate the presence of the solvate dmso molecule in the complex due to the overlap of the ν(SO) frequency of dmso with the ν(C–N) stretching vibration of the ethylenediamine at 1020 cm^−1^. The strong band at 1670 cm^−1^ in the IR spectrum of **2** was attributed to the ν(CO) vibration of dmf (Figure S2).

### 2.2. Crystal Structures

**Figure 1:**
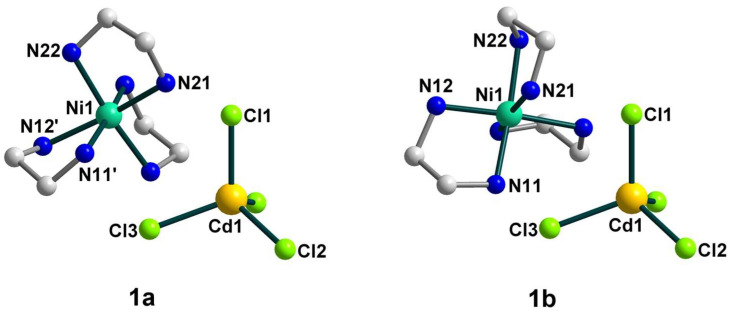
The structures of components **1a** (**left**) and **1b** (**right**) of **1** with the atom numbering schemes. H atoms and uncoordinated dmso molecules are omitted for clarity. Color scheme: Ni, blue-green; Cd, yellow; Cl, light green; N, blue; C, grey.

According to the X-ray diffraction analysis, the crystal structure of **1** is rather disordered and consists of two components (Figure 1) with a population of each of 0.5. In general, the structure represents an H-bonded supramolecular 1D chain constructed by two types of building blocks, discrete [Ni(en)_3_]^2+^ cations and [CdCl_4_]^2−^ anions as counterions, as well as three uncoordinated molecules of dimethylsulfoxide.

The nickel(II) atom is coordinated by six N atoms from three bidentate ethylenediamine ligands with Ni–N distances ranging from 2.084(9) to 2.183(9) Å (Table S1). The coordination sphere of [Ni(en)_3_]^2+^ is distorted, which can be seen from the N–Ni–N_trans_ angles varying from 168.9 to 174.43°. The bond distances and angles in the en ligands are in accordance with those found in similar nickel(II) complexes [29]. The Cd(II) coordination environment closely approximates tetrahedral symmetry, where the Cd–Cl bond lengths are in the range 2.426(2)–2.4430(18) Å and the Cl–Cd–Cl bond angles are 107.68(6)–113.83(8)°.

**Figure 2:**
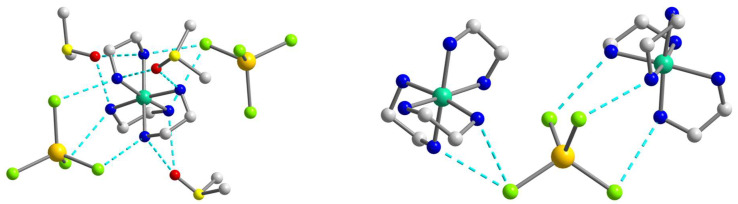
The supramolecular surroundings of the main building blocks (**left**—around [Ni(en)_3_]^2+^ and **right**—around [CdCl_4_]^2−^) in **1** (for the component **1a**). H atoms are omitted for clarity. Color scheme: Ni, blue-green; Cd, yellow; Cl, light green; N, blue; C, grey.

**Figure 3:**
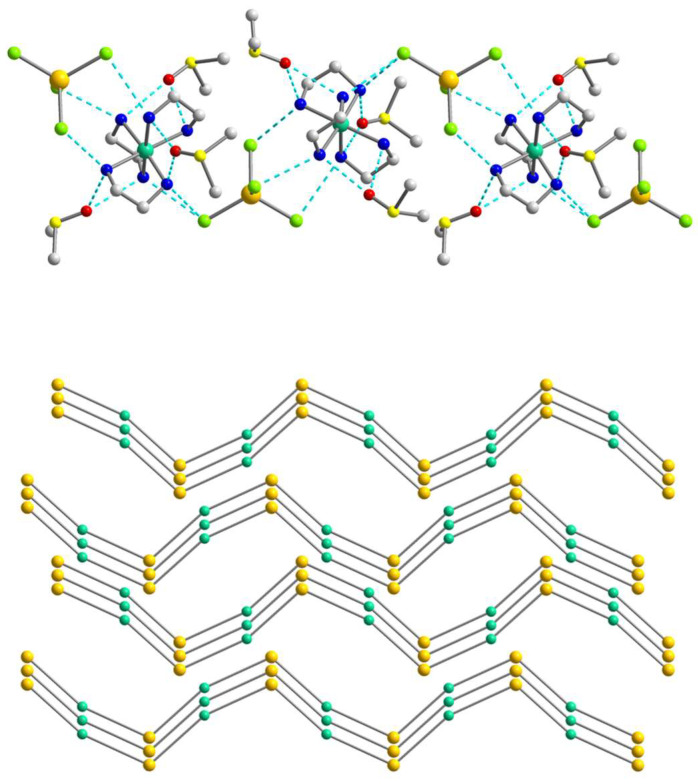
(**Top**): the representation of the supramolecular chain in **1a**. H atoms are omitted for clarity. Color scheme: Ni, blue-green; Cd, yellow; Cl, light green; N, blue; C, grey. (**Bottom**): packing of the supramolecular chains in **1a** that are drawn in the simplified topology. Color scheme: Ni, blue-green; Cd, yellow.

As the population of both components in the crystal structure of **1** is equal (0.5), the involvement in hydrogen bonding should be taken into consideration for each of them, but since they are quite similar, we decided to focus on the supramolecular structure of component **1a**. In assessing the H-bonding, the angles at the hydrogens were set to >140°. The supramolecular polymeric 1D structure of **1a** is fastened by numerous strong hydrogen bonds of two types, N–H···O and N–H···Cl, with the [Ni(en)_3_]^2+^ cation H-bonded to two different [CdCl_4_]^2−^ anions and three molecules of dmso, while the [CdCl_4_]^2−^ anion H-bonded to two neighboring [Ni(en)_3_]^2+^ cations (Figure 2 and Figure 3). All four Cl atoms in **1a** form hydrogen bonds to NH_2_ groups of [Ni(en)_3_]^2+^ cations, and one of them is 2-coordinated by H(N) atoms, and three others are unbranched. The N···Cl distances range from 3.258 to 3.659 Å and N–H···Cl angles are 144.81–166.07° (Table S4). Each O_dmso_ atom is involved in two H-bonds to H(N) atoms and the N···O distances of the en···solvate hydrogen bonds vary from 2.905(1) to 3.135(1) Å and N–H···O angles are in the range 153.95–164.62° (Table S4). The observed hydrogen bonds stabilize the overall supramolecular one-dimensional chains of **1a** (Figure 3) in an undulating shape. The further interchain connection did not occur due to the sterical hindrances caused by the presence of the dmso solvent molecules in the outlying positions of the chains. The nearest Ni∙∙∙Cd non-bonded separations within the supramolecular polymer are 5.229 and 5.259 Å, while the shortest Ni···Ni separation is 8.752 Å.

**Figure 4:**
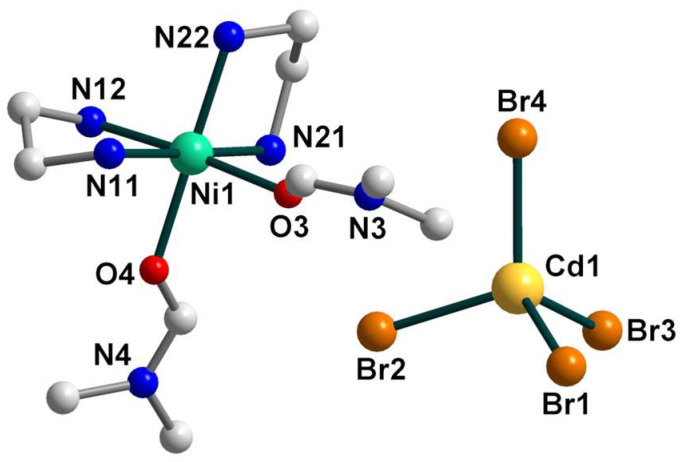
The structure of **2** with the atom numbering schemes. H atoms are omitted for clarity. Color scheme: Ni, blue-green; Cd, yellow; O, red; Br, dark orange; N, blue; C, grey.

The crystal structure of **2** consists of two types of blocks, [Ni(en)_2_(dmf)_2_]^2+^ and [CdBr_4_]^2−^, which form a three-dimensional network assisted by numerous hydrogen bonds. The Ni center is coordinated by four N atoms of two bidentate en ligands and two O atoms of coordinated dmf solvents (Figure 4). The coordination environment of the Ni(II) atom is best described as a slightly distorted octahedral with the N–Ni–N/O_trans_ angles varying from 175.59(14) to 179.48(14)° and Ni–N/O distances in the range 2.082(3)–2.109(3) Å (Table S2). The tetrahedral [CdBr_4_]^2−^ anion shows the Cd–Br bond distances in the range from 2.5595(6) to 2.6207(5) Å and the Br–Cd–Br bond angles are between 102.160(18) and 113.651(18)°. It should be noted, that **2** represents the first example of a complex containing the *cis*-isomer of the heteroleptic [M(en)_2_(dmf)_2_]^2+^ fragment which, according to the CSD, had not been previously structurally characterized for any transition metal.

**Figure 5:**
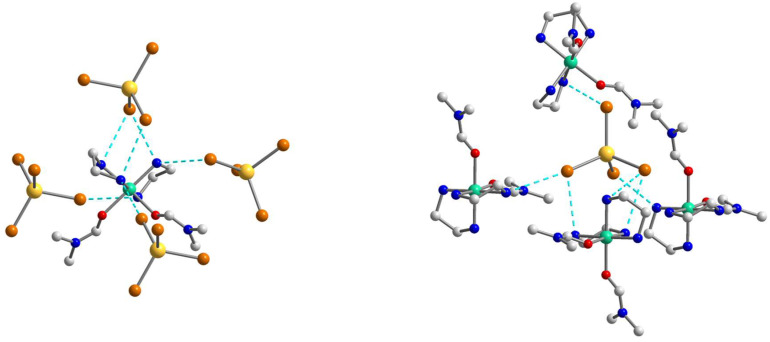
The supramolecular surroundings of the main building blocks (**left**—around [Ni(en)_2_(dmf)_2_]^2+^ and **right**—around [CdBr_4_]^2−^) in **2**. H atoms are omitted for clarity. Color scheme: Ni, blue-green; Cd, yellow; O, red; Br, dark orange; N, blue; C, grey.

**Figure 6:**
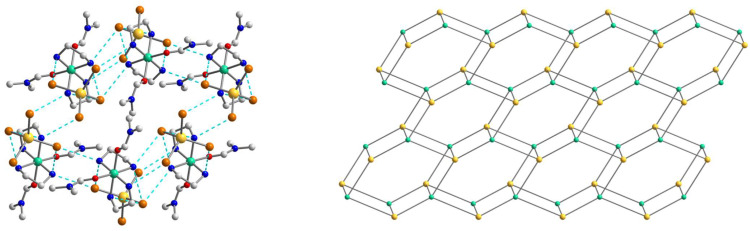
(**Left**): The representation of the supramolecular 3D network in **2**. H atoms are omitted for clarity. Color scheme: Ni, blue-green; Cd, yellow; O, red; Br, dark orange; N, blue; C, grey. (**Right**): The topology of the 3D network in **2**. Color scheme: Ni, blue-green; Cd, yellow.

The structure of **2** is fastened by numerous N–H···Br hydrogen bonds, with the [Ni(en)_2_(dmf)_2_]^2+^ cation H-bonded to four different [CdBr_4_]^2−^ anions, each of which, in turn, H-bonded to four neighboring cations (Figure 5). All Br atoms take part in hydrogen bonding with NH_2_ groups of en of the [Ni(en)_2_(dmf)_2_]^2+^, and two of them are 2-coordinated by H(N) atoms and the other two are unbranched. The N···Br lengths ranging from 3.529(4) to 3.742(4) Å and N–H···Br angles are within the range 149.1–171.8° (Table S4). The building blocks of **2** are packed into a 3D supramolecular network whose simplified topology is shown in Figure 6. The shortest Ni···Cd separation within the net is equal to 5.606 Å, while the nearest Ni···Ni distance is 7.903 Å.

**Figure 7:**
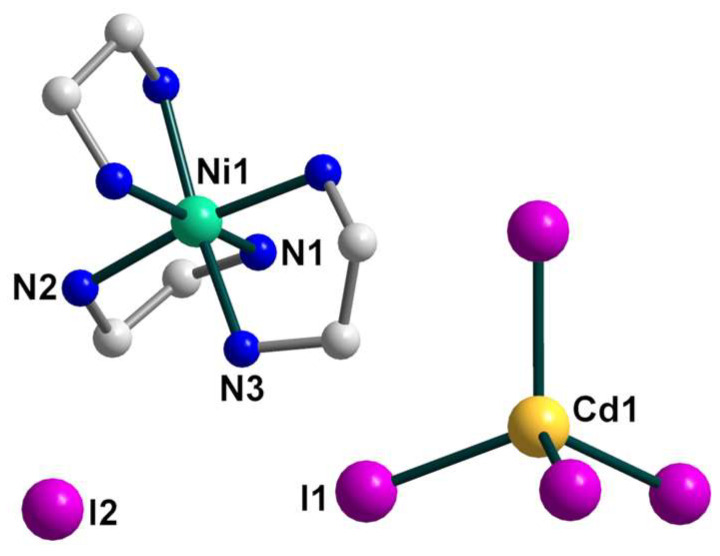
The structure of **3** with the atom numbering schemes. H atoms are omitted for clarity. Color scheme: Ni, blue-green; Cd, yellow; I, violet; N, blue; C, grey.

The crystal structure of **3** contains the same as in **1** [Ni(en)_3_]^2+^ cations, [CdI_4_]^2−^ anions, and two uncoordinated iodide anions (Figure 7), which linked into supramolecular three-dimensional network assisted by strong hydrogen bonds. The geometrical parameters of [Ni(en)_3_]^2+^ unit are like those found in **1**. The Cd coordination environment closely approximates tetrahedral symmetry, with the I–Cd–I bond angle being 109.142(5)–110.132(10)° (Table S3).

**Figure 8:**
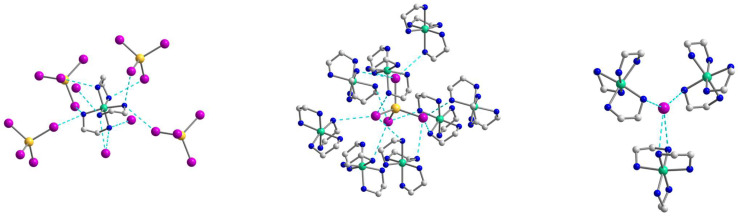
The supramolecular surroundings of the building blocks (**left**—around [Ni(en)_3_]^2+^, **middle**—around [CdI_4_]^2−^, and **right**—around I^2−^) in **3**. H atoms are omitted for clarity. Color scheme: Ni, blue-green; Cd, yellow; I, violet; N, blue; C, grey.

**Figure 9:**
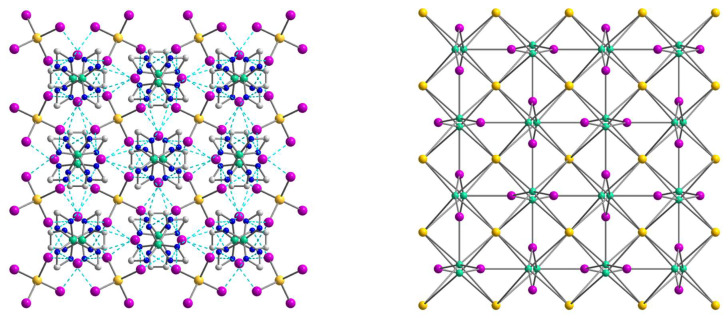
(**Left**): the representation of the supramolecular 3D network in **3**. H atoms are omitted for clarity. Color scheme: Ni, blue-green; Cd, yellow; I, violet; N, blue; C, grey. (**Right**): the topology of the 3D network in **3**. Color scheme: Ni, blue-green; Cd, yellow; I, violet.

A complex system of hydrogen bonds responsible for the formation of the supramolecular three-dimensional architecture is found in the crystal structure of **3** (Figure 8 and Figure 9). Each [Ni(en)_3_]^2+^ cation is surrounded by four [CdI_4_]^2−^ and three I^2−^ anions, while each [CdI_4_]^2−^ block is H-bonded to eight neighboring [Ni(en)_3_]^2+^ cations (Figure 8). All four I1 atoms of the [CdI_4_]^2−^ anion are 3-coordinated by H(N) atoms and form H bonds with NH_2_ groups of en ligands of cations showing the N···I1 distances in the range 3.774–3.972 Å and the N–H···I1 angles varying from 142.26 to 154.80° (Table S4). The non-coordinated I2 atom also takes part in H-bonding with amino groups of en of the cations, being 4-coordinated by H(N) atoms and surrounded by three [Ni(en)_3_]^2+^ units (Figure 8). The N···I2 distances are 3.708 and 3.728 Å, while N–H···I2 angles are 150.81 and 160.33° (Table S4). The shortest distance between iodine atoms I1···I2 is 4.590 Å, pointing to no specific interaction between them. Comparing the mean length values of hydrogen bonds formed by similar compounds [Cd(en)_3_]_2_[CdI_4_](I)_2_ (3.76 Å) [30], [Zn(en)_3_]_2_[CdI_4_](I)_2_ (3.81 Å) [31], and **3** (3.84 Å), one can conclude about stronger donor properties of [Cd(en)_3_]^2+^ cation in contrast to [Zn(en)_3_]^2+^ and [Ni(en)_3_]^2+^ ones. The nearest Ni∙∙∙Cd and Ni∙∙∙Ni separations within the supramolecular framework in **3** are 5.869 and 8.087 Å, respectively. 

### 2.3. Hirshfeld Surface Analysis

**Figure 10:**
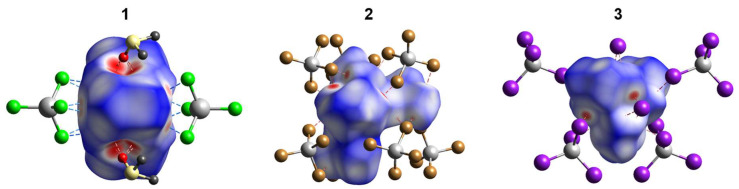
The Hirshfeld surfaces of [Ni(en)_3_]^2+^ and [Ni(en)_2_(dmf)_2_]^2+^ cations in **1**–**3** (both disordered components were included for **1**). The colored map corresponds to a normalized contact distance (*d*_norm_). Color scheme: Cd, grey; Cl, light green; Br, brown; I, violet; S, pale yellow; O, red; C, dark grey. Figures S4–S6 show the fingerprint plots (*d*_e_ vs. *d*_i_, Å) for cations of **1**–**3**.

**Figure 11:**
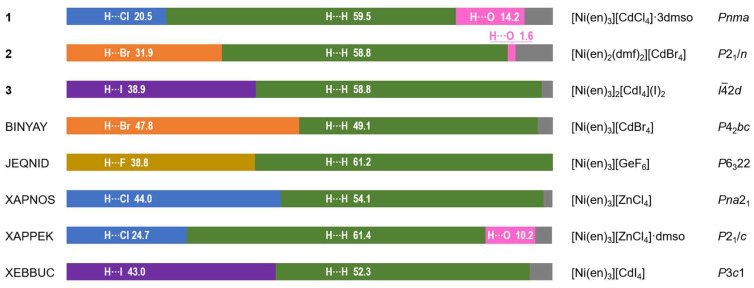
Contributions of H···Hal, H···H and H···O contacts to the Hirshfeld surface of [Ni(en)_3_]^2+^ and [Ni(en)_2_(dmf)_2_]^2+^ cations in **1**–**3** (both disordered components were included for **1**) and literature examples (CSD refcodes are indicated in the left, and formulae of compounds and space groups are indicated in the right). Contributions of other contacts are shown in grey color.

Analysis of the Hirshfeld surface (HS) [32] was performed to visualize the differences in environments around the nickel cations and their connectivity with [Cd(Hal)_4_]^2−^ anions in **1**–**3**. The normalized contact difference surfaces and selected fingerprint plots are shown in Figure 10 and Figure S4–S6, respectively. The shortest contacts corresponding to strong H···Hal and H···O hydrogen bonds are shown in red spots on the normalized surfaces (Figure 10). The contribution of the H···Hal contacts to the overall interactions increases from **1** to **3** (Figure 10 and Figure 11), reaching 38.9% for **3**. In all cases, a major part of the intermolecular interactions constitutes van der Waals H···H contacts with contribution higher than 50% (Figure 11). Analysis of the CSD data revealed such distribution of H···Hal and H···H contacts to be typical for the ionic structures based on [Ni(en)_3_]^2+^ cation (Figure 11). The highest percentage of non-directed H···H interactions (61.4%) is observed for [Ni(en)_3_][ZnCl_4_]·dmso complex [33], while the smallest one (49.1%) for [Ni(en)_3_][CdBr_4_] compound [34]. The lack of directionality of the H···H contacts results in a great topological diversity of [Ni(en)_3_]-based crystal structures, as can be illustrated by the variety of space groups of the examples discussed (Figure 11). 

### 2.4. Theoretical Studies

#### 2.4.1. Electronic Structures of [Ni(en)_3_]^2+^ and [Ni(en)_2_(dmf)_2_]^2+^ Cations

**Figure 12:**
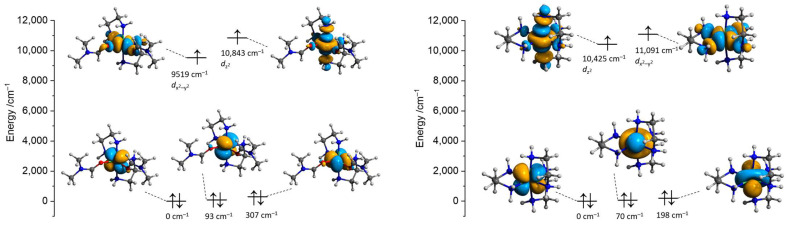
Isosurfaces and relative energies of the AILFT(SC-NEVPT2) nickel *d*-orbitals of the state-averaged CAS(8,5) active space of **2′** (**left**) and **3′** (**right**) calculated at the ZORA/ZORA-def2-TZVPP/CPCM level. Positions of H atoms were optimized at the ωB97X-D4/ma-def2-TZVP level, and the coordinates of all other atoms are from the crystallographic data.

The DFT and state-averaged CASSCF/SC-NEVPT2 calculations were performed to investigate the electronic structures of the nickel cations using the crystallographic atomic coordinates as the starting ones. Since the nickel cations in **1** and **3** are chemically equivalent and considering the positional disorder in the respective cation in **1**, the calculations of the [Ni(en)_3_]^2+^ fragment were performed only using the atomic coordinates of **3**. First, the positions of all hydrogen atoms were optimized at the ωB97X-D4/ma-def2-TZVP level, keeping the coordinates of all other atoms constrained. Optimization of H-atoms is an important step since the low scattering factor of a hydrogen atom leads to lower precision in the determination of respective atomic coordinates from the X-ray crystallographic data [35,36]. The CAS(8,5) active space was constructed at the ZORA/ZORA-def2-TZVPP level using the five *d*-orbitals of the nickel centers in terms of ab initio ligand field theory (AILFT). The unpaired electrons in both [Ni(en)_2_(dmf)_2_]^2+^ (**2′**) and [Ni(en)_3_]^2+^ (**3′**) cations are located at the *d*_z_^2^ and *d*_x_^2^_-y_^2^ orbitals (Figure 12), in agreement with the distorted *O*_h_ symmetry of the coordination environments around the nickel centers. The restricted open-shell DFT calculations (ROKS) at the PBE0/ZORA-def2-TZVPP level resulted in the SOMO orbitals of the same shape and order for **2′** and **3′** as for CASSCF ones (Figure S7). However, their mutual dispositions for **2′** and **3′** are different, and the *d*_z_^2^ orbital is of the highest energy for **2′**. The three lower-lying CAS AILFT orbitals are very close and become of mixed character after the SC-NEVPT2 correction. The Racah ligand field parameters *B* and *C* are shown in Listings S1 and S2. 

**Figure 13:**
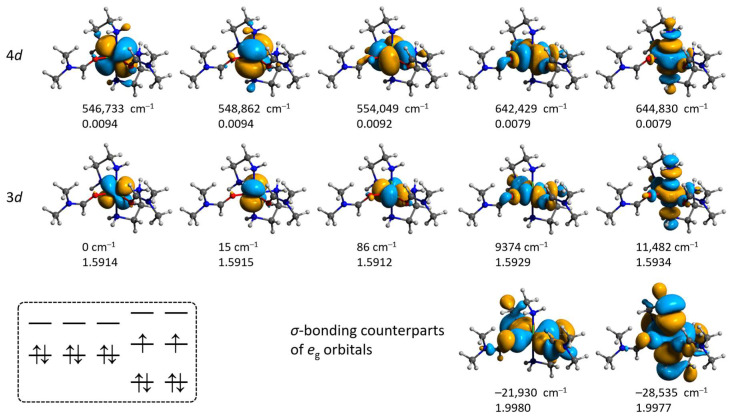
Isosurfaces and relative energies of the molecular orbitals for **2′** obtained from the CAS(12,12)/SC-NEVPT2 calculation at the ZORA/ZORA-def2-TZVPP/CPCM level. Positions of H atoms were optimized at the ωB97X-D4/ma-def2-TZVP level, and the coordinates of all other atoms are from the crystallographic data. The scheme in the left bottom illustrates the ground state configuration.

**Figure 14:**
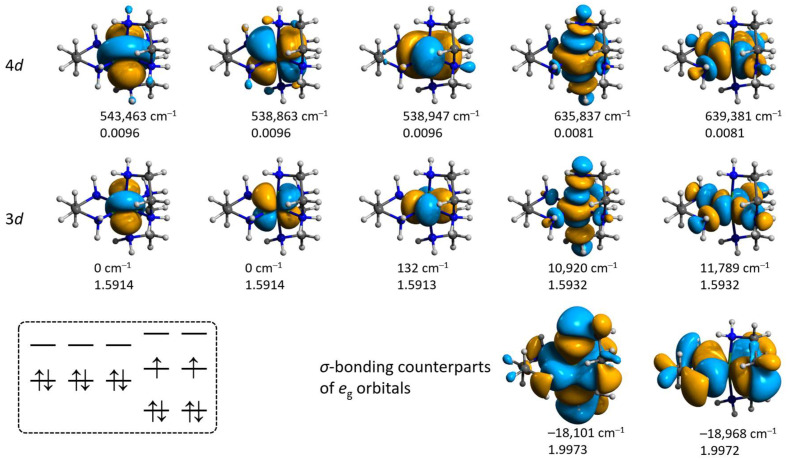
Isosurfaces and relative energies of the molecular orbitals for **3′** obtained from the CAS(12,12)/SC-NEVPT2 calculation at the ZORA/ZORA-def2-TZVPP/CPCM level. Positions of H atoms were optimized at the ωB97X-D4/ma-def2-TZVP level, coordinates of all other atoms are from the crystallographic data. The scheme in the left bottom illustrates the ground state configuration.

Expansion of the active space to the bonding counterparts of the singly occupied orbitals (SOMOs) as well as the 4d double shell does not alter the orbitals’ sequence, but brings the SOMO orbitals at slightly higher energy (Figure 13 and Figure 14). The CAS(8,5) → CAS(12,12) expansion had a notable influence on the calculated value of the zero-field splitting *D* of **3′**, which changes from +1.15 to −0.93 cm^−1^, respectively (Listings S1–S4). 

All the CASSCF calculations suggested the formal ground spin states of **2′** and **3′** to be a ^3^A_g_ triplet having a single configuration *t*^6^_2g_e^2^_g_ with a weight of 97%. The lowest singled state is located 14,620 and 14,592 cm^−1^ above the ground triplet state for **2′** and **3′**, respectively, as evidenced by the CAS(12,12)/SC-NEVPT2 results. These single states were interpreted as approximately equal mixtures of 20 and 02 configurations for both **2′** and **3′**, where the pairs of electrons are located either on *d*_z_^2^ or *d*_x_^2^_−y_^2^ orbitals (Listings S1–S4). The calculated absorption spectra are depicted in Figure S9. The energies and compositions of transitions predicted by different CASSCF and CASSCF/NEVPT2 calculations are similar. The lowest energy transitions for **2′** and **3′** correspond to the *d*_xy_ → *d*_x_^2^_−y_^2^ excitations, where the first excited triplet state is a single-reference one. The lowest-lying strong transitions ^3^A_g_ → ^3^B_1g_, ^3^A_g_ → ^3^B_2g,_ and ^3^A_g_ → ^3^B_3g_ are merged into a single peak in the 5000–15,000 cm^−1^ region (Figure S9). All other excited states are of strongly multireference character and involve various transitions between the double-occupied nearly degenerate *t*_2g_ orbitals and singly occupied orbitals that have a notable difference in energy (Listings S1–S4).

The experimental diffuse reflectance UV spectra for **1** and **2** containing [Ni(en)_3_]^2+^ and [Ni(en)_2_(dmf)_2_]^2+^ cations are shown in Figure S9. The spectra feature two broad absorption bands with the maxima at 18,320 and 28,692 cm^−1^ for **1**, and 18,360 and 27,863 cm^−1^ for **2**. These bands are as expected for spin-allowed ^3^T_1g_(^3^F) and ^3^T_1g_(^3^P) transitions for [Ni(en)_3_]^2+^ species [37] and other nearly-octahedral Ni(II) complexes [38]. The respective transitions predicted using the CASSCF calculations are composed of many multireference states (Listings S2 and S4) due to the symmetry of complexes lower than ideal *O*_h_. Among the CAS methods, the best agreement between the experimental and calculated data is obtained from the CAS(12,12) calculations with the subsequent NEVPT2 correction (Figure S9). The further improvement of the prediction accuracy for Ni(II) cations requires the use of computationally heavy DDCI3 and SORCI methods [39]. According to the TD-DFT calculations, the first *S*_1_ state is comprised of *d*_xy_ → *d*_x_^2^_−y_^2^ and mixed *d*_xz_/*d*_yz_ → *d*_z_^2^ excitations for **2′** and **3′**, respectively (Figure S8), similar to the CASSCF results (Listings S1–S4). However, the TD-DFT energies of the *S*_1_ states (14,037 and 15,435 cm^−1^ for **2′** and **3′**, respectively) are ca. 1.5 times overestimated as compared to the *S*_1_ triplet states from the CAS(12,12)/NEVPT12 calculation (9660 and 10,954 cm^−1^, respectively; Listings S3 and S4). Thus, the TD-DFT method fails to predict the transition energies showing a significant discrepancy (more than 5000 cm^−1^) between the experimental and predicted absorptions.

#### 2.4.2. Non-Covalent Interactions in the Lattices of 1–3

The crystal structures of all three complexes reveal numerous weak contacts between nickel cationic blocks, cadmium anionic blocks, solvent molecules (**1**), or iodide anions (**3**). The binding energies (BE) between the nickel and cadmium blocks calculated at the ωB97X-D4/ma-def2-QZVPP level appear to be of very high energies of ca. −200 kcal mol^−1^ magnitude per every pair of {Ni}^2+^···{Cd}^2−^ blocks (Table S5). These values are not surprising considering the strong electrostatic attraction of oppositely charged blocks, as well as the geometrical match between the halogen atoms of [CdHal_4_]^2−^ blocks and the corresponding NH_2_ groups of the cationic nickel blocks (Figure 2, Figure 5 and Figure 8).

The binding energy between the [Ni(en)_3_]^2+^ and [CdCl_4_]^2−^ blocks in **1** was calculated to be −257 kcal mol^−1^, while the respective interactions between the same nickel block and the nearest uncoordinated dmso molecules appear to be of considerably lower magnitude with BE between 29 and −35 kcal mol^−1^. The binding energies calculated for different components of the disordered [Ni(en)_3_]^2+^ in **1** appeared to be almost equal (Table S5). The [Ni(en)_2_(dmf)_2_]^2+^ cationic block in the crystal structure of **2** forms the non-covalent contacts with the seven nearest [CdBr_4_]^2−^ blocks (Figure 10), four of which form the N–H···Br bonds with the angles higher than 140° (Figure 5). The respective binding energies range from −156 to −240 kcal mol^−1^ (Table S5). The strongest interaction of −239 kcal mol^−1^ is observed with the [CdBr_4_]^2−^ block, for which the cadmium atoms are located at the *x*, *1 + y*, *z* position, with *d*(Ni···Cd) = 5.606 Å. The binding energies of the two intermolecular contacts between the [Ni(en)_3_]^2+^ and [CdI_4_]^2−^ blocks in **3** were estimated to be −185 and −242 kcal mol^−1^, *d*(Ni···Cd) = 7.978 and 5.869 Å, respectively (Table S5). The crystal structure of **3** features the non-coordinated iodide anion surrounded by three [Ni(en)_3_]^2+^ blocks (Figure 8). The respective binding energies were calculated to be −146 and −150 kcal mol^−1^ (Table S5). From the nearly linear dependence between the binding energy (BE) and Ni···Cd separation (Figure S11), one can assume that these energies have the largest contribution from the electrostatic interaction rather than the sum of contributions of individual hydrogen bonds between the blocks.

It is known that the energies of non-covalent interactions correlate with the electron densities in the respective (3, −1) bond critical points [40,41]. The [Ni(en)_3_]^2+^ block in the structure of **1** forms three strong N–H···Cl hydrogen bonds with the [CdCl_4_]^2−^ block (*x*, *y*, *z*) with the total *ρ*(**r**_BCP_) of 4.0 × 10^−2^ a.u., which can be interpreted as an attractive interaction (BE_BCP_) of −16.5 kcal mol^−1^, according to the linear regression developed by Emamiam and Lu [40]. The total electron densities of the N–H···O bond critical points for [Ni(en)_3_]^2+^···dmso interactions, *ρ*(**r**_BCP_) = 4.2 × 10^−2^ and 4.3 × 10^−2^ a.u. for contacts with O4 and O3 dmso oxygen atoms, respectively, appeared to be higher than that for the [Ni(en)_3_]^2+^···[CdCl_4_]^2−^ one, leading to the estimated BE_BCP_ = −17.2 and −17.6 kcal mol^−1^. For **2**, the strongest non-covalent interaction between nickel and cadmium blocks (BE = −239 kcal mol^−1^, Table S5) can be represented by seven bond critical points with the total *ρ*(**r**_BCP_) = 4.8 × 10^−2^ a.u. (BE_BCP_ = −23.3 kcal mol^−1^). Integration of the real space domain (Figure S12) designated by the reduced density gradient (RDG) function [42] afforded V = 1.42 Å^3^ and 0.048 a.u. of electrons. Optimization of the geometry of the respective [Ni(en)_2_(dmf)_2_][CdBr_4_] fragment at the ωB97X-D4/ma-def2-TZVP level afforded an even closer *d*(Ni···Cd) separation of 5.057 Å and a slight distortion of the tetrahedral geometry of the [CdBr_4_]^2−^ block. The interaction between the I^−^ anion (*x*, *y*, *z*) and the nickel block in **3** can be described by the two hydrogen bonds with *ρ*(**r**_BCP_) = 9.8 × 10^−3^ and 1.2 × 10^−2^ a.u., corresponding to the total BE_BCP_ of −9.3 kcal mol^−1^, while the binding energy calculated at the DFT level equals to −146 kcal mol^−1^ (Table S5). The four bond critical points can be found in the non-covalent area between the nickel cation and symmetrically transformed iodine atom I^a^ (^a^ = 1 + *y*, 1 − *x*, 1 − *z*), with total *ρ*(**r**_BCP_) = 3.6 × 10^−2^ a.u. and much higher total BE_BCP_ of −16.3 kcal mol^−1^. Analysis of the non-covalent interaction RDG isosurface revealed large domains that cannot be properly described by a few bond critical points (Figure S13). Integration of the respective domains resulted in V = 0.596 Å^3^ (0.026 a.u. of electrons) for the interaction with I and V = 0.889 Å^3^ (0.038 a.u. of electrons) for I^a^. 

As can be seen in all cases, the binding energies estimated from the electron densities at the critical points (BE_BCP_) are approximately one order less than the respective energies (BE) obtained from the DFT single-point calculations. This accounts for the limited applicability of the EB_BCP_ vs. *ρ*(**r**_BCP_) general dependencies for the cases where both interacting fragments are constructed from many atoms and possess a large charge.

#### 2.4.3. Rotation of the Coordinated dmf Ligand in **2′**

**Figure 15:**
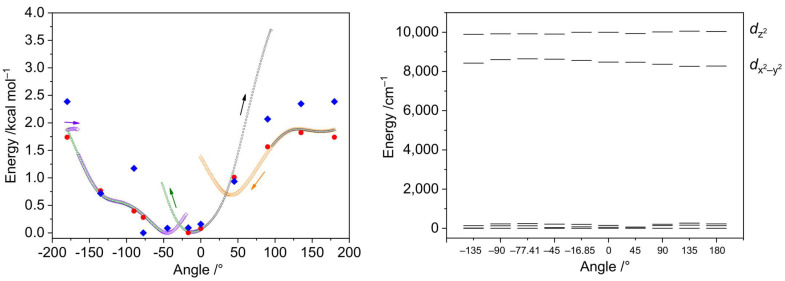
Left: relative energies of the [Ni(en)_2_(dmf)_2_]^2+^ cations depending on the torsion angle [O]_dmf1_–Ni–[O–C]_dmf2_. The cation geometry was relaxed, keeping the torsion angle constrained. Small black, green, violet, and yellow symbols represent the electronic energies from the relaxed scans using the r^2^SCAN-3c method, where the arrows indicate the directions of scans. Big red circles are the electronic energies obtained after geometry relaxation at the ωB97X-D4/ma-def2-TZVP level and selected angles, while big blue rhombs indicate the free Gibbs energies calculated at the same level. Right: relative energies of the AILFT(SC-NEVPT2) nickel *d*-orbitals of the CAS(8,5) active space calculated for ωB97X-D4/ma-def2-TZVP optimized structures with the [O]_dmf1_–Ni–[O–C]_dmf2_ torsion angle constrained at given values.

The DFT optimization of the geometry of the [Ni(en)_2_(dmf)_2_]^2+^ cation disclosed a notable alteration in the mutual disposition of the coordinated dmf molecules. One of the coordinated dmf ligands undergoes rotation to form the C–H···O hydrogen bond with the oxygen atom of the other coordinated dmf molecule, with *ρ*(**r**_BCP_) = 0.83 × 10^−2^ a.u. and BE_BCP_ = −1.1 kcal mol^−1^. The torsion angle [O]_dmf1_–Ni–[O–C]_dmf2_ changes from −77.41 to −16.85°. We were interested in investigating if this rotation can be caused by the high lattice energy of the crystal structure of **2**. The energy dependence on the dmf rotation angle is depicted in Figure 15, left. The relaxed scans of the respective torsion angle from 0° to 180° at two different directions were performed using the fast r^2^SCAN-3c composite method [43] that combines a meta-GGA r^2^SCAN functional with def2-mTZVPP basis set along with dispersion and counterpoise corrections. The r^2^SCAN-3c method was used due to its calculation speed and accuracy for weak interactions [44]. The scans disclosed that the lowest energy configuration can be found only after completing both direct and reverse scans (Figure 15, left). 

**Figure 16:**
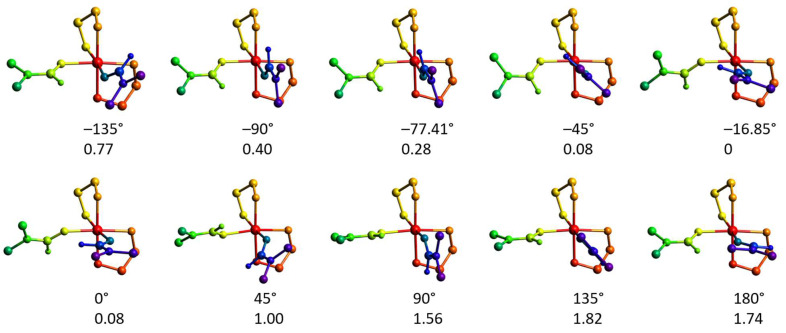
Molecular geometries of the [Ni(en)_2_(dmf)_2_]^2+^ cations optimized at the ωB97X-D4/ma-def2-TZVP level with the [O]_dmf1_–Ni–[O–C]_dmf2_ torsion angle constrained. The numbers indicate the angle (top, °) and electronic energy (bottom, kcal mol^−1^). The colors designate different parts of the molecule (relaxed and angle-constrained dmf ligands, green and violet, respectively; nickel atom, red; ethylenediamine ligands, yellow and orange).

**Figure 17:**
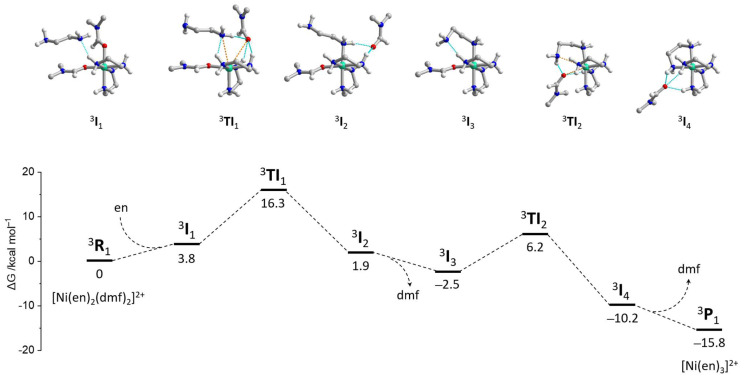
Free energy profile (**bottom**) of a reaction pathway for the ligand substitution in [Ni(en)_3_(dmf)_2_]^2+^ (^3^**R**_1_) with formation of [Ni(en)_3_]^2+^ (^3^**P**_1_), calculated at the ωB97X-D4/ma-def2-TZVP//DLPNO-CCSD(T)/ma-def2-TZVPP level, involving the C-PCM dmf solvation model. The structures of intermediates are shown in (**top**.)

The geometries and energies of the selected angle values were optimized using a range-separated functional supplied with the larger basis set (ωB97X-D4/ma-def2-TZVP), showing no significant differences from the r^2^SCAN-3c energies (Figure 15 and Figure 16). The AILFT calculations for these points revealed similar configurations for all geometries (Figure 15, right). The smallest *E*_dz2_ − *E*_dx2−y2_ gap of 1279 cm^−1^ was observed for the crystallographic angle of −77.41°, while the largest one (1796 cm^−1^) for 180° angle. The *t*_2g_ orbitals are nearly degenerate in all cases (Figure 17, right). The maximum single point energy change for complete dmf rotation does not exceed 2 kcal mol^−1^ and the maximum Gibbs energy change does not exceed 2.5 kcal mol^−1^, indicating that dmf ligands can adopt any configuration during the crystallization process.

#### 2.4.4. The Mechanism of Interconversion of [Ni(en)_2_(dmf)_2_]^2+^ and [Ni(en)_3_]^2+^

As the chelating en ligand is in excess relative to the metal in the synthetic protocols towards **1**–**3**, one may expect the formation of a stable [Ni(en)_3_]^2+^ cationic block as a product. However, the appearance of previously unknown cation [Ni(en)_2_(dmf)_2_]^2+^ in the structure of **2** suggests it can be a partially substituted intermediate during the formation of [Ni(en)_3_]^2+^. The complete pathway for the formation of [Ni(en)_3_]^2+^ should involve reversible stepwise substitution of all six dmf ligands in [Ni(dmf)_6_]^2+^, presumably formed at the first step. To determine if the isolation of the [Ni(en)_2_(dmf)_2_][CdBr_4_] compound **2** is governed by either kinetic or thermodynamic factors, we investigated the mechanism of [Ni(en)_2_(dmf)_2_]^2+^ + en → [Ni(en)_3_]^2+^ + 2dmf substitution reaction. 

The initial search for possible configurations was performed using the r^2^SCAN-3c method, while the final geometries and vibrational energies were obtained at the ωB97X-D4/ma-def2-TZVP level, where the electronic energy was refined at the DLPNO-CCSD(T)/ma-def2-TZVPP one (Table S6). Correction of the electronic energy using the DLPNO-CCSD(T) approximation [45,46] is among the best options for precise calculation of reaction barriers since DFT functionals may estimate this parameter with much less accuracy [44,47,48]. The analysis of the largest PNO amplitudes confirmed the absence of multireference character in all studied cases. From the experimental data, it is known that substitution at the Ni^2+^ ion proceeds through the dissociative mechanism [49]. However, attempts to model the dissociation of one dmf molecule did not afford stable intermediates, always leading to the coordination of a dmf molecule back to the nickel center. Considering that such dissociation should occur in the dmf medium, the respective equilibrium should be strongly shifted towards the starting [Ni(en)_2_(dmf)_2_]^2+^ compound. Therefore, we modeled the {[Ni(en)_2_(dmf)_2_]^2+^···(en)} supramolecular assembly ^3^**I**_1_ where ethylenediamine molecule is docked between two coordinated dmf ligands, strengthened by the N–H···N hydrogen bond with the coordinated en ligand (Figure 17). Although the binding energy between [Ni(en)_2_(dmf)_2_]^2+^ and en is negative, −8.4 kcal mol^−1^, the respective Gibbs energy change has a positive character (+3.8 kcal mol^−1^) associated with the entropy factor. 

Introduction of the ethylenediamine molecule into the coordination sphere of the nickel atom leads to the decoordination of one dmf molecule to produce the {[Ni(en)_2_(dmf)(en_monodentate_)]^2+^···(dmf)} intermediate ^3^**I**_2_ (Figure 17). The structure of ^3^**I**_1_ was found through the relaxed geometry scan of the *d*(Ni···N) distance, where the N atom belongs to the en molecule to be coordinated. The transition states initially found between the ^3^**I**_1_ and ^3^**I**_2_ intermediates revealed small imaginary frequencies of absolute magnitude lower than 100 cm^−1^. Although the largest imaginary vibration corresponds to the correct substitution pathway, attempts to refine the respective structures as true transition states were unsuccessful. The IRC scan disclosed small barriers of ca. 2 kcal mol^−1^ around the tentative transition states. Moreover, it was possible to refine their structures using the loose geometry optimization criteria to achieve a single structure ^3^**TI**_1_ with no imaginary frequencies. Thus, the potential energy surface (PES) around the presumable transition state is very flat, leading to many possible configurations. From this point of view, the configuration ^3^**TI**_1_ was considered a “transition intermediate”. The Gibbs energy gap between ^3^**I**_1_ and ^3^**TI**_1_ of 12.5 kcal mol^−1^ falls into the region of typical **I**-**TS** barrier height values. The coordination polyhedron of nickel in ^3^**TI**_1_ can be described as a tetragonal pyramid, as expected for the dissociative substitution mechanism. The Ni···N and Ni···O distances with interchanging en and dmf ligands constitute 3.322 and 3.411 Å, respectively. 

The further elimination of the decoordinated dmf molecule results in the rearrangement of the [Ni(en)_2_(dmf)(en_monodentate_)]^2+^ intermediate ^3^**I**_3_ where the uncoordinated amino group of the en ligand forms the N–H···N hydrogen bond with the other en ligand (Figure 17). The relaxed scan of the Ni···N distance (where N belongs to the uncoordinated NH_2_ group) allowed to obtain the supramolecular intermediate {[Ni(en)_3_]^2+^···(dmf)} (^3^**I**_4_), which eliminates the dmf molecule to produce the product [Ni(en)_3_]^2+^ (^3^**P**_1_). The latter process is favorable with the ΔG = −5.7 kcal mol^−1^. The search towards the transition state between ^3^**I**_3_ and ^3^**I**_4_ encountered the same issues as for the ^3^**TI**_1_ transition intermediate. Hence, the stable configuration ^3^**TI**_2_ was found with the Ni···N and Ni···O distances of 3.232 and 3.233 Å, respectively. 

The energies calculated at different levels of theory (r^2^SCAN-3c, B3LYP/ma-def2-SVP, ωB97X-D4/ma-def2-TZVP without CCSD correction) were to be very close to the ωB97X-D4/ma-def2-TZVP//DLPNO-CCSD(T)/ma-def2-TZVPP level (Figure S14, Table S6). The lowest barrier of 12.5 kcal mol^−1^ was obtained for the B3LYP/ma-def2-SVP scheme, while the r^2^SCAN-3c energies were found to be almost equal to the highest used calculation level. The lowest energy of the ^3^**P**_1_ (ΔG = −17.8 kcal mol^−1^) was found for the CCSD-uncorrected ωB97X-D4/ma-def2-TZVP level. 

Overall, the [Ni(en)_2_(dmf)_2_]^2+^ → [Ni(en)_3_]^2+^ transformation was found to be favorable with the total ΔG = −15.8 kcal mol^−1^. Although the exact transition states were not located due to the flat PES in the respective regions, one can estimate that the energies of the transition intermediates ^3^**TI**_1_ and ^3^**TI**_2_ are close to the transition state energies. Thus, the maximum barrier height of 16.3 kcal mol^−1^ was elucidated for this reaction. The Gibbs free energy of the reaction, as well as the relatively small barrier height, presume the reversible nature of this reaction, considering also that it proceeds in the dmf medium, which shifts the equilibrium towards the starting reagents [Ni(en)_2_(dmf)_2_]^2+^ + en.

Therefore, from the high binding energies between the nickel cationic {Ni}^2+^ and cadmium anionic {Cd}^2−^ blocks, which are superior the dmf rotation energies as well as the barriers between [Ni(en)_2_(dmf)_2_]^2+^ and [Ni(en)_3_]^2+^, one can conclude that the thermodynamic factor (the lattice energy) is decisive in the formation of **2′** or **3′** structural blocks.

## 3. Materials and Methods

All chemicals were of reagent grade and used as received. All experiments were carried out in air. Infrared spectra were recorded in KBr pellets on a UR-10 spectrophotometer in the 4000–400 cm^−1^ region using conventional techniques. 

### 3.1. Synthesis of [Ni(en)_3_][CdCl_4_]∙3dmso *(**1**)*

Cadmium oxide (0.32 g, 0.0025 mol), NiCl_2_·6H_2_O (0.59 g, 0.0025 mol), NH_4_Cl (0.27 g, 0.005 mol), dmso (20 cm^3^), and ethylenediamine (0.5 cm^3^, 0.0075 mol) were heated to 50–60 °C and stirred magnetically for 60 min. The light-violet crystals suitable for X-ray crystallography were obtained after the addition of 15 cm^3^ of Pr^i^OH to the reaction mixture. Yield: 0.58 g, 32%. Anal. calc. for C_12_H_42_CdCl_4_N_6_NiO_3_S_3_: Ni, 8.07; Cd, 15.45; Cl, 19.49; C, 19.81; H, 5.82; N, 11.55. Found: Ni, 7.8; Cd, 15.1; Cl, 19.6; C, 20.0; H, 5.9; N, 11.8%. IR (KBr, cm^−1^): 3320 s, 3280 sh, 3150 m, 2950 m, 2890 m, 1590 s, 1470 w, 1400 m, 1330 w, 1280 w, 1150 w, 1100 w, 1020 vs, 950 w, 720 w, 650 m, 530 m, 480 w.

### 3.2. Synthesis of [Ni(en)_2_(dmf)_2_][CdBr_4_] *(**2**)*

Cadmium oxide (0.32 g, 0.0025 mol), NiBr_2_·5H_2_O (0.77 g, 0.0025 mol), NH_4_Br (0.49 g, 0.005 mol), dmf (20 cm^3^), and ethylenediamine (0.5 cm^3^, 0.0075 mol) were heated to 50–60 °C and stirred magnetically for 20 min. The light-violet microcrystalline powder, later it was found to be a complex [Ni(en)_3_][CdBr_4_], was obtained after the addition of 10 cm^3^ of Pr^i^OH to the reaction mixture. Yield: 0.47 g, 28%. The light-blue crystals of [Ni(en)_2_(dmf)_2_][CdBr_4_] suitable for X-ray crystallography were obtained from the filtrate after addition of 25 cm^3^ of Pr^i^OH. Yield: 0.65 g, 35%. Anal. calc. for C_10_H_30_CdBr_4_N_6_NiO_2_: Ni, 7.75; Cd, 14.85; Br, 42.22; C, 15.86; H, 3.99; N, 11.10. Found: Ni, 7.6; Cd, 14.7; Br, 42.4; C, 15.9; H, 4.1; N, 11.3%. IR (KBr, cm^−1^): 3340 m, 3310 m, 3280 m, 2990 sh, 2960 m, 2890 w, 1670 vs, 1580 m, 1500 w, 1450 m, 1420 m, 1390 s, 1320 w, 1290 w, 1270 w, 1210 sh, 1150 sh, 1110 m, 1030 m, 1020 vs, 980 w, 780 w, 690 m, 650 m, 630 m, 590 w, 500 m, 420 w.

### 3.3. Synthesis of [Ni(en)_3_]_2_[CdI_4_](I)_2_ *(**3**)*

Cadmium oxide (0.32 g, 0.0025 mol), Ni powder (0.29 g, 0.005 mol), NH_4_I (2.17 g, 0.015 mol), dmf (20 cm^3^), and ethylenediamine (1 cm^3^, 0.015 mol) were heated to 50–60 °C and stirred magnetically for 12 h. The light-violet crystals suitable for X-ray analysis were obtained after the addition of 15 cm^3^ of diethyl ether to the reaction mixture. Yield: 0.36 g, 11%. Anal. calc. for C_12_H_48_CdI_6_N_12_Ni_2_: Ni, 8.68; Cd, 8.32; I, 56.33; C, 10.66; H, 3.58; N, 12.43. Found: Ni, 8.6; Cd, 8.5; I, 56.2; C, 10.3; H, 3.9; N, 12.2%. IR (KBr, cm^−1^): 3500 br, 3310 vs, 3250 vs, 3210 m, 3120 w, 2950 w, 2920 m, 2890 m, 1590 m, 1560 vs, 1450 m, 1390 w, 1320 m, 1270 m, 1140 w, 1090 m, 1080 w, 1040 w, 1020 vs, 980 m, 860 w, 640 s, 610 m, 510 m, 490 m, 470 m.

### 3.4. Crystallography

The crystal data for **1**–**3** are summarized in the Table 1. Crystallographic data for these three structures were collected at 150(2) K on a Bruker Smart diffractometer using Mo Kα radiation. Following multi-scan corrections and solution by direct methods, the structure was refined against *F*^2^ with full-matrix least-squares using the program SHELXL-2017 [50]. In **1**, the cation, anion, and one solvent dmso lie on a crystallographic mirror plane. As a result, the nitrogen atoms of the cation are disordered over two sites, each with an occupancy of 0.5. The sulfur atom of the solvent dmso on the mirror plane is also disordered over two sites with refined occupancies of 0.827(9), and 1–0.827(9). All hydrogen atoms in **1**–**3** were added at calculated positions and refined through the use of a riding model with isotropic displacement parameters based on those of the parent atom. Anisotropic displacement parameters were employed for the non-hydrogen atoms.

Crystallographic data for the structures reported can be obtained free of charge from the Cambridge Crystallographic Data Centre via www.ccdc.cam.ac.uk/data_request/cif, quoting the deposition numbers CCDC 2111366 (**1**), 1958573 (**2**), and 1955081 (**3**).

### 3.5. Theoretical Calculations

The ORCA 5.0.4 (Max-Planck-Institut für Kohlenforschung, Mülheim a. d. Ruhr, Germany) software package was used for all calculations [51,52,53]. Unless stated otherwise, geometry optimizations were performed using the range-separated ωB97X-D4 functional [54,55] with the minimally augmented ma-def2-TZVP basis sets [56]. Binding energies were calculated using the same functional but larger basis set ma-def2-QZVPP [56]. To obtain the binding energy (BE) of the A···B assembly, individual electronic energies E_AB_, E_A_, and E_B_ were calculated using unrelaxed atomic coordinates. The final BE was calculated according to the equation BE = E_AB_ − E_A_ − E_B_. The QTAIM [57] studies were performed using the same ωB97X-D4 functional. Both QTAIM and CASSCF calculations employed the ZORA relativistic approximation, SARC-def2-TZVPP basis set [58] for cadmium and iodine atoms and ma-ZORA-def2-TZVPP (for QTAIM) or ZORA-def2-TZVPP (for CASSCF and AILFT [59]) for all other atoms [56,60]. In certain cases, the r^2^SCAN-3c composite meta-GGA DFT method [43] and B3LYP hybrid meta-GGA functional [61,62] were employed, the latter accompanied by ma-def2-TZVP or ma-def2-SVP basis sets [56]. The SCF optimization convergence criteria were settled with *VeryTightSCF* keywords, and integration grids of high density (*Defgrid3* keyword) were employed. Dispersion correction was introduced through the *D4* keyword (Grimme’s atom-pairwise approach) [63]. The *AutoAux* keyword [64] was used to generate auxiliary basis sets in all cases. The SARC/J auxiliary basis set [58,65] was applied when using ZORA approximation. The CASSCF/AILFT calculation was invoked by the *actorbs dorbs* keyword, while the 4d orbitals for CAS(12,12) calculation were generated through the *extorbs doubleshell* keywords. The σ-bonding counterpart orbitals were found from the visual inspection of orbitals isosurfaces, as well as from the respective atomic contributions. All convergence thresholds for CASSCF calculations were as default (gradient threshold ‖g‖ < 1 × 10^−3^), except of the D4Tpre parameter for NEVPT2 procedure, which was set to 1 × 10^−14^. The selected CASSCF outputs are shown in Listings S1–S4.

The time-dependent DFT calculations (TD-DFT) were performed at the PBE0 functional [66,67] and ZORA-def2-TZVPP basis set using the Tamm–Dancoff approximation (TDA) [68]. The crystal field for CASSCF and TD-DFT calculations was accounted for by means of the C-PCM model [69] with *ε* = infinity. The strongly contracted *n*-electron valence state perturbation theory (SC-NEVPT2) [70,71] was applied with CASSCF/AILFT calculations. The coupled cluster calculations were performed through the DLPNO-CCSD(T) scheme [45,46] using the ma-def2-TZVPP basis set [56] and *TightPNO* keyword. The RIJK [72] and RIJCOSX [73] approximations were used for CASSCF/SC-NEVPT2 and all other calculations, respectively. The solvent (dmf) effects were accounted for by means of the C-PCM model [69]. The correction Δ*G* term of 1.89 kcal mol^−1^ was added to the final Gibbs energies of single molecules to convert 1 atm to 1 M standard states [74]. The searches for transition states were performed using either nudged elastic band (NEB) [75] or relaxed scan methods. The visualization of molecular orbitals was made using the Avogadro 1.2 (University of Pittsburgh, Pittsburgh, PA, USA) program [76]. Analysis of bond critical points and non-covalent interactions indexes [42] was performed using the Multiwfn 3.8 (University of Science and Technology, Beijing, People’s Republic of China) programme [77]. The visualization of the non-covalent reduced density gradient (RDG) [42] isosurfaces was made using Visual Molecular Dynamics 1.9.3. (University of Illinois at Urbana–Champaign, Urbana, IL, USA) programme [78]. Hirshfeld analysis [32] and surface visualization were made using the CrystalExplorer 21.5 (University of Western Australia, Perth, Australia) program [79]. 

## 4. Conclusions

The heterometallic Ni/Cd compounds were synthesized via self-assembly reactions of cadmium oxide, nickel salt (or nickel powder), and ammonium salt (NH_4_Cl, NH_4_Br, and NH_4_I) with non-aqueous solutions (dmso or dmf) of ethylenediamine, and characterized by X-ray diffraction analysis. Despite similar compositions, the crystal structures of **1**–**3** feature three different supramolecular organizations of nickel and cadmium building blocks where the formation of the 1D chain (in **1**) and 3D framework architectures (in **2** and **3**) were observed. The *cis*-isomer of a heteroleptic complex cation [M(en)_2_(dmf)_2_]^2+^ was, for the first time, characterized by single-crystal X-ray diffraction. The electronic structures of [Ni(en)_2_(dmf)_2_]^2+^ and [Ni(en)_3_]^2+^ cations have been investigated via the DFT and CASSCF methods, including the ab initio ligand field theory (AILFT). The non-covalent interactions between the complex cationic and anionic blocks were studied from the point of view of a quantum theory of atoms in ligands (QTAIM), showing that the binding energies predicted from the electron densities at the (3, −1) bond critical points are considerably underestimated when compared to the energies calculated from the electronic energies. The rotation of the dmf molecule in the complex cation [Ni(en)_2_(dmf)_2_]^2+^ was investigated and it was found that the rotation energy does not exceed 2 kcal mol^−1^. The mechanism of dmf substitution for an ethylenediamine ligand in the coordination sphere of nickel was investigated at the ωB97X-D4/ma-def2-TZVP//DLPNO-CCSD(T)/ma-def2-TZVPP level of theory. The reaction barrier for the [Ni(en)_2_(dmf)_2_]^2+^ → [Ni(en)_3_]^2+^ transformation in dmf medium was found to be 16.3 kcal mol^−1^. It was concluded that the nature of the reaction product in the formation of **1**–**3** is likely determined by the thermodynamic factors rather than kinetic ones.

## Figures and Tables

**Table 1:** Crystal data and structure refinement for **1**–**3**.

	1	2	3
Empirical formula	C_12_H_42_CdCl_4_N_6_NiO_3_S_3_	C_10_H_30_Br_4_CdN_6_NiO_2_	C_12_H_48_CdI_6_N_12_Ni_2_
Formula weight	727.60	757.15	1351.84
Temperature, K	150(2)	150(2)	150(2)
Wavelength, Å	0.71073	0.71073	0.71073
Crystal system	Orthorhombic	Monoclinic	Tetragonal
Space group	*Pnma*	*P*2_1_/*n*	*I*$\bar{4}$2*d*
*a*, Å	17.298(4)	12.3920(12)	14.6310(10)
*b*, Å	13.884(3)	11.3388(11)	14.6310(10)
*c*, Å	12.558(3)	17.672(2)	16.824(2)
*α*, °	90	90	90
*β*, °	90	108.043(2)	90
*γ*, °	90	90	90
Volume, Å^3^	3016.0(12)	2361.0(4)	3601.4(7)
*Z*	4	4	4
Density (calculated), Mg/m^3^	1.602	2.130	2.493
μ, mm^−1^	1.915	8.482	6.787
F(000)	1488	1456	2504
Crystal size, mm^3^	0.450 × 0.180 × 0.060	0.550 × 0.240 × 0.220	0.160 × 0.160 × 0.130
θ range for data collection, °	2.004 to 25.174	1.777 to 27.00	1.845 to 37.552
Index ranges	−20 ≤ h ≤ 20, −16 ≤ k ≤ 16, −14 ≤ l ≤ 14	−16 ≤ h ≤ 16, −15 ≤ k ≤ 15, −24 ≤ l ≤ 24	−25 ≤ h ≤ 25, −25 ≤ k ≤ 25, −28 ≤ l ≤ 28
Reflections collected	22,087	22,374	35,196
Independent reflections	2818 [*R*(int) = 0.069]	5146 [*R*(int) = 0.052]	4747 [*R*(int) = 0.033]
Completeness to θ = 25.174° (for **1**), 25.242° (for **2**) and 25.242° (for **3**), %	99.5	99.9	100.0
Refinement method	Full-matrix least-squares on *F*^2^	Full-matrix least-squares on *F*^2^	Full-matrix least-squares on *F*^2^
Data/restraints/parameters	2818/0/191	5146/0/221	4747/0/76
Goodness-of-fit on *F*^2^	1.091	0.989	1.113
Final *R* indices [*I* > 2σ(*I*)]	*R*1 = 0.0434, *wR*2 = 0.1003	*R*1 = 0.0336, *wR*2 = 0.0818	*R*1 = 0.0198, *wR*2 = 0.0391
*R* indices (all data)	*R*1 = 0.0785, *wR*2 = 0.1241	*R*1 = 0.0480, *wR*2 = 0.0873	*R*1 = 0.0221, *wR*2 = 0.0397
Largest diff. peak and hole, e Å^−3^	1.121 and −0.564	1.421 and −1.094	1.125 and −0.412
Absolute structure parameter	–	–	0.002(11)

## Data Availability

Crystallographic data for the structures can be obtained free of charge from the Cambridge Crystallographic Data Centre via www.ccdc.cam.ac.uk/data_request/cif, quoting the deposition numbers CCDC 2111366 (**1**), 1958573 (**2**) and 1955081 (**3**).

## Supplementary Materials

The following supporting information can be downloaded at: https://www.mdpi.com/article/10.3390/molecules28227652/s1, Figure S1: IR spectrum of [Ni(en)_3_][CdCl_4_]·3dmso (**1**). Figure S2: IR spectrum of [Ni(en)_2_(dmf)_2_][CdBr_4_] (**2**). Figure S3: IR spectrum of [Ni(en)_3_]_2_[CdI_4_](I)_2_ (**3**). Figures S4–S6: The selected fingerprint plots for **1**–**3**. Figure S7: Isosurfaces of the singly occupied molecular orbitals for **2′** and **3′** showing the energy gaps, calculated at the open-shell spin restricted PBE0/ZORA-def2-TZVPP level. Figure S8: Isosurfaces of the natural transition orbitals for **2′** and **3′** involved in the first excited state transitions calculated at the TD-DFT PBE0/ZORA-def2-TZVPP level. Figure S9: Fragments of the absorption spectra for **2′** and **3′** calculated at different levels of theory. Figure S10: Experimental diffuse reflectance spectra of **1** and **2** along with the theoretical ones for **2′** and **3′** calculated at the CAS(12,12)/NEVPT2 level. Figure S11: Dependence of the binding energy on the distance between the nickel and cadmium centers in the {Ni}^2+^{Cd}^2−^ supramolecular assembly, where {Cd}^2−^ stands for [CdCl_4_]^2−^, [CdI_4_]^2−^ or [CdBr_4_]^2−^. Figure S12. Non-covalent interactions domain calculated using the reduced density gradient in the {[Ni(en)_2_(dmf)_2_]^2+^···[CdBr_4_]^2−^} assembly in **2**, where the symmetry operation for the cadmium center is *x*, 1 + *y*, *z*. Figure S13: Isosurface of the RDG illustrating non-covalent interactions in the {I··· [Ni(en)_3_]···I^a^} fragment. Figure S14: Free energy profiles of a reaction pathway for the ligand substitution in [Ni(en)_3_(dmf)_2_]^2+^ (^3^R_1_) with formation of [Ni(en)_3_]^2+^ (^3^P_1_), calculated at the indicated levels of theory and involving the C-PCM dmf solvation model. Table S1: Selected geometrical parameters (distances/Å and angles/°) for 1. Table S2: Selected geometrical parameters (distances/Å and angles/°) for **2**. Table S3: Selected geometrical parameters (distances/Å and angles/°) for 3. Table S4: Hydrogen bonding distances (Å) and angles (°) for complexes 1a–3. Table S5: Binding energies of the selected blocks in **1**–**3** calculated at the ωB97X-D4/ma-def2-QZVPP level. Table S6: Gibbs free energies of the molecular fragments involved in the ^3^R_1_ → ^3^P_1_ reaction. Table S7: Maxima of the Gaussian lineshapes of the transitions calculated at the stated levels. Listings S1: Selected output of the state-averaged CAS(8,5)/AILFT(SC-NEVPT2) calculation for **2′**. Listings S2: Selected output of the state-averaged CAS(8,5)/AILFT(SC-NEVPT2) calculation for **3′**. Listings S3: Selected output of the state-averaged CAS(12,12)/SC-NEVPT2 calculation for **2′**. Listings S4: Selected output of the state-averaged CAS(12,12)/SC-NEVPT2 calculation for **3′**. Listings S5: Cartesian coordinates of [Ni(en)_2_(dmf)_2_]^2+^ and [Ni(en)_3_]^2+^ with the positions of H atoms optimized at the ωB97X-D4/ma-def2-TZVP level. Listings S6: Cartesian coordinates of [Ni(en)_2_(dmf)_2_]^2+^ and [Ni(en)_3_]^2+^ with the positions of all atoms optimized at the ωB97X-D4/ma-def2-TZVP level. Listings S7: Cartesian coordinates of [Ni(en)_2_(dmf)_2_]^2+^ with the positions of all atoms optimized at the ωB97X-D4/ma-def2-TZVP level, keeping the [O]_dmf1_–Ni–[O–C]_dmf2_ torsion angle constrained to the indicated value. Listings S8: Cartesian coordinates of the starting reagents, product, and intermediate for the ^3^R_1_ → ^3^P_1_ reaction.
