# Supplementary material for: Halogen-Dependent Diversity and Weak Interactions in the Heterometallic Ni/Cd Complex Solids: Structural and Theoretical Investigation
Source: Molecules. 2023 Nov 18;28(22):7652. doi: 10.3390/molecules28227652 (PMC10674445; doi:10.3390/molecules28227652)
Supplement: Supplementary file 1 [file molecules-28-07652-s001.zip › molecules-2721724-supplementary.pdf]

Supplementary Information

Halogen-Dependent Diversity and Weak Interactions in the Heterometallic Ni/Cd Complex Solids: Structural and Theoretical Investigation

Oksana V. Nesterova [1], Svitlana R. Petrusenko [2,3], Brian W. Skelton [4], and Dmytro S. Nesterov [1]*

[1] *Centro de Química Estrutural, Institute of Molecular Sciences, Instituto Superior Técnico, Universidade de Lisboa, Av. Rovisco Pais, 1049-001, Lisbon, Portugal*

[2] *Department of Chemistry, Taras Shevchenko National University of Kyiv, 64/13 Volodymyrska str., Kyiv 01601, Ukraine*

[3] *Department of Inorganic Chemistry and Technology, Jožef Stefan Institute, Jamova 39, SI-1000 Ljubljana, Slo-venia*

[4] *School of Molecular Sciences, M310, University of Western Australia, 35 Stirling Hwy, Perth, WA 6009, Australia*

** Correspondence: oksana.nesterova@tecnico.ulisboa.pt*

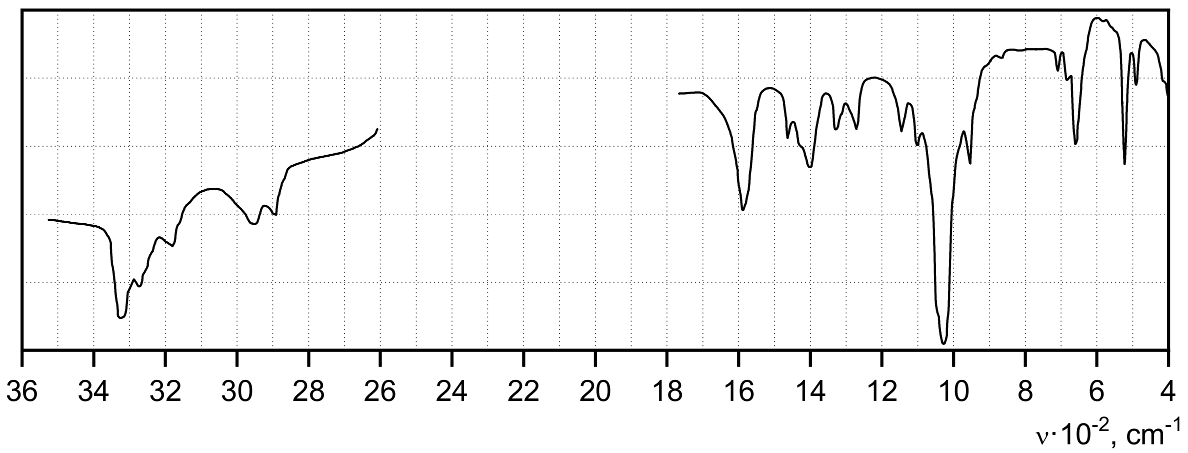

Figure S1. IR spectrum of $[Ni(en)_3][CdCl_4]\cdot 3dmso$ (**1**).

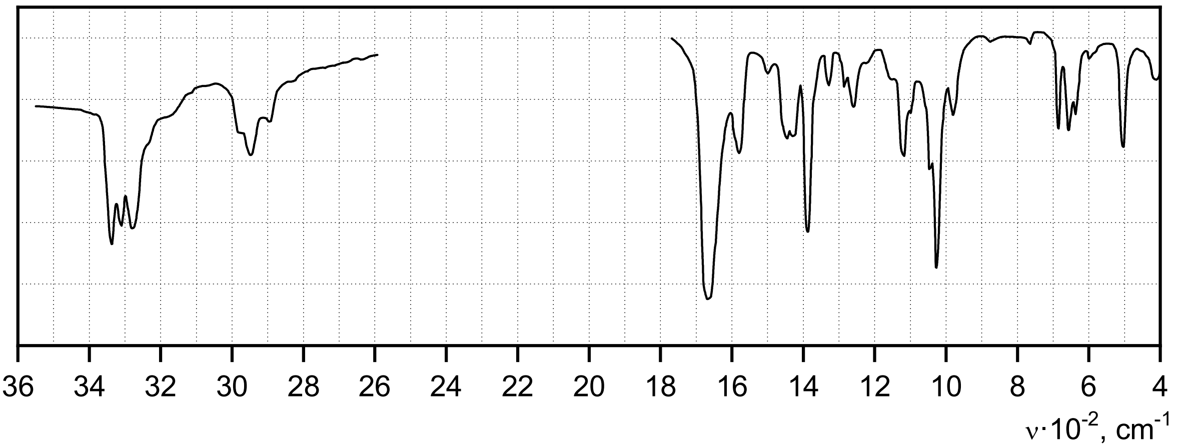

Figure S2. IR spectrum of $[Ni(en)_2(dmf)_2][CdBr_4]$ (**2**).

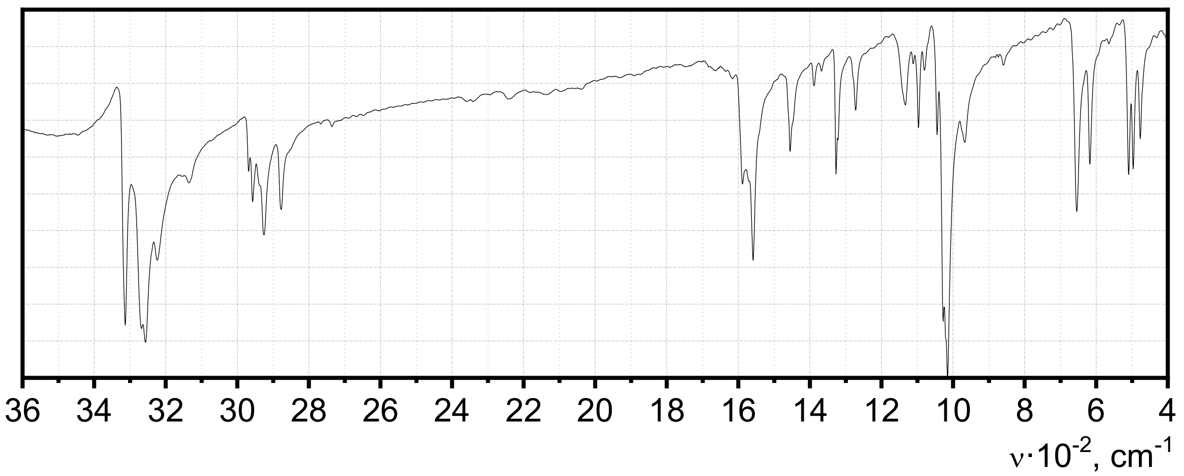

Figure S3. IR spectrum of $[Ni(en)_3]_2[CdI_4](I)_2$ (**3**).

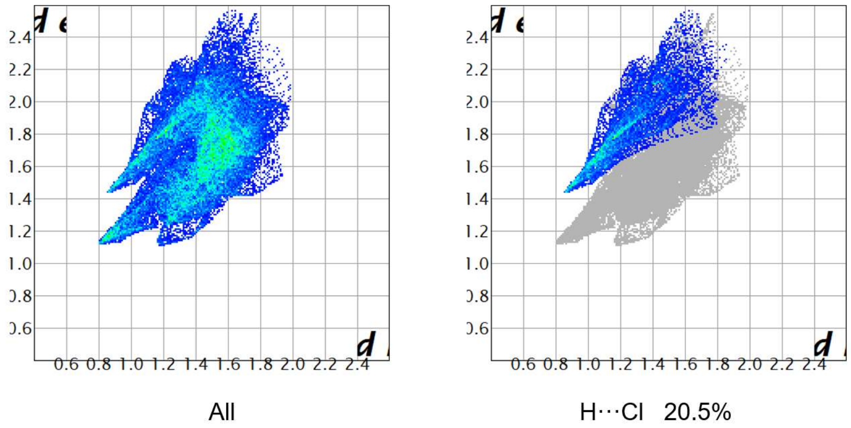

Figure S4. The selected fingerprint plots (d_e *vs*. d_i, Å) for $[Ni(en)_3]^{2+}$ cation in **1**.

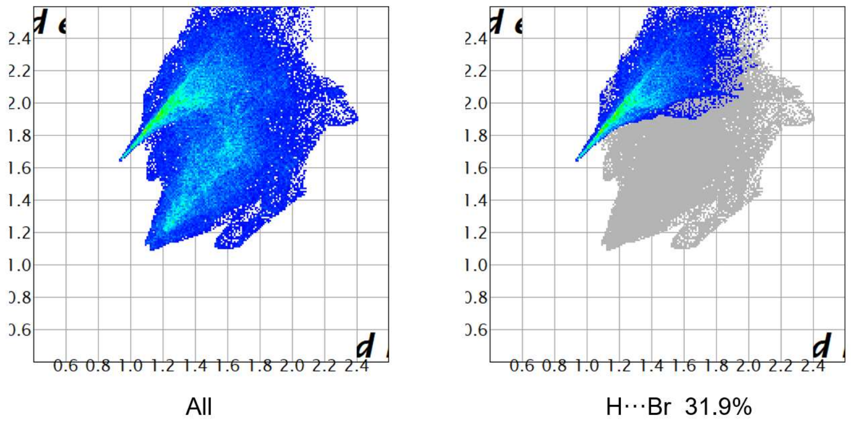

Figure S5. The selected fingerprint plots (d_e *vs*. d_i, Å) for $[Ni(en)_2(dmf)_2]^{2+}$ cation in 2.

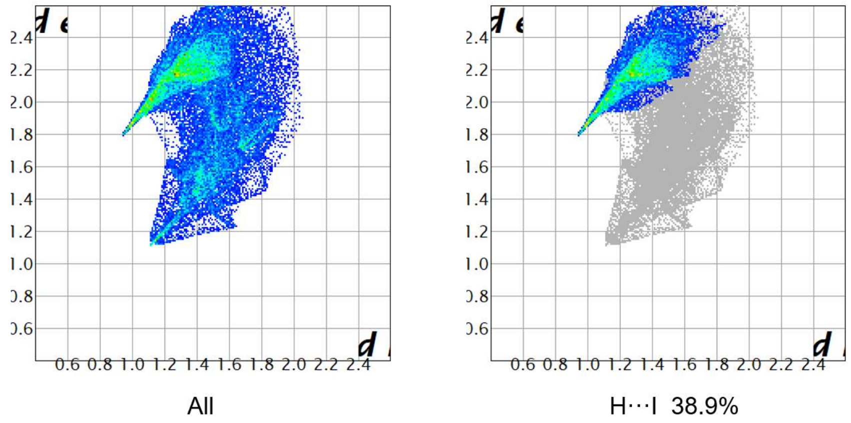

Figure S6. The selected fingerprint plots (d_e *vs*. d_i, Å) for $[Ni(en)_3]^{2+}$ cation in 3.

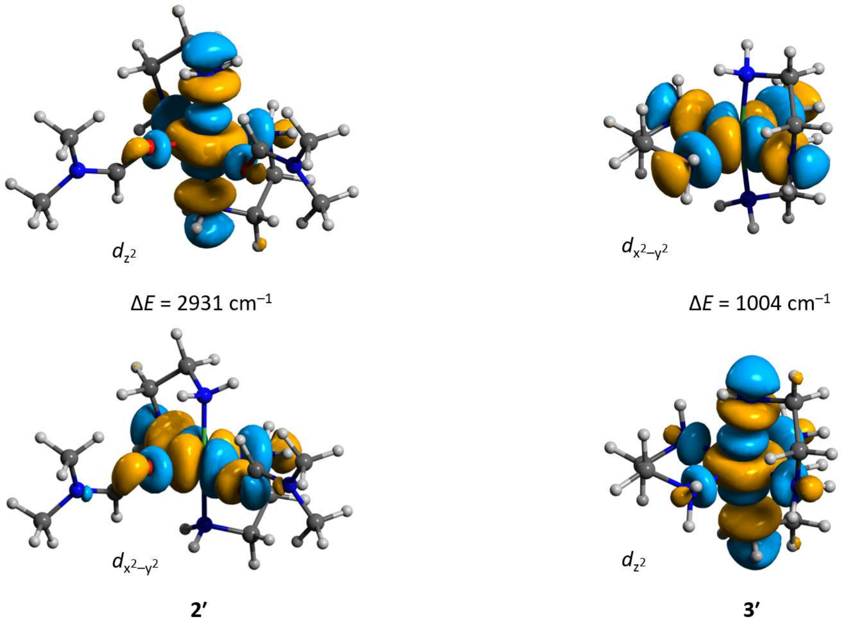

Figure S7. Isosurfaces of the singly occupied molecular orbitals (SOMOs) for **2'** (left) and **3'** (right) showing the energy gaps, calculated at the open-shell spin restricted PBE0/ZORA-def2-TZVPP level.

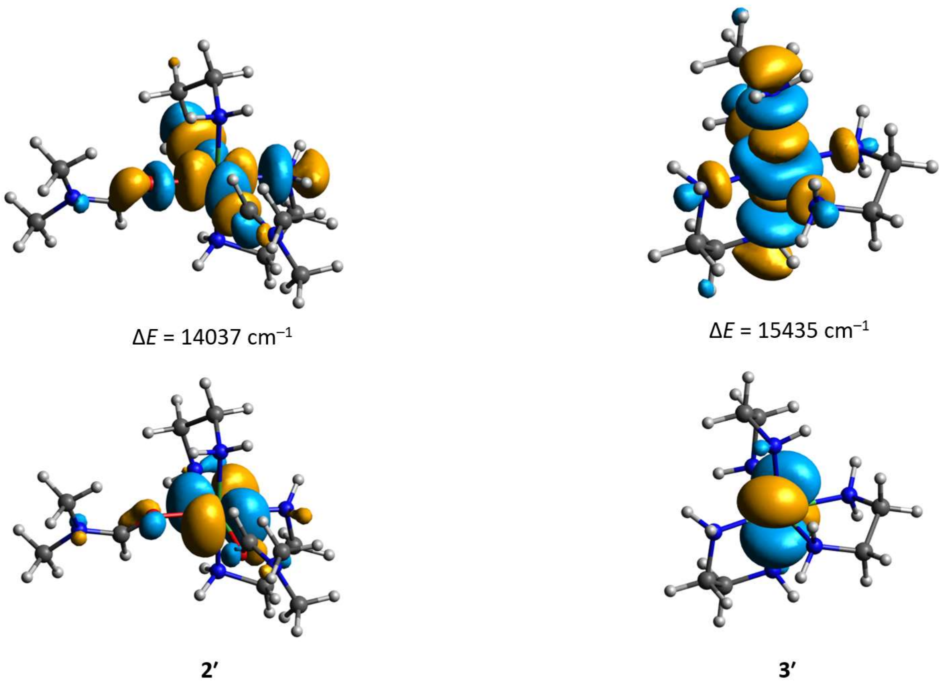

Figure S8. Isosurfaces of the natural transition orbitals (NTOs) for **2'** (top) and **3'** (bottom) involved into the first excited state transitions (100% contribution for **2'** and 80% for **3'**), calculated at the TDDFT PBE0/ZORA-def2-TZVPP level.

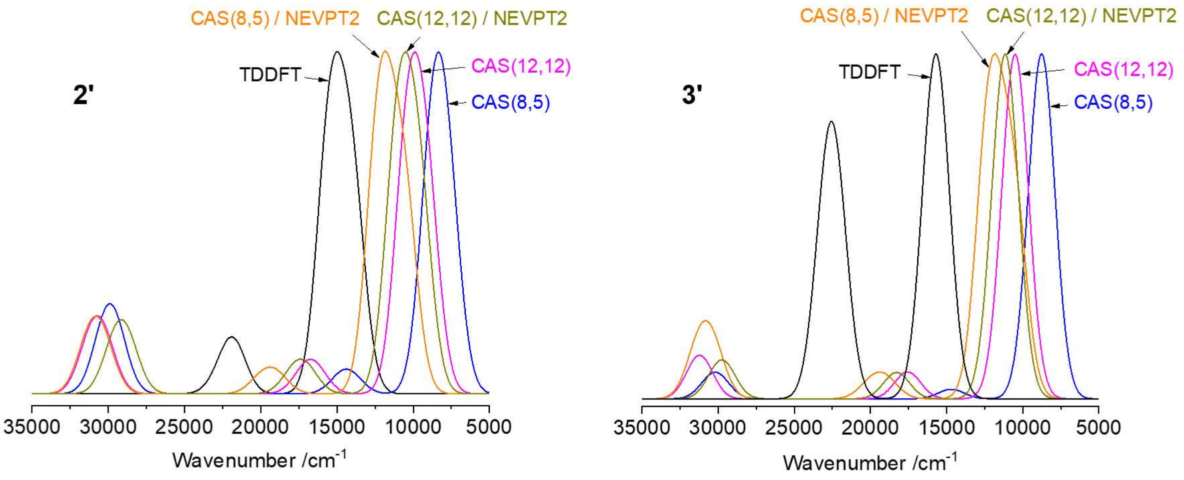

Figure S9. Fragments of the absorption spectra for **2**' (left) and **3**' (right) calculated at different levels of theory. The theoretical curves were obtained by the Gaussian broadening of the discrete transitions.

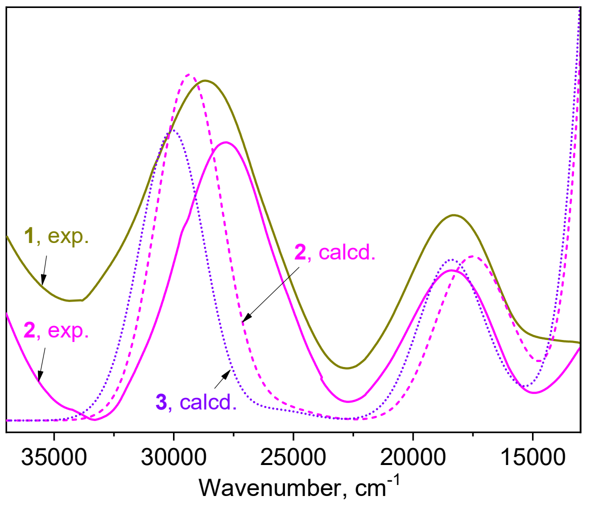

Figure S10. Experimental diffuse reflectance Vis spectra of **1** and **2** along with the theoretical ones for $[Ni(en)_2(dmf)_2]^{2+}$ (**2**') and $[Ni(en)_3]^{2+}$ (**3**') calculated at the CAS(12,12)/NEVPT2 level. The theoretical curves were obtained by the Gaussian broadening of the discrete transitions.

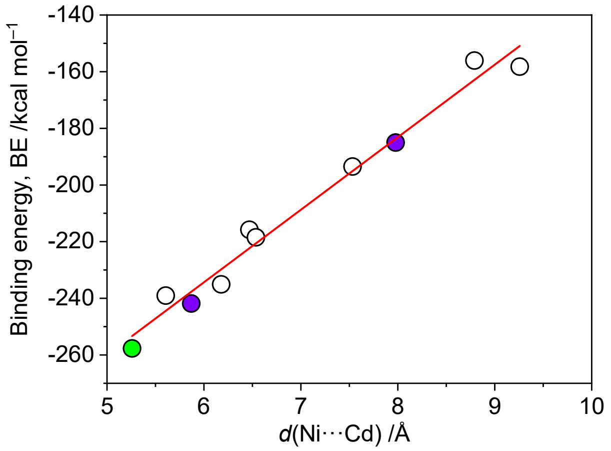

Figure S11. Dependence of the binding energy (BE) on the distance between the nickel and cadmium centres in the $\{Ni\}^{2+}\{Cd\}^{2-}$ supramolecular assembly (Table S5), where $\{Cd\}^{2-}$ stands for $[CdCl_4]^{2-}$ (green circle), $[CdI_4]^{2-}$ (violet circles) and $[CdBr_4]^{2-}$ (empty circles). Solid red line is a linear fit of these data.

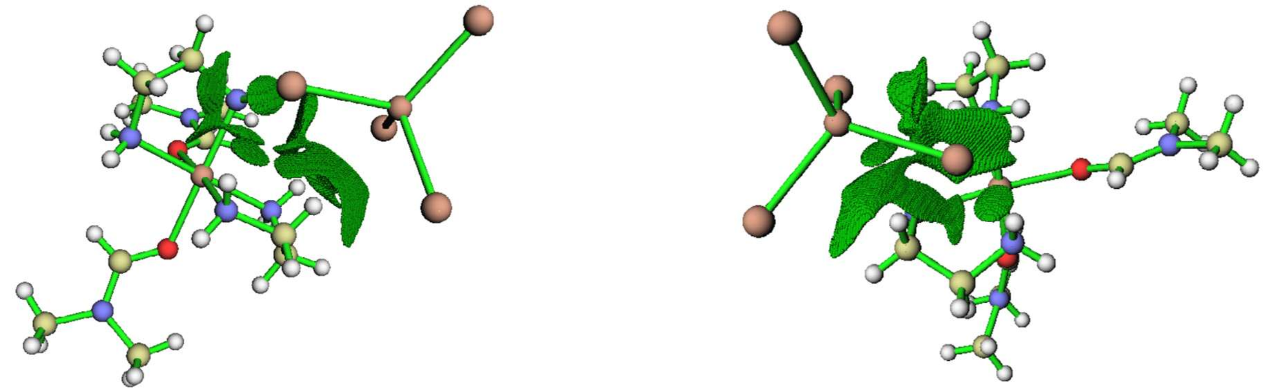

Figure S12. Non-covalent interactions domain calculated using the reduced density gradient in the $\{[Ni(en)_2(dmf)_2]^{2+}\cdots[CdBr_4]^{2-}\}$ assembly in **2**, where the symmetry operation for the cadmium centre is *x, 1 + y, z*. Left and right pictures show the same structure from different sides.

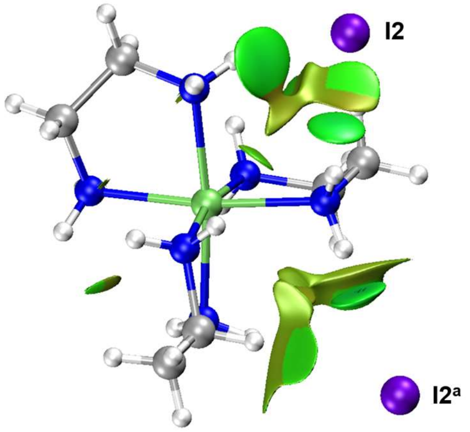

Figure S13. 3D isosurface of the RDG (isovalue of 0.5) illustrating non-covalent interactions in $\{I\cdots[Ni(en)_3]\cdots I^a\}$ fragment (^{a} = 1 + *y*, 1 – *x*, 1 – *z*).

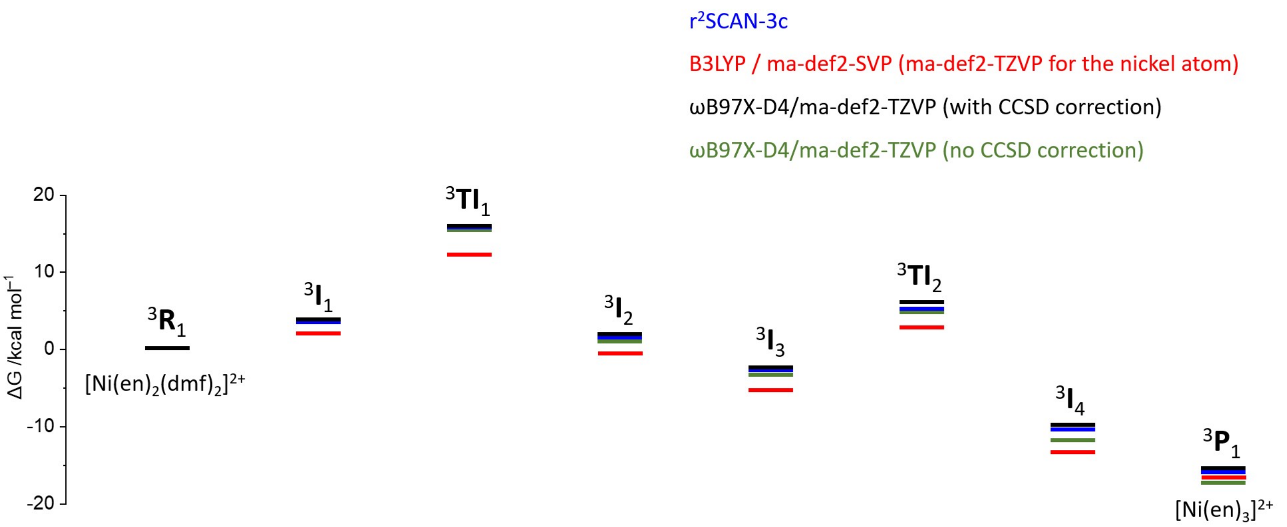

Figure S14. Free energy profiles of a reaction pathway for the ligand substitution in $[Ni(en)_3(dmf)_2]^{2+}$ (^{3}**R**$_1$) with formation of $[Ni(en)_3]^{2+}$ (^{3}**P**$_1$), calculated at the indicated levels of theory and involving the C-PCM dmf solvation model.

Table S1. Selected geometrical parameters (distances/Å and angles/°) for **1**.

Ni1–N12	2.066(9)	Cd1–Cl1	2.426(2)
Ni1–N11	2.084(9)	Cd1–Cl2	2.439(2)
Ni1–N21	2.103(9)	Cd1–Cl3	2.4430(18)
Ni1–N22	2.148(9)		
Ni1–N12'	2.156(9)		
Ni1–N11'	2.183(9)		
N12^1–Ni1–N12	107.7(5)	Cl1–Cd1–Cl2	113.83(8)
N12^1–Ni1–N11	133.3(4)	Cl1–Cd1–Cl3	107.68(6)
N12–Ni1–N11	83.4(4)	Cl2–Cd1–Cl3	108.97(5)
N21–Ni1–N22	81.8(3)		
N22–Ni1–N12'^{1}	115.0(3)		
N21–Ni1–N12'	168.9(4)		
N22–Ni1–N12'	90.9(4)		
N21–Ni1–N11'	92.1(4)		
N22–Ni1–N11'	89.2(3)		
N12'^{1}–Ni1–N11'	130.3(4)		
N12'–Ni1–N11'	79.4(4)		
N11'–Ni1–N11'^{1}	115.6(5)		

Symmetry transformation used to generate equivalent atoms: ^{1} x, –y+1/2, z.

Table S2. Selected geometrical parameters (distances/Å and angles/°) for **2**.

Ni1–O3	2.082(3)	Cd1–Br4	2.5595(6)
Ni1–N21	2.092(3)	Cd1–Br2	2.5950(6)
Ni1–O4	2.095(3)	Cd1–Br1	2.5950(6)
Ni1–N22	2.097(4)	Cd1–Br3	2.6207(5)
Ni1–N11	2.108(3)		
Ni1–N12	2.109(3)		
O3–Ni1–N21	84.19(14)	Br4–Cd1–Br2	108.40(2)
O3–Ni1–O4	90.34(13)	Br4–Cd1–Br1	113.651(18)
N21–Ni1–O4	95.89(13)	Br2–Cd1–Br1	111.197(19)
O3–Ni1–N22	89.63(14)	Br4–Cd1–Br3	112.33(2)
N21–Ni1–N22	83.59(14)	Br2–Cd1–Br3	102.160(18)
O4–Ni1–N22	179.48(14)	Br1–Cd1–Br3	108.521(18)
O3–Ni1–N11	97.59(13)		
N21–Ni1–N11	177.99(14)		
O4–Ni1–N11	85.06(13)		
N22–Ni1–N11	95.46(14)		
O3–Ni1–N12	175.59(14)		
N21–Ni1–N12	95.62(14)		
O4–Ni1–N12	85.29(14)		
N22–Ni1–N12	94.72(14)		
N11–Ni1–N12	82.68(14)		

Table S3. Selected geometrical parameters (distances/Å and angles/°) for **3**.

Ni1–N2	2.115(2)	Cd1–I1	2.7975(2)
Ni1–N3	2.125(2)		
Ni1–N1	2.149(3)		
N2–Ni1–N2[3]	95.59(13)	I1[1]–Cd1–I1	109.142(5)
N2–Ni1–N3[3]	172.07(10)	I1[2]–Cd1–I1	110.132(10)
N2–Ni1–N3	91.49(9)		
N3[3]–Ni1–N3	81.67(13)		
N2–Ni1–N1[3]	90.93(10)		
N3–Ni1–N1[3]	94.35(10)		
N2–Ni1–N1	82.01(10)		
N3–Ni1–N1	93.57(10)		
N1[3]–Ni1–N1	169.52(13)		

Symmetry transformations used to generate equivalent atoms: [1] 1/2–y, x–1/2, 3/2–z; [2] 1–x, –y, z; [3] x, 1/2–y, 5/4–z.

Table S4. Hydrogen bonding distances (Å) and angles (°) for complexes **1a–3**.

D–H···A	D–H	H···A	D···A	D–H···A
**1a**				
N11–H11A···O4[1]	0.91	2.02	2.905(12)	164.3
N11–H11B···Cl3	0.91	2.62	3.456(10)	153.6
N11'–H11C···Cl3	0.91	2.39	3.257(9)	159.3
N11'–H11D···O3[2]	0.91	2.04	2.930(10)	164.7
N12–H12E···O3[2]	0.91	2.29	3.135(10)	154.0
N12–H12F···Cl2[2]	0.91	2.81	3.658(9)	155.2
N12'–H12G···Cl2[3]	0.91	2.45	3.344(10)	166.1
N12'–H12H···O4[4]	0.91	2.11	2.987(12)	161.9
N21–H21A···Cl1	0.91	2.74	3.521(9)	144.8
N21–H21B···O3[3]	0.91	2.10	2.981(10)	161.6
N22–H22D···O3[2]	0.91	2.05	2.920(11)	158.9
**2**				
N11–H11A···Br1[1]	0.91	2.91	3.742(4)	152.4
N11–H11B···Br1[2]	0.91	2.81	3.645(4)	153.0
N12–H12C···Br3[2]	0.91	2.81	3.616(4)	149.1
N21–H21B···Br2[3]	0.91	2.68	3.583(4)	171.8
N22–H22C···Br3[2]	0.91	2.71	3.570(4)	158.2
N22–H22D···Br4[4]	0.91	2.70	3.529(4)	152.4
**3**				
N1–H1A···I1[1]	0.91	3.21	3.972	142.3
N1–H1B···I1	0.91	2.93	3.775(3)	154.8
N2–H2A···I2	0.91	2.91	3.728(2)	150.8
N2–H2B···I2[1]	0.91	2.84	3.708(2)	160.3
N3–H3A···I1[2]	0.91	3.07	3.875	149.3

Symmetry transformations used to generate equivalent atoms: **1a** [1] 1–x, 1–y, –z; [2] 1/2+x, y, 1/2–z; [3] 1/2+x, 1/2–y, 1/2–z; [4] 1–x, –1/2+y, –z; **2** [1] 1–x, 1–y, 1–z; [2] x, y+1,z; [3] 2–x, 1–y, 1–z; [4] 3/2–x, y+1/2, 3/2–z; **3** [1] 1+y, –1/2+x, 1/4+z; [2] 1/2–y, 1–x, –1/4+z.

Table S5. Binding energies of the selected blocks in **1–3** calculated at the ωB97X-D4/ma-def2-QZVPP level.

	Components			Separation		
Complex	A	B	*Symmetry operation for B*	Type	Distance /Å	Binding energy /kcal mol^{-1}
**1**	$[Ni(en)_3]^{2+}$ [a]	$[CdCl_4]^{2-}$	*x, y, z* [b]	*d*(Ni···Cd)	5.259	–257.70
	$[Ni(en)_3]^{2+}$ [a]	dmso	*1 – x, y – 0.5, –z* [c]	*d*(Ni···O)	4.033	–33.45
	$[Ni(en)_3]^{2+}$ [a]	dmso	*0.5 + x, y, 0.5 – z* [c]	*d*(Ni···O)	3.962	–33.47
	$[Ni(en)_3]^{2+}$ [d]	$[CdCl_4]^{2-}$	*x, y, z* [b]	*d*(Ni···Cd)	5.529	–257.70
	$[Ni(en)_3]^{2+}$ [d]	dmso	*1 – x, y – 0.5, –z* [c]	*d*(Ni···O)	4.033	–28.86
	$[Ni(en)_3]^{2+}$ [d]	dmso	*0.5 + x, y, 0.5 – z* [c]	*d*(Ni···O)	3.962	–33.45
**2**	$[Ni(en)_2(dmf)_2]^{2+}$	$[CdBr_4]^{2-}$	*x, y, z* [b]	*d*(Ni···Cd)	6.469	–215.81
	$[Ni(en)_2(dmf)_2]^{2+}$	$[CdBr_4]^{2-}$	*x – 0.5, 1.5 – y, z –0.5* [b]	*d*(Ni···Cd)	8.791	–156.09
	$[Ni(en)_2(dmf)_2]^{2+}$	$[CdBr_4]^{2-}$	*x, 1 + y, z* [b]	*d*(Ni···Cd)	5.606	–239.04
	$[Ni(en)_2(dmf)_2]^{2+}$	$[CdBr_4]^{2-}$	*2 – x, 1 – y, 1 – z* [b]	*d*(Ni···Cd)	6.178	–235.09
	$[Ni(en)_2(dmf)_2]^{2+}$	$[CdBr_4]^{2-}$	*1 – x, 1 – y, 1 – z* [b]	*d*(Ni···Cd)	7.533	–193.47
	$[Ni(en)_2(dmf)_2]^{2+}$	$[CdBr_4]^{2-}$	*x – 0.5, 0.5 – y, z – 0.5* [b]	*d*(Ni···Cd)	9.258	–158.22
	$[Ni(en)_2(dmf)_2]^{2+}$	$[CdBr_4]^{2-}$	*1.5 – x, 0.5 + y, 1.5 – z* [b]	*d*(Ni···Cd)	6.536	–218.47
**3**	$[Ni(en)_3]^{2+}$	$[CdI_4]^{2-}$	*x, y, z* [b]	*d*(Ni···Cd)	5.869	–241.85
	$[Ni(en)_3]^{2+}$	$[CdI_4]^{2-}$	*1 – y, x, 1 – z* [b]	*d*(Ni···Cd)	7.978	–185.02
	$[Ni(en)_3]^{2+}$	I^-	*x, y, z* [e]	*d*(Ni···I)	4.725	–145.84
	$[Ni(en)_3]^{2+}$	I^-	*1 + y, 1 – x, 1 – z* [e]	*d*(Ni···I)	4.782	–149.60

[a] First disordered component; [b] symmetry operation for the central cadmium atom of the $[CdHal_4]^{2-}$ anion; [c] symmetry operation for the dmso molecule; [d] second disordered component; [e] symmetry operation for the iodine atom.

Table S6. Gibbs free energies (Hartree) of the molecular fragments involved in the ^{3}**R**$_1$ → ^{3}**P**$_1$ reaction.[a]

	ωB97X-D4[b]	ωB97X-D4[b] / CCSD[c]	B3LYP[d]	r^{2}SCAN-3c
dmf	–248.6408964	–248.050007	–248.1479228	–248.389304
en	–190.6101744	–190.1389362	–190.2099297	–190.3926915
^{3}**R**$_1$, $[Ni(en)_2(dmf)_2]^{2+}$	–2386.62267	–2383.746161	–2384.809812	–2385.718158
^{3}**P**$_1$, $[Ni(en)_3]^{2+}$	–2079.98239	–2077.813309	–2078.754177	–2079.361367
^{3}**I**$_1$	–2577.20477	–2573.876047	–2575.013564	–2576.102241
^{3}**TI**$_1$	–2577.22832	–2573.856077	–2574.996859	–2576.082169
^{3}**I**$_2$	–2328.597605	–2573.879129	–2575.017729	–2576.10564
^{3}**I**$_3$	–2328.584405	–2325.839064	–2326.880673	–2327.72606
^{3}**TI**$_2$	–2328.611335	–2325.825262	–2326.867395	–2327.713208
^{3}**I**$_4$	–2328.611335	–2325.851281	–2326.893631	–2327.738638

[a] Geometries optimized on the same DFT level. C-PCM dmf solvation model was applied in all cases; [b] ma-def2-TZVP basis set; [c] Correction of the electronic energy through the DLPNO-CCSD(T)/ma-def2-TZVPP calculations; [d] ma-def2-TZVP basis set for nickel atom and ma-def2-SVP for all other atoms.

Table S7. Maxima of the Gaussian lineshapes of the transitions (Listings S1–S4) calculated at the stated levels.[a]

Compound	Method	Wavenumbers /cm^{-1}		
**2'**				
	CAS(8,5)	8343	14413	29897
	CAS(8,5)/NEVPT2	11862	19384	30821
	CAS(12,12)	9883	16745	30733
	CAS(12,12)/NEVPT2	10543	11488	29326
	TD-DFT	14985	21982	
**3'**				
	CAS(8,5)	8739	14765	30205
	CAS(8,5)/NEVPT2	11862	19384	30821
	CAS(12,12)	10543	17581	31217
	CAS(12,12)/NEVPT2	11158	18377	30041
	TD-DFT	15689	22595	

[a] the 5000 – 30000 cm^{-1} range. The Gaussian lineshapes were calculated using the orca_mapspc tool from the ORCA package.

Listings S1. Selected output of the stated-averaged CAS(8,5)/AILFT(SC-NEVPT2) calculation for **2'**.

```
----------------------------
LOEWDIN REDUCED ACTIVE MOs
----------------------------

                  83
               -0.37121
                1.60000
               --------
 0 Ni dz2          92.9
 1 N  pz            1.2
 7 C  px            0.0
 8 H  s             0.0
10 N  px            0.3
13 N  pz            1.3
19 C  py            0.0
21 H  s             0.0
22 N  py            0.3
25 O  py            0.0
28 N  py            0.0
37 O  px            0.0
40 N  px            0.0

                  84        85        86        87
               -0.41493  -0.41497  -0.37960  -0.41469
                1.60000   1.60000   1.60000   1.60000
               --------  --------  --------  --------
 0 Ni s             0.0       0.0       0.0       0.0
 0 Ni dxz          98.7       0.0       0.0       0.0
 0 Ni dyz           0.0      98.7       0.0       0.0
 0 Ni dx2y2         0.0       0.0      93.9       0.0
 0 Ni dxy           0.0       0.0       0.0      98.7
12 H  s             0.0       0.0       0.0       0.1
24 H  s             0.0       0.0       0.0       0.0
44 H  s             0.0       0.0       0.0       0.0

---------------------------------------------
CAS-SCF STATES FOR BLOCK  1 MULT= 3 NROOTS=10
---------------------------------------------

ROOT   0:  E=   -2399.7422139472 Eh
      0.97263 [     2]: 12212
      0.01016 [     3]: 12221
      0.00694 [     7]: 22112
      0.00427 [     1]: 12122
      0.00339 [     5]: 21212
ROOT   1:  E=   -2399.7071356007 Eh  0.955 eV   7698.8 cm**-1
      0.97924 [     3]: 12221
      0.00958 [     2]: 12212
      0.00585 [     8]: 22121
      0.00272 [     0]: 11222
ROOT   2:  E=   -2399.7026033486 Eh  1.078 eV   8693.5 cm**-1
      0.59420 [     5]: 21212
      0.26308 [     0]: 11222
      0.10318 [     7]: 22112
      0.02264 [     1]: 12122
      0.00549 [     6]: 21221
      0.00510 [     4]: 21122
      0.00293 [     8]: 22121
ROOT   3:  E=   -2399.7023609626 Eh  1.084 eV   8746.7 cm**-1
      0.58360 [     7]: 22112
      0.26461 [     1]: 12122
      0.08940 [     5]: 21212
      0.02224 [     0]: 11222
      0.01564 [     2]: 12212
      0.01374 [     4]: 21122
      0.00414 [     8]: 22121
      0.00342 [     6]: 21221
      0.00320 [     9]: 22211
ROOT   4:  E=   -2399.6786666787 Eh  1.729 eV  13947.0 cm**-1
      0.27760 [     6]: 21221
      0.22288 [     8]: 22121
      0.16938 [     1]: 12122
      0.09086 [     4]: 21122
      0.07928 [     9]: 22211
      0.07698 [     0]: 11222
      0.05032 [     5]: 21212
      0.03227 [     7]: 22112
ROOT   5:  E=   -2399.6768843972 Eh  1.778 eV  14338.2 cm**-1
      0.34526 [     8]: 22121
      0.22027 [     6]: 21221
      0.20132 [     0]: 11222
```

```
     0.07102 [     1]: 12122
     0.06792 [     5]: 21212
     0.06366 [     7]: 22112
     0.01461 [     4]: 21122
     0.00901 [     3]: 12221
     0.00669 [     9]: 22211
ROOT   6:  E=   -2399.6746378096 Eh  1.839 eV  14831.2 cm**-1
     0.48603 [     4]: 21122
     0.30058 [     9]: 22211
     0.08636 [     6]: 21221
     0.04683 [     7]: 22112
     0.03530 [     1]: 12122
     0.03342 [     5]: 21212
     0.01124 [     8]: 22121
ROOT   7:  E=   -2399.6081033787 Eh  3.649 eV  29433.9 cm**-1
     0.30176 [     1]: 12122
     0.25444 [     6]: 21221
     0.11186 [     7]: 22112
     0.09942 [     9]: 22211
     0.08170 [     4]: 21122
     0.06962 [     0]: 11222
     0.06458 [     8]: 22121
     0.01657 [     5]: 21212
ROOT   8:  E=   -2399.6076759953 Eh  3.661 eV  29527.7 cm**-1
     0.34952 [     0]: 11222
     0.31767 [     8]: 22121
     0.13754 [     5]: 21212
     0.08941 [     6]: 21221
     0.08069 [     1]: 12122
     0.02366 [     7]: 22112
ROOT   9:  E=   -2399.6042237223 Eh  3.755 eV  30285.4 cm**-1
     0.50964 [     9]: 22211
     0.30702 [     4]: 21122
     0.06093 [     6]: 21221
     0.05014 [     1]: 12122
     0.02768 [     7]: 22112
     0.02546 [     8]: 22121
     0.01197 [     0]: 11222
     0.00714 [     5]: 21212

---------------------------------------------
CAS-SCF STATES FOR BLOCK  2 MULT= 1 NROOTS=15
---------------------------------------------

ROOT   0:  E=   -2399.6586898562 Eh
     0.55024 [     0]: 02222
     0.37721 [    12]: 22202
     0.04173 [    14]: 22220
     0.00570 [     5]: 20222
     0.00552 [     2]: 12122
     0.00441 [    10]: 22112
     0.00440 [     9]: 22022
     0.00351 [    13]: 22211
     0.00278 [     7]: 21212
     0.00265 [     1]: 11222
ROOT   1:  E=   -2399.6583750613 Eh  0.009 eV     69.1 cm**-1
     0.92794 [     3]: 12212
     0.02674 [     9]: 22022
     0.02442 [     5]: 20222
     0.00942 [     4]: 12221
     0.00383 [    10]: 22112
     0.00257 [     2]: 12122
ROOT   2:  E=   -2399.6263898683 Eh  0.879 eV   7089.0 cm**-1
     0.85699 [     4]: 12221
     0.11911 [     6]: 21122
     0.00793 [     3]: 12212
     0.00642 [    10]: 22112
     0.00292 [     1]: 11222
     0.00253 [     2]: 12122
ROOT   3:  E=   -2399.6224688608 Eh  0.986 eV   7949.6 cm**-1
     0.46060 [     7]: 21212
     0.21818 [     1]: 11222
     0.10112 [    10]: 22112
     0.09632 [    11]: 22121
     0.07184 [     2]: 12122
     0.04685 [     8]: 21221
ROOT   4:  E=   -2399.6221341056 Eh  0.995 eV   8023.1 cm**-1
     0.46274 [    10]: 22112
     0.20449 [     2]: 12122
     0.10829 [     7]: 21212
     0.09354 [     8]: 21221
     0.05361 [     1]: 11222
     0.04421 [    11]: 22121
     0.01369 [     4]: 12221
     0.01233 [     0]: 02222
     0.00303 [    14]: 22220
```

```
ROOT   5:  E=   -2399.6068270586 Eh  1.411 eV  11382.6 cm**-1
      0.45145 [    12]: 22202
      0.29629 [     0]: 02222
      0.08055 [     5]: 20222
      0.07464 [     9]: 22022
      0.04997 [    14]: 22220
      0.01611 [    13]: 22211
      0.01377 [     2]: 12122
      0.01277 [     1]: 11222
      0.00342 [    10]: 22112
ROOT   6:  E=   -2399.5941201959 Eh  1.757 eV  14171.4 cm**-1
      0.66955 [     1]: 11222
      0.29506 [     7]: 21212
      0.01203 [     5]: 20222
      0.00643 [     3]: 12212
      0.00396 [     0]: 02222
      0.00301 [    11]: 22121
      0.00299 [     8]: 21221
      0.00299 [     2]: 12122
ROOT   7:  E=   -2399.5938077482 Eh  1.766 eV  14240.0 cm**-1
      0.65697 [     2]: 12122
      0.28440 [    10]: 22112
      0.02234 [     9]: 22022
      0.01020 [     0]: 02222
      0.00935 [     3]: 12212
      0.00388 [     8]: 21221
      0.00386 [     1]: 11222
      0.00358 [    13]: 22211
      0.00251 [    11]: 22121
ROOT   8:  E=   -2399.5905978207 Eh  1.853 eV  14944.5 cm**-1
      0.93742 [    13]: 22211
      0.01996 [    12]: 22202
      0.01919 [    14]: 22220
      0.00796 [    11]: 22121
      0.00577 [     7]: 21212
      0.00536 [    10]: 22112
      0.00374 [     8]: 21221
ROOT   9:  E=   -2399.5674552535 Eh  2.483 eV  20023.7 cm**-1
      0.57741 [    14]: 22220
      0.22698 [     5]: 20222
      0.06717 [     9]: 22022
      0.02767 [    11]: 22121
      0.02511 [     8]: 21221
      0.02400 [    12]: 22202
      0.01892 [     0]: 02222
      0.01821 [    13]: 22211
      0.00539 [     1]: 11222
      0.00302 [     2]: 12122
ROOT  10:  E=   -2399.5665845047 Eh  2.506 eV  20214.8 cm**-1
      0.51285 [     9]: 22022
      0.35423 [     5]: 20222
      0.04193 [     3]: 12212
      0.02106 [     8]: 21221
      0.01962 [    11]: 22121
      0.01793 [    14]: 22220
      0.01733 [     2]: 12122
      0.00789 [     6]: 21122
      0.00405 [     1]: 11222
ROOT  11:  E=   -2399.5644954978 Eh  2.563 eV  20673.3 cm**-1
      0.51815 [     6]: 21122
      0.25377 [     8]: 21221
      0.08637 [     4]: 12221
      0.07067 [    11]: 22121
      0.03227 [    10]: 22112
      0.01956 [    14]: 22220
      0.01117 [     5]: 20222
ROOT  12:  E=   -2399.5634853459 Eh  2.591 eV  20895.0 cm**-1
      0.48321 [    11]: 22121
      0.29788 [     8]: 21221
      0.09381 [     7]: 21212
      0.02495 [    10]: 22112
      0.02360 [     9]: 22022
      0.02191 [     5]: 20222
      0.01632 [    13]: 22211
      0.01382 [     6]: 21122
      0.00964 [     2]: 12122
      0.00820 [     1]: 11222
      0.00421 [     4]: 12221
ROOT  13:  E=   -2399.5630158071 Eh  2.603 eV  20998.0 cm**-1
      0.33497 [     6]: 21122
      0.25055 [     8]: 21221
      0.24342 [    11]: 22121
      0.06892 [    10]: 22112
      0.02845 [     7]: 21212
      0.02569 [     4]: 12221
      0.01666 [     1]: 11222
```

```
      0.01546 [    14]: 22220
      0.00647 [     2]: 12122
      0.00510 [     9]: 22022
ROOT  14:  E=   -2399.4139401295 Eh  6.660 eV  53716.4 cm**-1
      0.26152 [     9]: 22022
      0.26102 [     5]: 20222
      0.25449 [    14]: 22220
      0.11705 [    12]: 22202
      0.10418 [     0]: 02222
```

SA-CASSCF TRANSITION ENERGIES

LOWEST ROOT (ROOT 0 ,MULT 3) = -2399.742213947 Eh -65300.305 eV

STATE	ROOT	MULT	DE/a.u.	DE/eV	DE/cm**-1
1:	1	3	0.035078	0.955	7698.8
2:	2	3	0.039611	1.078	8693.5
3:	3	3	0.039853	1.084	8746.7
4:	4	3	0.063547	1.729	13947.0
5:	5	3	0.065330	1.778	14338.2
6:	6	3	0.067576	1.839	14831.2
7:	0	1	0.083524	2.273	18331.4
8:	1	1	0.083839	2.281	18400.5
9:	2	1	0.115824	3.152	25420.4
10:	3	1	0.119745	3.258	26281.0
11:	4	1	0.120080	3.268	26354.5
12:	7	3	0.134111	3.649	29433.9
13:	8	3	0.134538	3.661	29527.7
14:	5	1	0.135387	3.684	29714.0
15:	9	3	0.137990	3.755	30285.4
16:	6	1	0.148094	4.030	32502.8
17:	7	1	0.148406	4.038	32571.4
18:	8	1	0.151616	4.126	33275.9
19:	9	1	0.174759	4.755	38355.1
20:	10	1	0.175629	4.779	38546.2
21:	11	1	0.177718	4.836	39004.7
22:	12	1	0.178729	4.863	39226.4
23:	13	1	0.179198	4.876	39329.4
24:	14	1	0.328274	8.933	72047.8

NEVPT2 TRANSITION ENERGIES

LOWEST ROOT (ROOT 0, MULT 3) = -2403.920989992 Eh -65414.016 eV

STATE	ROOT	MULT	DE/a.u.	DE/eV	DE/cm**-1
1:	1	3	0.048468	1.319	10637.6
2:	2	3	0.055354	1.506	12148.7
3:	3	3	0.055641	1.514	12211.9
4:	0	1	0.072167	1.964	15838.7
5:	1	1	0.072915	1.984	16003.0
6:	4	3	0.085662	2.331	18800.7
7:	5	3	0.088048	2.396	19324.4
8:	6	3	0.091404	2.487	20060.8
9:	2	1	0.119615	3.255	26252.4
10:	5	1	0.125114	3.405	27459.4
11:	3	1	0.125767	3.422	27602.8
12:	4	1	0.126069	3.431	27669.0
13:	7	3	0.137997	3.755	30286.9
14:	8	3	0.138463	3.768	30389.1
15:	9	3	0.142565	3.879	31289.3
16:	6	1	0.147700	4.019	32416.5
17:	7	1	0.148088	4.030	32501.6
18:	8	1	0.152150	4.140	33393.0
19:	9	1	0.187695	5.107	41194.3
20:	10	1	0.189312	5.151	41549.3
21:	11	1	0.190550	5.185	41821.0
22:	12	1	0.191068	5.199	41934.7
23:	13	1	0.191781	5.219	42091.0
24:	14	1	0.308742	8.401	67761.0

Racah Parameters :

```
B                    =  0.005418194 a.u. =     0.147 eV =    1189.2 cm**-1
C                    =  0.017338559 a.u. =     0.472 eV =    3805.4 cm**-1
C/B                  =  3.200
------------------------------------------------------------------------------
```

The ligand field one electron eigenfunctions:

Orbital	Energy (eV)	Energy(cm-1)	dz2	dxz	dyz	dx2-y2	dxy
1	0.000	0.0	0.089485	-0.481544	0.870846	0.032218	0.026393
2	0.011	92.5	-0.002289	0.859215	0.475655	0.114344	-0.149737
3	0.038	307.3	-0.010726	0.150588	0.057684	-0.084126	0.983262
4	1.180	9518.8	-0.003413	0.070490	0.078964	-0.989324	-0.100110
5	1.344	10843.0	0.995922	0.047106	-0.076262	-0.006929	0.007531

D = -5.662736 cm-1
E/D = 0.052761

g-factors:
2.256065 2.258721 2.298415 iso = 2.271067

Listings S2. Selected output of the stated-averaged CAS(8,5)/AILFT(SC-NEVPT2) calculation for **3'**.

```
----------------------------
LOEWDIN REDUCED ACTIVE MOs
----------------------------

                   60        61        62        63        64
               -0.37426  -0.41433  -0.41338  -0.37105  -0.41316
                1.60000   1.60000   1.60000   1.60000   1.60000
               --------  --------  --------  --------  --------
 0 Ni dz2          93.3       0.0       0.0       0.0       0.0
 0 Ni dxz           0.0      98.7       0.0       0.0       0.0
 0 Ni dyz           0.0       0.0      98.6       0.0       0.0
 0 Ni dx2y2         0.0       0.0       0.0      92.9       0.0
 0 Ni dxy           0.0       0.0       0.0       0.0      98.6

---------------------------------------------
CAS-SCF STATES FOR BLOCK  1 MULT= 3 NROOTS=10
---------------------------------------------

ROOT   0:  E=   -2094.6369082146 Eh
      0.94240 [     2]: 12212
      0.02044 [     7]: 22112
      0.02019 [     3]: 12221
      0.00894 [     1]: 12122
      0.00618 [     5]: 21212
ROOT   1:  E=   -2094.5979462650 Eh  1.060 eV   8551.2 cm**-1
      0.43504 [     7]: 22112
      0.31663 [     5]: 21212
      0.12431 [     0]: 11222
      0.09641 [     1]: 12122
      0.00854 [     6]: 21221
      0.00628 [     8]: 22121
      0.00469 [     4]: 21122
      0.00398 [     2]: 12212
      0.00258 [     3]: 12221
ROOT   2:  E=   -2094.5975790161 Eh  1.070 eV   8631.8 cm**-1
      0.40532 [     5]: 21212
      0.29070 [     7]: 22112
      0.12365 [     1]: 12122
      0.08370 [     0]: 11222
      0.03772 [     2]: 12212
      0.02278 [     4]: 21122
      0.01400 [     8]: 22121
      0.00832 [     3]: 12221
      0.00792 [     6]: 21221
      0.00588 [     9]: 22211
ROOT   3:  E=   -2094.5959056035 Eh  1.116 eV   8999.0 cm**-1
      0.93559 [     3]: 12221
      0.01571 [     8]: 22121
      0.01519 [     2]: 12212
      0.01362 [     0]: 11222
      0.00695 [     5]: 21212
      0.00641 [     7]: 22112
      0.00572 [     6]: 21221
ROOT   4:  E=   -2094.5700367852 Eh  1.820 eV  14676.6 cm**-1
      0.36684 [     4]: 21122
      0.30016 [     9]: 22211
      0.10874 [     8]: 22121
      0.09655 [     0]: 11222
      0.05817 [     6]: 21221
      0.03796 [     7]: 22112
      0.01912 [     1]: 12122
      0.00904 [     3]: 12221
      0.00311 [     5]: 21212
ROOT   5:  E=   -2094.5686887112 Eh  1.856 eV  14972.5 cm**-1
      0.35066 [     8]: 22121
      0.21516 [     6]: 21221
```

```
      0.18108 [     1]: 12122
      0.12751 [     0]: 11222
      0.09171 [     5]: 21212
      0.01674 [     4]: 21122
      0.01279 [     7]: 22112
ROOT   6:  E=   -2094.5684780296 Eh  1.862 eV  15018.7 cm**-1
      0.29977 [     6]: 21221
      0.16691 [     4]: 21122
      0.12061 [     1]: 12122
      0.11368 [     9]: 22211
      0.10741 [     8]: 22121
      0.09463 [     0]: 11222
      0.05051 [     7]: 22112
      0.02623 [     5]: 21212
      0.02013 [     3]: 12221
ROOT   7:  E=   -2094.5004991150 Eh  3.712 eV  29938.3 cm**-1
      0.55386 [     9]: 22211
      0.41501 [     4]: 21122
      0.02309 [     0]: 11222
      0.00354 [     8]: 22121
ROOT   8:  E=   -2094.4996798176 Eh  3.734 eV  30118.2 cm**-1
      0.25608 [     1]: 12122
      0.20633 [     6]: 21221
      0.19159 [     8]: 22121
      0.19080 [     0]: 11222
      0.09840 [     7]: 22112
      0.04811 [     5]: 21212
      0.00833 [     9]: 22211
ROOT   9:  E=   -2094.4985117068 Eh  3.766 eV  30374.5 cm**-1
      0.24492 [     0]: 11222
      0.20153 [     8]: 22121
      0.19601 [     6]: 21221
      0.19410 [     1]: 12122
      0.09393 [     5]: 21212
      0.04732 [     7]: 22112
      0.01450 [     9]: 22211
      0.00610 [     4]: 21122

---------------------------------------------
CAS-SCF STATES FOR BLOCK  2 MULT= 1 NROOTS=15
---------------------------------------------

ROOT   0:  E=   -2094.5535332497 Eh
      0.49295 [    12]: 22202
      0.41245 [     0]: 02222
      0.03025 [    14]: 22220
      0.01287 [     2]: 12122
      0.01246 [    13]: 22211
      0.01185 [    10]: 22112
      0.01024 [     9]: 22022
      0.00951 [     5]: 20222
      0.00404 [     1]: 11222
ROOT   1:  E=   -2094.5533118911 Eh  0.006 eV     48.6 cm**-1
      0.90595 [     3]: 12212
      0.02683 [     9]: 22022
      0.02276 [     5]: 20222
      0.01949 [     4]: 12221
      0.01462 [    10]: 22112
      0.00442 [     7]: 21212
      0.00351 [     2]: 12122
ROOT   2:  E=   -2094.5176905077 Eh  0.975 eV   7866.6 cm**-1
      0.33365 [    10]: 22112
      0.32157 [     7]: 21212
      0.09962 [    11]: 22121
      0.09905 [     1]: 11222
      0.07595 [     2]: 12122
      0.03135 [     8]: 21221
      0.01268 [    12]: 22202
      0.01208 [     6]: 21122
      0.00906 [     0]: 02222
ROOT   3:  E=   -2094.5171931782 Eh  0.989 eV   7975.7 cm**-1
      0.33737 [     7]: 21212
      0.32189 [    10]: 22112
      0.10751 [     2]: 12122
      0.09631 [     8]: 21221
      0.08274 [     1]: 11222
      0.03207 [    11]: 22121
      0.00915 [     3]: 12212
      0.00383 [     6]: 21122
      0.00289 [     0]: 02222
ROOT   4:  E=   -2094.5157322218 Eh  1.029 eV   8296.4 cm**-1
      0.83562 [     4]: 12221
      0.12387 [     6]: 21122
      0.01736 [     3]: 12212
      0.01280 [    11]: 22121
      0.00399 [     7]: 21212
```

ROOT 5: E= -2094.5019597750 Eh 1.403 eV 11319.1 cm**-1
0.39921 [0]: 02222
0.32544 [12]: 22202
0.07121 [14]: 22220
0.06087 [5]: 20222
0.05189 [9]: 22022
0.03705 [2]: 12122
0.03224 [13]: 22211
0.00923 [10]: 22112
0.00896 [1]: 11222
0.00297 [7]: 21212
ROOT 6: E= -2094.4884074819 Eh 1.772 eV 14293.5 cm**-1
0.89759 [13]: 22211
0.04094 [12]: 22202
0.04041 [14]: 22220
0.00953 [11]: 22121
0.00815 [7]: 21212
ROOT 7: E= -2094.4858908928 Eh 1.841 eV 14845.8 cm**-1
0.41259 [2]: 12122
0.31748 [1]: 11222
0.11542 [7]: 21212
0.08045 [10]: 22112
0.03229 [9]: 22022
0.02365 [3]: 12212
0.00635 [5]: 20222
0.00497 [0]: 02222
ROOT 8: E= -2094.4855973761 Eh 1.849 eV 14910.2 cm**-1
0.41960 [1]: 11222
0.27015 [2]: 12122
0.11489 [10]: 22112
0.09753 [7]: 21212
0.03606 [0]: 02222
0.02441 [9]: 22022
0.00924 [6]: 21122
0.00781 [5]: 20222
0.00746 [8]: 21221
0.00629 [4]: 12221
0.00296 [12]: 22202
ROOT 9: E= -2094.4592821765 Eh 2.565 eV 20685.7 cm**-1
0.57168 [14]: 22220
0.16056 [9]: 22022
0.13747 [5]: 20222
0.03520 [13]: 22211
0.02178 [0]: 02222
0.01931 [6]: 21122
0.01794 [8]: 21221
0.01556 [12]: 22202
0.01075 [2]: 12122
0.00305 [4]: 12221
ROOT 10: E= -2094.4587306517 Eh 2.580 eV 20806.8 cm**-1
0.45202 [5]: 20222
0.42907 [9]: 22022
0.03645 [3]: 12212
0.02647 [2]: 12122
0.02077 [6]: 21122
0.01396 [10]: 22112
0.00756 [8]: 21221
0.00511 [1]: 11222
0.00444 [7]: 21212
0.00290 [11]: 22121
ROOT 11: E= -2094.4565457639 Eh 2.639 eV 21286.3 cm**-1
0.52537 [11]: 22121
0.31757 [8]: 21221
0.09193 [7]: 21212
0.01804 [2]: 12122
0.01432 [13]: 22211
0.01418 [1]: 11222
0.00657 [10]: 22112
0.00613 [5]: 20222
ROOT 12: E= -2094.4557966730 Eh 2.660 eV 21450.7 cm**-1
0.50064 [8]: 21221
0.30301 [11]: 22121
0.08672 [10]: 22112
0.02467 [4]: 12221
0.01604 [14]: 22220
0.01594 [6]: 21122
0.01504 [1]: 11222
0.01393 [2]: 12122
0.01257 [5]: 20222
0.00595 [7]: 21212
ROOT 13: E= -2094.4555104460 Eh 2.667 eV 21513.5 cm**-1
0.79234 [6]: 21122
0.10573 [4]: 12221
0.03040 [1]: 11222
0.01840 [5]: 20222
0.01522 [8]: 21221

0.00919 [11]: 22121
0.00893 [14]: 22220
0.00794 [2]: 12122
0.00338 [7]: 21212
0.00278 [10]: 22112
ROOT 14: E= -2094.3072978087 Eh 6.700 eV 54042.4 cm**-1
0.26284 [5]: 20222
0.25788 [14]: 22220
0.25777 [9]: 22022
0.11108 [0]: 02222
0.10637 [12]: 22202

SA-CASSCF TRANSITION ENERGIES

LOWEST ROOT (ROOT 0 ,MULT 3) = -2094.636908215 Eh -56997.968 eV

STATE	ROOT	MULT	DE/a.u.	DE/eV	DE/cm**-1
1:	1	3	0.038962	1.060	8551.2
2:	2	3	0.039329	1.070	8631.8
3:	3	3	0.041003	1.116	8999.0
4:	4	3	0.066871	1.820	14676.6
5:	5	3	0.068220	1.856	14972.5
6:	6	3	0.068430	1.862	15018.7
7:	0	1	0.083375	2.269	18298.7
8:	1	1	0.083596	2.275	18347.3
9:	2	1	0.119218	3.244	26165.3
10:	3	1	0.119715	3.258	26274.4
11:	4	1	0.121176	3.297	26595.1
12:	5	1	0.134948	3.672	29617.8
13:	7	3	0.136409	3.712	29938.3
14:	8	3	0.137228	3.734	30118.2
15:	9	3	0.138397	3.766	30374.5
16:	6	1	0.148501	4.041	32592.1
17:	7	1	0.151017	4.109	33144.5
18:	8	1	0.151311	4.117	33208.9
19:	9	1	0.177626	4.833	38984.4
20:	10	1	0.178178	4.848	39105.5
21:	11	1	0.180362	4.908	39585.0
22:	12	1	0.181112	4.928	39749.4
23:	13	1	0.181398	4.936	39812.2
24:	14	1	0.329610	8.969	72341.1

NEVPT2 TRANSITION ENERGIES

LOWEST ROOT (ROOT 0, MULT 3) = -2097.708634995 Eh -57081.554 eV

STATE	ROOT	MULT	DE/a.u.	DE/eV	DE/cm**-1
1:	1	3	0.055778	1.518	12241.8
2:	2	3	0.056219	1.530	12338.6
3:	3	3	0.058123	1.582	12756.5
4:	0	1	0.072377	1.969	15884.9
5:	1	1	0.072694	1.978	15954.5
6:	4	3	0.091651	2.494	20115.1
7:	5	3	0.093800	2.552	20586.8
8:	6	3	0.093848	2.554	20597.2
9:	5	1	0.124512	3.388	27327.2
10:	2	1	0.126335	3.438	27727.3
11:	3	1	0.126929	3.454	27857.7
12:	4	1	0.128788	3.504	28265.7
13:	7	3	0.143179	3.896	31424.1
14:	8	3	0.143771	3.912	31554.1
15:	9	3	0.145304	3.954	31890.4
16:	6	1	0.149612	4.071	32836.1
17:	7	1	0.152461	4.149	33461.3
18:	8	1	0.152828	4.159	33541.9
19:	9	1	0.194308	5.287	42645.6
20:	10	1	0.194860	5.302	42766.9
21:	11	1	0.196225	5.340	43066.3
22:	12	1	0.197069	5.363	43251.7
23:	13	1	0.197259	5.368	43293.3
24:	14	1	0.312824	8.512	68657.0

Racah Parameters :

B = 0.005434025 a.u. = 0.148 eV = 1192.6 cm**-1
C = 0.017148476 a.u. = 0.467 eV = 3763.7 cm**-1
C/B = 3.156

The ligand field one electron eigenfunctions:

```
----------------------------------------
Orbital   Energy (eV)  Energy(cm-1)     dz2       dxz       dyz       dx2-y2     dxy
   1        0.000        0.0        0.155518  -0.752822  0.632382  0.031633  0.090361
   2        0.009       70.4        0.042524  0.526895  0.522881  -0.030867  0.667994
   3        0.025      198.2        0.034174  0.384924  0.544774  0.171023  -0.724319
   4        1.292    10424.6        0.986305  0.082481  -0.141776  -0.003184  -0.017016
   5        1.375    11091.1       -0.006412  -0.025898  -0.099043  0.984270  0.143844

D   =   -0.931115  cm-1
E/D =    0.289747

g-factors:
   2.180631     2.185825     2.190407 iso =     2.185621
```

Listings S3. Selected output of the stated-averaged CAS(12,12)/SC-NEVPT2 calculation for **2'**.

```
----------------------------
LOEWDIN REDUCED ACTIVE MOs
----------------------------

                  81        82        83
              -0.52334  -0.55344  -0.37111
               1.99796   1.99766   1.59341
              --------  --------  --------
 0 Ni dz2          0.0      18.0      88.5
 0 Ni dx2y2       15.3       0.0       0.0
 1 N  pz           0.0      19.6       2.0
 4 C  pz           0.1       0.1       0.0
10 N  px          19.5       5.8       0.6
13 N  pz           0.0      20.6       2.0
22 N  py          20.2       5.7       0.6
25 O  px           8.7       2.5       0.3
25 O  py           0.4       0.0       0.0
28 N  py           0.1       0.0       0.0
37 O  px           0.1       0.1       0.0
37 O  py           7.9       2.2       0.3
40 N  px           0.1       0.0       0.0

                  84        85        86        87        88        89
              -0.38071  -0.42335  -0.42342  -0.42303   2.06768   2.07738
               1.59295   1.59148   1.59145   1.59120   0.00941   0.00937
              --------  --------  --------  --------  --------  --------
 0 Ni dxz          0.5      44.2      52.3       1.2      39.4      53.9
 0 Ni dyz          0.6      53.1      42.4       1.8      52.1      38.6
 0 Ni dx2y2       88.7       1.1       0.0       1.1       0.1       1.4
 0 Ni dxy          1.0       0.1       3.0      94.4       1.2       0.5

                  90        91        92
               2.10101   2.51464   2.50370
               0.00924   0.00794   0.00792
              --------  --------  --------
 0 Ni s            0.0       0.0       0.0
 0 Ni dz2          0.0      86.5       1.7
 0 Ni dx2y2        1.0       1.6      86.3
 0 Ni dxy         92.0       0.0       1.2
 3 H  s            0.0       0.0       0.0
11 H  s            0.1       0.0       0.0
12 H  s            0.2       0.0       0.0
15 H  s            0.0       0.0       0.0
23 H  s            0.2       0.0       0.0
24 H  s            0.1       0.0       0.0
31 H  s            0.0       0.0       0.0
32 H  s            0.0       0.0       0.0
44 H  s            0.0       0.0       0.0

---------------------------------------------
CAS-SCF STATES FOR BLOCK  1 MULT= 3 NROOTS=10
---------------------------------------------

ROOT   0:  E=   -2399.8766145825 Eh
      0.97291 [  1974]: 221122200000
ROOT   1:  E=   -2399.8349584496 Eh  1.134 eV   9142.5 cm**-1
      0.95776 [  1470]: 221222100000
      0.01608 [  1476]: 221221200000
ROOT   2:  E=   -2399.8295039750 Eh  1.282 eV  10339.6 cm**-1
      0.55806 [   315]: 222112200000
      0.23254 [  1476]: 221221200000
      0.12582 [   244]: 222121200000
      0.05679 [  1547]: 221212200000
      0.00254 [  1470]: 221222100000
ROOT   3:  E=   -2399.8292534450 Eh  1.289 eV  10394.6 cm**-1
      0.53806 [   244]: 222121200000
      0.24607 [  1547]: 221212200000
```

```
      0.12767 [   315]: 222112200000
      0.05184 [  1476]: 221221200000
      0.00743 [   238]: 222122100000
      0.00416 [  1470]: 221222100000
ROOT   4:  E=   -2399.8027968217 Eh  2.009 eV  16201.1 cm**-1
      0.31550 [    21]: 222212100000
      0.24893 [  1547]: 221212200000
      0.16049 [   244]: 222121200000
      0.14765 [    27]: 222211200000
      0.08961 [   238]: 222122100000
      0.00588 [     0]: 222221100000
      0.00502 [  1476]: 221221200000
      0.00386 [   315]: 222112200000
ROOT   5:  E=   -2399.8007618790 Eh  2.064 eV  16647.7 cm**-1
      0.46816 [     0]: 222221100000
      0.30213 [  1476]: 221221200000
      0.14606 [   315]: 222112200000
      0.02451 [    21]: 222212100000
      0.01292 [  1547]: 221212200000
      0.00826 [    27]: 222211200000
      0.00558 [   244]: 222121200000
      0.00550 [   238]: 222122100000
      0.00507 [  1470]: 221222100000
ROOT   6:  E=   -2399.7979229133 Eh  2.141 eV  17270.8 cm**-1
      0.35658 [    27]: 222211200000
      0.35128 [   238]: 222122100000
      0.16617 [    21]: 222212100000
      0.05116 [  1547]: 221212200000
      0.02885 [     0]: 222221100000
      0.01041 [  1476]: 221221200000
      0.01021 [   244]: 222121200000
      0.00309 [   315]: 222112200000
ROOT   7:  E=   -2399.7389723603 Eh  3.745 eV  30209.0 cm**-1
      0.32724 [    21]: 222212100000
      0.23866 [  1547]: 221212200000
      0.16526 [   238]: 222122100000
      0.07198 [   244]: 222121200000
      0.06153 [     0]: 222221100000
      0.05017 [    27]: 222211200000
      0.04481 [  1476]: 221221200000
      0.01446 [   315]: 222112200000
ROOT   8:  E=   -2399.7384622234 Eh  3.759 eV  30320.9 cm**-1
      0.41168 [     0]: 222221100000
      0.31084 [  1476]: 221221200000
      0.12093 [   315]: 222112200000
      0.06168 [    21]: 222212100000
      0.04378 [  1547]: 221212200000
      0.01479 [   244]: 222121200000
      0.00585 [   238]: 222122100000
      0.00349 [    27]: 222211200000
      0.00309 [  1470]: 221222100000
ROOT   9:  E=   -2399.7344268161 Eh  3.869 eV  31206.6 cm**-1
      0.41231 [    27]: 222211200000
      0.35090 [   238]: 222122100000
      0.08209 [    21]: 222212100000
      0.07463 [  1547]: 221212200000
      0.04952 [   244]: 222121200000

---------------------------------------------
CAS-SCF STATES FOR BLOCK  2 MULT= 1 NROOTS=15
---------------------------------------------

ROOT   0:  E=   -2399.7973844463 Eh
      0.58910 [  3858]: 220222200000
      0.35292 [   770]: 222022200000
      0.02505 [     0]: 222222000000
ROOT   1:  E=   -2399.7964126430 Eh  0.026 eV    213.3 cm**-1
      0.94514 [  2058]: 221122200000
      0.02866 [    34]: 222211200000
ROOT   2:  E=   -2399.7567371798 Eh  1.106 eV   8921.0 cm**-1
      0.88587 [  1554]: 221222100000
      0.03975 [   105]: 222202200000
      0.03569 [     7]: 222220200000
      0.00896 [   343]: 222112200000
      0.00546 [  1560]: 221221200000
ROOT   3:  E=   -2399.7522329352 Eh  1.229 eV   9909.6 cm**-1
      0.29672 [   272]: 222121200000
      0.21890 [   343]: 222112200000
      0.18716 [  1631]: 221212200000
      0.13198 [  1560]: 221221200000
      0.04744 [    28]: 222212100000
      0.03403 [     1]: 222221100000
      0.03130 [   770]: 222022200000
      0.01350 [  3858]: 220222200000
      0.00732 [   105]: 222202200000
      0.00484 [     7]: 222220200000
```

ROOT 4: E= -2399.7518765801 Eh 1.238 eV 9987.8 cm**-1
0.29075 [343]: 222112200000
0.23739 [272]: 222121200000
0.17410 [1560]: 221221200000
0.15672 [1631]: 221212200000
0.04611 [1]: 222221100000
0.04162 [28]: 222212100000
0.01539 [770]: 222022200000
0.00621 [3858]: 220222200000
0.00300 [105]: 222202200000
ROOT 5: E= -2399.7465873971 Eh 1.382 eV 11148.7 cm**-1
0.47008 [770]: 222022200000
0.27428 [3858]: 220222200000
0.05413 [7]: 222220200000
0.04965 [105]: 222202200000
0.04609 [343]: 222112200000
0.04368 [0]: 222222000000
0.02340 [1560]: 221221200000
0.00587 [1]: 222221100000
0.00272 [272]: 222121200000
ROOT 6: E= -2399.7277886330 Eh 1.894 eV 15274.5 cm**-1
0.35820 [1560]: 221221200000
0.26216 [1631]: 221212200000
0.19069 [343]: 222112200000
0.15538 [272]: 222121200000
0.00453 [1554]: 221222100000
ROOT 7: E= -2399.7274628139 Eh 1.903 eV 15346.0 cm**-1
0.34540 [1631]: 221212200000
0.26243 [1560]: 221221200000
0.20618 [272]: 222121200000
0.14509 [343]: 222112200000
0.01242 [1554]: 221222100000
ROOT 8: E= -2399.7235818363 Eh 2.008 eV 16197.8 cm**-1
0.97130 [266]: 222122100000
0.00403 [272]: 222121200000
ROOT 9: E= -2399.6925084884 Eh 2.854 eV 23017.6 cm**-1
0.60114 [0]: 222222000000
0.15534 [105]: 222202200000
0.13377 [7]: 222220200000
0.05457 [34]: 222211200000
0.01502 [770]: 222022200000
0.01231 [3858]: 220222200000
ROOT 10: E= -2399.6919286671 Eh 2.870 eV 23144.9 cm**-1
0.73388 [34]: 222211200000
0.15369 [28]: 222212100000
0.03538 [0]: 222222000000
0.02504 [2058]: 221122200000
0.00933 [105]: 222202200000
0.00764 [272]: 222121200000
0.00751 [7]: 222220200000
ROOT 11: E= -2399.6902701124 Eh 2.915 eV 23508.9 cm**-1
0.51390 [1]: 222221100000
0.18429 [7]: 222220200000
0.18023 [105]: 222202200000
0.03593 [343]: 222112200000
0.03353 [1554]: 221222100000
0.01532 [1560]: 221221200000
0.01238 [28]: 222212100000
ROOT 12: E= -2399.6891303275 Eh 2.946 eV 23759.0 cm**-1
0.70903 [28]: 222212100000
0.15283 [34]: 222211200000
0.06467 [272]: 222121200000
0.01879 [1631]: 221212200000
0.01839 [105]: 222202200000
0.00839 [7]: 222220200000
ROOT 13: E= -2399.6886767912 Eh 2.958 eV 23858.6 cm**-1
0.37161 [1]: 222221100000
0.26990 [7]: 222220200000
0.24175 [105]: 222202200000
0.03819 [343]: 222112200000
0.03778 [1554]: 221222100000
0.00860 [28]: 222212100000
0.00500 [34]: 222211200000
0.00296 [1560]: 221221200000
ROOT 14: E= -2399.5613455530 Eh 6.423 eV 51804.5 cm**-1
0.26579 [7]: 222220200000
0.26449 [0]: 222222000000
0.26201 [105]: 222202200000
0.08047 [770]: 222022200000
0.06651 [3858]: 220222200000
0.01733 [24572]: 122122200000
0.01495 [9069]: 211222200000

SA-CASSCF TRANSITION ENERGIES

```
LOWEST ROOT (ROOT 0 ,MULT 3) =  -2399.876614583 Eh -65303.963 eV

STATE   ROOT MULT  DE/a.u.     DE/eV    DE/cm**-1
   1:    1    3   0.041656     1.134   9142.5
   2:    2    3   0.047111     1.282  10339.6
   3:    3    3   0.047361     1.289  10394.6
   4:    4    3   0.073818     2.009  16201.1
   5:    5    3   0.075853     2.064  16647.7
   6:    6    3   0.078692     2.141  17270.8
   7:    0    1   0.079230     2.156  17389.0
   8:    1    1   0.080202     2.182  17602.3
   9:    2    1   0.119877     3.262  26310.0
  10:    3    1   0.124382     3.385  27298.6
  11:    4    1   0.124738     3.394  27376.8
  12:    5    1   0.130027     3.538  28537.7
  13:    7    3   0.137642     3.745  30209.0
  14:    8    3   0.138152     3.759  30320.9
  15:    9    3   0.142188     3.869  31206.6
  16:    6    1   0.148826     4.050  32663.5
  17:    7    1   0.149152     4.059  32735.0
  18:    8    1   0.153033     4.164  33586.8
  19:    9    1   0.184106     5.010  40406.6
  20:   10    1   0.184686     5.026  40533.9
  21:   11    1   0.186344     5.071  40897.9
  22:   12    1   0.187484     5.102  41148.0
  23:   13    1   0.187938     5.114  41247.6
  24:   14    1   0.315269     8.579  69193.6

-----------------------------
 NEVPT2 TRANSITION ENERGIES
------------------------------

LOWEST ROOT (ROOT 0, MULT 3) =  -2403.868606440 Eh -65412.590 eV

STATE ROOT MULT  DE/a.u.     DE/eV    DE/cm**-1
   1:   1    3   0.044016     1.198   9660.3
   2:   2    3   0.049950     1.359  10962.8
   3:   3    3   0.050243     1.367  11027.1
   4:   0    1   0.066616     1.813  14620.4
   5:   1    1   0.069291     1.886  15207.6
   6:   4    3   0.076699     2.087  16833.6
   7:   5    3   0.078704     2.142  17273.6
   8:   6    3   0.081908     2.229  17976.8
   9:   2    1   0.113542     3.090  24919.5
  10:   5    1   0.115215     3.135  25286.7
  11:   3    1   0.117145     3.188  25710.4
  12:   4    1   0.117661     3.202  25823.7
  13:   7    3   0.130593     3.554  28661.8
  14:   8    3   0.131102     3.567  28773.5
  15:   9    3   0.134920     3.671  29611.4
  16:   6    1   0.138378     3.765  30370.4
  17:   7    1   0.138749     3.776  30451.9
  18:   8    1   0.142873     3.888  31357.1
  19:   9    1   0.179313     4.879  39354.6
  20:  10    1   0.180063     4.900  39519.4
  21:  11    1   0.181450     4.938  39823.7
  22:  12    1   0.182137     4.956  39974.4
  23:  13    1   0.182604     4.969  40077.0
  24:  14    1   0.290830     7.914  63829.7

D   =   -4.560018  cm-1
E/D =    0.050219

g-factors:
    2.197016     2.199129     2.232439 iso =     2.209528
```

Listings S4. Selected output of the stated-averaged CAS(12,12)/SC-NEVPT2 calculation for **3'**.

```
----------------------------
LOEWDIN REDUCED ACTIVE MOs
----------------------------

                   58        59
                 -0.50431  -0.50826
                  1.99734   1.99718
                 --------  --------
 0 Ni dz2           18.0       0.0
 0 Ni dx2y2          0.0      18.5
 1 N  pz            18.6       0.0
 4 N  py             5.3      13.5
 7 N  px             4.3      14.2
13 C  py             0.0       0.2
16 C  px             0.1       0.1
```

```
19 N  py           4.1      13.8
22 C  py           0.1       0.0
25 N  pz          17.8       0.0
28 N  px           5.5      13.6
31 C  pz           0.1       0.0

                    60        61        62        63        64        65
                 -0.37208  -0.36812  -0.42184  -0.42184  -0.42124   2.03341
                  1.59318   1.59318   1.59143   1.59137   1.59132   0.00964
                 --------  --------  --------  --------  --------  --------
 0 Ni dz2           86.4       0.5       0.3       2.4       0.0       2.3
 0 Ni dxz            0.6       0.0      34.9      54.9       8.0      55.5
 0 Ni dyz            1.7       1.1      51.8      40.2       3.7      35.8
 0 Ni dx2y2          0.7      85.5       1.8       0.1       1.2       0.1
 0 Ni dxy            0.1       1.8       9.9       1.0      85.6       0.2

                    66        67        68        69
                  2.03379   2.05436   2.47525   2.49140
                  0.00963   0.00955   0.00811   0.00806
                 --------  --------  --------  --------
 0 Ni dz2            0.2       0.2      87.2       0.0
 0 Ni dxz           12.0      25.9       0.6       0.1
 0 Ni dyz           12.8      42.6       1.9       0.8
 0 Ni dx2y2          0.6       2.3       0.0      86.4
 0 Ni dxy           68.1      23.4       0.0       1.9
 2 H  s              0.0       0.1       0.1       0.0
 5 H  s              0.0       0.1       0.0       0.0
 8 H  s              0.0       0.1       0.0       0.0
20 H  s              0.0       0.1       0.0       0.0
26 H  s              0.0       0.1       0.1       0.0
29 H  s              0.0       0.1       0.0       0.0

---------------------------------------------
CAS-SCF STATES FOR BLOCK  1 MULT= 3 NROOTS=10
---------------------------------------------

ROOT   0:  E=   -2094.7734986109 Eh
      0.97206 [  1974]: 221122200000
      0.00255 [  2001]: 221121111000
      0.00254 [  2187]: 221111210100
ROOT   1:  E=   -2094.7266574010 Eh  1.275 eV  10280.5 cm**-1
      0.69107 [   315]: 222112200000
      0.21444 [  1476]: 221221200000
      0.06298 [   238]: 222122100000
      0.00516 [  1547]: 221212200000
ROOT   2:  E=   -2094.7262190710 Eh  1.287 eV  10376.7 cm**-1
      0.73824 [   244]: 222121200000
      0.22776 [  1547]: 221212200000
      0.00543 [  1476]: 221221200000
ROOT   3:  E=   -2094.7242205441 Eh  1.341 eV  10815.3 cm**-1
      0.89319 [  1470]: 221222100000
      0.06694 [  1547]: 221212200000
      0.00839 [   244]: 222121200000
      0.00668 [   238]: 222122100000
ROOT   4:  E=   -2094.6952924676 Eh  2.128 eV  17164.3 cm**-1
      0.45330 [    27]: 222211200000
      0.32121 [   238]: 222122100000
      0.10385 [   315]: 222112200000
      0.08298 [  1476]: 221221200000
      0.01245 [     0]: 222221100000
ROOT   5:  E=   -2094.6934748852 Eh  2.178 eV  17563.2 cm**-1
      0.49977 [    21]: 222212100000
      0.33302 [  1547]: 221212200000
      0.10368 [   244]: 222121200000
      0.03770 [  1470]: 221222100000
      0.00259 [   315]: 222112200000
ROOT   6:  E=   -2094.6932895829 Eh  2.183 eV  17603.8 cm**-1
      0.47703 [     0]: 222221100000
      0.30259 [  1476]: 221221200000
      0.15022 [   238]: 222122100000
      0.03205 [   315]: 222112200000
      0.01191 [    27]: 222211200000
ROOT   7:  E=   -2094.6323003403 Eh  3.842 eV  30989.4 cm**-1
      0.43348 [   238]: 222122100000
      0.40967 [    27]: 222211200000
      0.09808 [     0]: 222221100000
      0.02129 [   315]: 222112200000
      0.00962 [  1476]: 221221200000
      0.00317 [  1470]: 221222100000
ROOT   8:  E=   -2094.6315997326 Eh  3.861 eV  31143.2 cm**-1
      0.47805 [    21]: 222212100000
      0.34012 [  1547]: 221212200000
      0.11843 [   244]: 222121200000
      0.03542 [  1470]: 221222100000
      0.00255 [   315]: 222112200000
ROOT   9:  E=   -2094.6302298068 Eh  3.899 eV  31443.9 cm**-1
```

```
     0.39033 [     0]: 222221100000
     0.35889 [  1476]: 221221200000
     0.11974 [   315]: 222112200000
     0.10302 [    27]: 222211200000
     0.00267 [   244]: 222121200000

---------------------------------------------
CAS-SCF STATES FOR BLOCK  2 MULT= 1 NROOTS=15
---------------------------------------------

ROOT   0:  E=   -2094.6942232065 Eh
     0.51701 [   770]: 220222200000
     0.40296 [  3858]: 220222200000
     0.02743 [  2058]: 221122200000
     0.01032 [     0]: 222222000000
     0.00629 [    28]: 222212100000
     0.00556 [     7]: 222220200000
     0.00360 [   105]: 222202200000
ROOT   1:  E=   -2094.6939933140 Eh  0.006 eV     50.5 cm**-1
     0.92015 [  2058]: 221122200000
     0.02324 [    34]: 222211200000
     0.01378 [   770]: 222022200000
     0.01376 [  3858]: 220222200000
ROOT   2:  E=   -2094.6490243037 Eh  1.230 eV   9920.0 cm**-1
     0.66141 [   343]: 222112200000
     0.17867 [  1560]: 221221200000
     0.06718 [     1]: 222221100000
     0.05522 [   266]: 222122100000
     0.00756 [    34]: 222211200000
     0.00486 [  1631]: 221212200000
ROOT   3:  E=   -2094.6485064991 Eh  1.244 eV  10033.7 cm**-1
     0.69142 [   272]: 222121200000
     0.16082 [  1631]: 221212200000
     0.05128 [    28]: 222212100000
     0.02143 [   105]: 222202200000
     0.01967 [  1554]: 221222100000
     0.01049 [  3858]: 220222200000
     0.00842 [   770]: 222022200000
     0.00538 [     0]: 222222000000
     0.00518 [  1560]: 221221200000
ROOT   4:  E=   -2094.6466323668 Eh  1.295 eV  10445.0 cm**-1
     0.77902 [  1554]: 221222100000
     0.08563 [  1631]: 221212200000
     0.05473 [     7]: 222220200000
     0.02129 [   105]: 222202200000
     0.01255 [  3858]: 220222200000
     0.01004 [   770]: 222022200000
     0.00612 [    28]: 222212100000
     0.00581 [   266]: 222122100000
ROOT   5:  E=   -2094.6453528872 Eh  1.330 eV  10725.8 cm**-1
     0.44054 [  3858]: 220222200000
     0.33850 [   770]: 222022200000
     0.06227 [     0]: 222222000000
     0.05163 [   105]: 222202200000
     0.03022 [     7]: 222220200000
     0.01651 [   272]: 222121200000
     0.01630 [  1554]: 221222100000
     0.01230 [  1631]: 221212200000
ROOT   6:  E=   -2094.6242607465 Eh  1.904 eV  15355.0 cm**-1
     0.87762 [   266]: 222122100000
     0.08755 [   343]: 222112200000
     0.00649 [  1554]: 221222100000
ROOT   7:  E=   -2094.6213176800 Eh  1.984 eV  16000.9 cm**-1
     0.68219 [  1631]: 221212200000
     0.20509 [   272]: 222121200000
     0.07985 [  1554]: 221222100000
     0.00579 [   343]: 222112200000
ROOT   8:  E=   -2094.6209479051 Eh  1.994 eV  16082.1 cm**-1
     0.76477 [  1560]: 221221200000
     0.17101 [   343]: 222112200000
     0.03019 [   266]: 222122100000
     0.00492 [   272]: 222121200000
ROOT   9:  E=   -2094.5850906957 Eh  2.970 eV  23951.8 cm**-1
     0.43678 [     0]: 222222000000
     0.28555 [    28]: 222212100000
     0.14896 [   105]: 222202200000
     0.07963 [     7]: 222220200000
     0.01344 [  3858]: 220222200000
     0.01042 [   770]: 222022200000
ROOT  10:  E=   -2094.5845154212 Eh  2.985 eV  24078.1 cm**-1
     0.87869 [    34]: 222211200000
     0.07359 [     1]: 222221100000
     0.02430 [  2058]: 221122200000
ROOT  11:  E=   -2094.5829579595 Eh  3.028 eV  24419.9 cm**-1
     0.58407 [    28]: 222212100000
     0.16624 [   105]: 222202200000
```

```
      0.15083 [     0]: 222222000000
      0.05578 [   272]: 222121200000
      0.01720 [  1631]: 221212200000
      0.00275 [  1554]: 221222100000
ROOT  12:  E=   -2094.5821710035 Eh  3.049 eV  24592.6 cm**-1
      0.83438 [     1]: 222221100000
      0.06844 [    34]: 222211200000
      0.04720 [   343]: 222112200000
      0.02284 [  1560]: 221221200000
      0.00429 [   266]: 222122100000
ROOT  13:  E=   -2094.5818896742 Eh  3.057 eV  24654.4 cm**-1
      0.52814 [     7]: 222220200000
      0.29168 [   105]: 222202200000
      0.06914 [  1554]: 221222100000
      0.04381 [    28]: 222212100000
      0.03525 [     0]: 222222000000
      0.00837 [  1631]: 221212200000
ROOT  14:  E=   -2094.4583093813 Eh  6.420 eV  51777.1 cm**-1
      0.26639 [     7]: 222220200000
      0.26500 [     0]: 222222000000
      0.26096 [   105]: 222202200000
      0.06657 [  3858]: 220222200000
      0.06277 [   770]: 222022200000
      0.02427 [  7269]: 212122200000
      0.02254 [ 26372]: 121222200000
```

SA-CASSCF TRANSITION ENERGIES

LOWEST ROOT (ROOT 0 ,MULT 3) = -2094.773498611 Eh -57001.685 eV

STATE	ROOT	MULT	DE/a.u.	DE/eV	DE/cm**-1
1:	1	3	0.046841	1.275	10280.5
2:	2	3	0.047280	1.287	10376.7
3:	3	3	0.049278	1.341	10815.3
4:	4	3	0.078206	2.128	17164.3
5:	0	1	0.079275	2.157	17398.9
6:	1	1	0.079505	2.163	17449.4
7:	5	3	0.080024	2.178	17563.2
8:	6	3	0.080209	2.183	17603.8
9:	2	1	0.124474	3.387	27319.0
10:	3	1	0.124992	3.401	27432.6
11:	4	1	0.126866	3.452	27843.9
12:	5	1	0.128146	3.487	28124.7
13:	7	3	0.141198	3.842	30989.4
14:	8	3	0.141899	3.861	31143.2
15:	9	3	0.143269	3.899	31443.9
16:	6	1	0.149238	4.061	32753.9
17:	7	1	0.152181	4.141	33399.9
18:	8	1	0.152551	4.151	33481.0
19:	9	1	0.188408	5.127	41350.8
20:	10	1	0.188983	5.142	41477.0
21:	11	1	0.190541	5.185	41818.8
22:	12	1	0.191328	5.206	41991.6
23:	13	1	0.191609	5.214	42053.3
24:	14	1	0.315189	8.577	69176.0

NEVPT2 TRANSITION ENERGIES

LOWEST ROOT (ROOT 0, MULT 3) = -2097.659112256 Eh -57080.206 eV

STATE	ROOT	MULT	DE/a.u.	DE/eV	DE/cm**-1
1:	1	3	0.049910	1.358	10954.0
2:	2	3	0.050236	1.367	11025.6
3:	3	3	0.052294	1.423	11477.3
4:	0	1	0.066487	1.809	14592.1
5:	1	1	0.067118	1.826	14730.8
6:	4	3	0.081260	2.211	17834.6
7:	5	3	0.083351	2.268	18293.4
8:	6	3	0.083522	2.273	18330.9
9:	5	1	0.111939	3.046	24567.7
10:	2	1	0.117441	3.196	25775.4
11:	3	1	0.117732	3.204	25839.2
12:	4	1	0.119845	3.261	26302.9
13:	7	3	0.134789	3.668	29582.8
14:	8	3	0.135208	3.679	29674.7
15:	9	3	0.136755	3.721	30014.3
16:	6	1	0.138471	3.768	30391.0
17:	7	1	0.141638	3.854	31086.0
18:	8	1	0.142143	3.868	31196.8
19:	9	1	0.184125	5.010	40410.9
20:	10	1	0.184774	5.028	40553.3
21:	11	1	0.185974	5.061	40816.6

```
22:  13   1   0.186811    5.083  41000.2
23:  12   1   0.186862    5.085  41011.4
24:  14   1   0.290209    7.897  63693.5

D   =   1.152441  cm-1
E/D =   0.295284

g-factors:
   2.189491    2.196670    2.201210 iso =    2.195790
```

Listings S5. Cartezian coordinates of $[Ni(en)_2(dmf)_2]^{2+}$ and $[Ni(en)_3]^{2+}$ with the positions of H atoms optimized at the ωB97X-D4/ma-def2-TZVP level (all other atomic coordinates constrained to the crystallographic data). The Ni–N(O) bonds are aligned with the molecular frame.

$[Ni(en)_2(dmf)_2]^{2+}$
Ni 0.00000 0.00000 0.00000
N 0.06242 0.03871 -2.10649
H -0.66032 -0.56533 -2.48652
H -0.07200 0.94869 -2.53755
C 1.38354 -0.49250 -2.49483
H 1.59784 -0.33054 -3.55413
H 1.37586 -1.56604 -2.30909
C 2.43347 0.18235 -1.64085
H 3.42889 -0.19921 -1.88070
H 2.44367 1.25752 -1.84448
N 2.09978 -0.00413 -0.20664
H 2.65290 0.63584 0.35427
H 2.38658 -0.93703 0.07515
N 0.00000 0.00000 2.09197
H -0.93474 -0.29498 2.36139
H 0.64978 -0.60941 2.57708
C 0.22119 1.39860 2.52937
H 1.29330 1.60538 2.45912
H -0.06643 1.55164 3.57282
C -0.54679 2.32934 1.63493
H -1.61730 2.12726 1.71574
H -0.38109 3.36804 1.93115
N -0.14690 2.07922 0.23314
H 0.74034 2.54139 0.05274
H -0.81126 2.52859 -0.38932
O -2.06627 -0.15586 0.21040
C -2.91183 0.31339 -0.55978
H -2.63673 0.68644 -1.55566
N -4.19446 0.40295 -0.28791
C -4.72525 -0.00033 0.98674
H -3.92067 -0.35169 1.62632
H -5.45177 -0.80535 0.85240
H -5.23124 0.84419 1.46091
C -5.11863 0.90567 -1.28703
H -4.57793 1.19281 -2.18844
H -5.65329 1.77637 -0.90124
H -5.84757 0.13419 -1.54464
O 0.14819 -2.07872 -0.21376
C 0.09409 -2.93740 0.66922
H -0.36000 -2.73210 1.64776
N 0.52480 -4.17404 0.52342
C 1.20594 -4.59167 -0.67653
H 0.98047 -3.90739 -1.49026
H 0.87068 -5.59229 -0.95381
H 2.28682 -4.62454 -0.51105
C 0.45657 -5.13996 1.60478
H -0.03272 -4.69843 2.47314
H 1.46230 -5.45989 1.88685
H -0.11436 -6.01446 1.28709

$[Ni(en)_3]^{2+}$
Ni 0.00000 0.00000 0.00000
N 0.00000 -0.00000 2.14900
H -0.62891 -0.67225 2.57860
H 0.91642 -0.22716 2.52696
N -0.04579 2.09440 0.29384
H 0.59020 2.60566 -0.31128
H -0.96005 2.49528 0.10058
N 2.12040 0.00903 -0.13229
H 2.40190 0.31539 -1.06041
H 2.59859 0.64236 0.50169
C -0.37043 1.36261 2.59768
H -0.10240 1.53934 3.64195
H -1.45761 1.45892 2.52531
C 0.30285 2.37082 1.70219
H 0.03064 3.38646 2.00009
H 1.38913 2.29369 1.80488
C 2.61992 -1.35799 0.08363
H 2.63158 -1.55125 1.16047
H 3.64313 -1.48925 -0.27617
N 0.30708 -2.09604 -0.16122
H 0.17427 -2.52976 0.74915
H -0.33416 -2.58963 -0.77515
C 1.68772 -2.34548 -0.60446
H 1.73044 -2.19936 -1.68793
H 2.00969 -3.37070 -0.40712
N -0.29631 0.25475 -2.11318

H 0.21531 1.02795 -2.52838
H 0.00292 -0.56317 -2.63830
N -2.10040 -0.24923 -0.03435
H -2.42875 -1.02490 0.53360
H -2.59515 0.56121 0.32945
C -1.74615 0.45712 -2.34177
H -2.02571 0.29025 -3.38463
H -1.98336 1.49938 -2.10962
C -2.51665 -0.46751 -1.43451
H -3.59173 -0.32505 -1.56965
H -2.30241 -1.50824 -1.69431

Listings S6. Cartezian coordinates of $[Ni(en)_2(dmf)_2]^{2+}$ and $[Ni(en)_3]^{2+}$ with the positions of all atoms optimized at the ωB97X-D4/ma-def2-TZVP level. The Ni–N(O) bonds are aligned with the molecular frame.

$[Ni(en)_2(dmf)_2]^{2+}$
Ni 0.00000 0.00000 0.00000
N 0.05654 0.00191 -2.13112
H -0.60512 -0.66988 -2.50631
H -0.16794 0.89340 -2.56312
C 1.43111 -0.39666 -2.52502
H 1.63243 -0.18019 -3.57665
H 1.51311 -1.47534 -2.38012
C 2.43108 0.31963 -1.63587
H 3.45030 0.03298 -1.90700
H 2.34966 1.40230 -1.77104
N 2.11967 0.00666 -0.22325
H 2.68051 0.58693 0.39173
H 2.38185 -0.95552 -0.02515
N -0.00000 0.00000 2.11379
H -0.92228 -0.32215 2.39266
H 0.66810 -0.63026 2.54434
C 0.20968 1.38390 2.58927
H 1.27865 1.60654 2.52710
H -0.08871 1.51436 3.63247
C -0.57483 2.32981 1.69747
H -1.64106 2.10274 1.76627
H -0.43011 3.36445 2.01730
N -0.16224 2.12163 0.28713
H 0.72544 2.58983 0.12703
H -0.82481 2.58501 -0.32641
O -2.10849 -0.18971 0.16580
C -3.01386 0.21126 -0.59472
H -2.77363 0.68308 -1.55719
N -4.30559 0.12672 -0.35161
C -4.82699 -0.46417 0.87789
H -4.00076 -0.77072 1.51360
H -5.44621 -1.32972 0.63339
H -5.44154 0.27018 1.40229
C -5.29610 0.61613 -1.31018
H -4.79646 1.03642 -2.18272
H -5.91090 1.38819 -0.84399
H -5.93956 -0.20592 -1.62911
O 0.21345 -2.08899 -0.09638
C -0.62768 -2.95513 0.22876
H -1.62471 -2.65685 0.57230
N -0.42008 -4.25514 0.19180
C 0.84922 -4.82865 -0.24673
H 1.52055 -4.03258 -0.55629
H 0.67151 -5.50645 -1.08391
H 1.30017 -5.39158 0.57314
C -1.45629 -5.20162 0.59875
H -2.35084 -4.66289 0.91029
H -1.09686 -5.80852 1.43201
H -1.70656 -5.85906 -0.23607

$[Ni(en)_3]^{2+}$
Ni 0.00000 0.00000 0.00000
N -0.00000 0.00000 2.16419
H -0.60444 -0.69867 2.58658
H 0.92374 -0.18863 2.54455
N -0.07946 2.13861 0.32647
H 0.56030 2.66467 -0.26156
H -0.99627 2.52858 0.12572
N 2.16202 0.00022 -0.10581
H 2.47051 0.28524 -1.03171
H 2.63349 0.63482 0.53177
C -0.43231 1.34733 2.61285
H -0.20785 1.51809 3.66835
H -1.51793 1.41003 2.49896
C 0.24540 2.39621 1.75205
H -0.05511 3.39885 2.06483
H 1.33056 2.33449 1.87079
C 2.63374 -1.38428 0.14525
H 2.58889 -1.57017 1.22208
H 3.67205 -1.52531 -0.16387
N 0.32733 -2.13476 -0.14769
H 0.19905 -2.57496 0.75977

H -0.30085 -2.63466 -0.76979
C 1.72763 -2.35506 -0.58714
H 1.77960 -2.17490 -1.66462
H 2.05348 -3.38359 -0.41553
N -0.25301 0.21374 -2.13809
H 0.29693 0.95841 -2.55638
H 0.02145 -0.62737 -2.63882
N -2.15092 -0.22305 -0.09781
H -2.50755 -0.99150 0.46265
H -2.64187 0.59799 0.24537
C -1.69180 0.47244 -2.39654
H -1.95037 0.32855 -3.44823
H -1.89680 1.51853 -2.15332
C -2.52421 -0.44169 -1.51802
H -3.58926 -0.27147 -1.69138
H -2.32134 -1.48697 -1.76652

Listings S7. Cartezian coordinates of $[Ni(en)_2(dmf)_2]^{2+}$ with the positions of all atoms optimized at the ωB97X-D4/ma-def2-TZVP level, keeping the $[O]_{dmf1}$–Ni–$[O–C]_{dmf2}$ torsion angle constrained to the indicated value (the angle of –16.85° corresponds to the fully optimized structure, Listing S6). The Ni–N(O) bonds are aligned with the molecular frame.

-135
Ni 0.00000 0.00000 0.00000
N 0.01887 0.02704 -2.12037
H -0.64181 -0.65913 -2.46857
H -0.26805 0.91834 -2.51292
C 1.38586 -0.31028 -2.57351
H 1.55577 -0.03690 -3.61757
H 1.50588 -1.39320 -2.49106
C 2.38839 0.39009 -1.67378
H 3.41027 0.16019 -1.98504
H 2.26180 1.47394 -1.74375
N 2.13085 -0.01221 -0.26914
H 2.65730 0.58589 0.35983
H 2.49948 -0.94775 -0.12544
N -0.00000 -0.00000 2.12454
H -0.94009 -0.28110 2.38946
H 0.62961 -0.63469 2.60371
C 0.24523 1.38691 2.58407
H 1.31941 1.58109 2.51445
H -0.04514 1.53136 3.62769
C -0.52171 2.34128 1.68736
H -1.59121 2.13524 1.75807
H -0.35624 3.37469 2.00053
N -0.11770 2.11632 0.27837
H 0.72928 2.64027 0.07997
H -0.83341 2.49164 -0.33552
O -2.09668 0.12739 0.05412
C -2.94025 -0.75896 -0.20245
H -2.61705 -1.77840 -0.44110
N -4.24444 -0.57309 -0.21048
C -4.84816 0.72253 0.08782
H -4.06639 1.45488 0.26887
H -5.48378 0.63600 0.97154
H -5.46161 1.04135 -0.75718
C -5.16530 -1.66536 -0.51629
H -4.60582 -2.57864 -0.71728
H -5.76305 -1.41166 -1.39387
H -5.83364 -1.83486 0.33014
O -0.14237 -2.10774 -0.19513
C 0.47385 -3.03485 0.36863
H 1.18723 -2.82906 1.17889
N 0.35348 -4.31456 0.07986
C -0.54420 -4.79047 -0.96872
H -0.97557 -3.94096 -1.49152
H -1.33847 -5.39627 -0.52701
H 0.01721 -5.40746 -1.67268
C 1.09921 -5.33661 0.81370
H 1.72822 -4.86929 1.57105
H 1.72913 -5.90116 0.12393
H 0.40398 -6.02203 1.30204

-90
Ni 0.00000 0.00000 0.00000
N 0.05096 0.05311 -2.12536
H -0.60504 -0.61972 -2.50679
H -0.21356 0.95371 -2.51272
C 1.42524 -0.29460 -2.55305
H 1.61637 -0.01741 -3.59239
H 1.53198 -1.37831 -2.46994
C 2.41795 0.39046 -1.63135
H 3.44196 0.14413 -1.92276
H 2.31062 1.47659 -1.70173
N 2.12399 -0.01090 -0.23533
H 2.66605 0.55790 0.40705
H 2.43740 -0.96706 -0.09364
N -0.00000 -0.00000 2.12976
H -0.93482 -0.28456 2.40977
H 0.64588 -0.62303 2.60284
C 0.23889 1.39179 2.57981
H 1.30999 1.59542 2.49347

H -0.03768 1.53713 3.62700
C -0.54916 2.33480 1.69018
H -1.61532 2.11527 1.77194
H -0.39383 3.37071 2.00031
N -0.15519 2.11164 0.27863
H 0.68889 2.63804 0.07404
H -0.87625 2.48430 -0.33057
O -2.09879 0.04763 0.08554
C -2.90252 -0.77872 -0.39701
H -2.53820 -1.70197 -0.86375
N -4.21263 -0.64324 -0.39708
C -4.87338 0.52287 0.18278
H -4.12509 1.21633 0.55619
H -5.52318 0.20646 1.00135
H -5.48120 1.01308 -0.58024
C -5.08292 -1.66215 -0.98000
H -4.48372 -2.47944 -1.38078
H -5.67590 -1.22563 -1.78593
H -5.75776 -2.05412 -0.21660
O -0.00664 -2.10579 -0.26937
C 0.02539 -3.05632 0.53966
H -0.00160 -2.87917 1.62264
N 0.08760 -4.33036 0.20984
C 0.13689 -4.77018 -1.18185
H 0.07428 -3.90702 -1.83899
H -0.69743 -5.44601 -1.37975
H 1.07150 -5.30463 -1.36429
C 0.12636 -5.38047 1.22704
H 0.08985 -4.93854 2.22247
H 1.04685 -5.95852 1.12634
H -0.72754 -6.04870 1.10153

-77.41
Ni 0.00000 0.00000 0.00000
N 0.28561 -0.20161 -2.09993
H -0.31741 -0.92732 -2.47181
H 0.06102 0.64215 -2.61841
C 1.70187 -0.56984 -2.32919
H 2.00533 -0.41318 -3.36695
H 1.80561 -1.63374 -2.10602
C 2.58165 0.23782 -1.39227
H 3.63285 -0.02344 -1.53702
H 2.47824 1.30583 -1.60429
N 2.13444 -0.00000 0.00000
H 2.60410 0.64513 0.62701
H 2.42411 -0.93087 0.28704
N -0.23006 0.25751 2.10081
H -1.18931 -0.00108 2.31562
H 0.36347 -0.29777 2.70805
C -0.03912 1.69630 2.39998
H 1.03486 1.90269 2.39725

H -0.42060 1.96389 3.38848
C -0.73497 2.51299 1.32717
H -1.80338 2.28876 1.32779
H -0.61504 3.58108 1.52258
N -0.19906 2.12675 0.00000
H 0.65958 2.63790 -0.18138
H -0.85547 2.41195 -0.71954
O -2.09822 0.00533 -0.13694
C -2.83362 -0.86488 -0.64982
H -2.40943 -1.80935 -1.01305
N -4.13725 -0.75646 -0.80284
C -4.87304 0.43136 -0.37770
H -4.17963 1.16212 0.02908
H -5.60570 0.15556 0.38355
H -5.39840 0.85911 -1.23383
C -4.92421 -1.82747 -1.41067
H -4.27420 -2.65747 -1.68683
H -5.42756 -1.45526 -2.30496
H -5.67618 -2.18195 -0.70306
O 0.07032 -2.11990 0.00541
C -0.16467 -2.95449 0.90406
H -0.53362 -2.63702 1.88807
N -0.01329 -4.25778 0.78532
C 0.47140 -4.87283 -0.44756
H 0.61896 -4.10609 -1.20320
H -0.25899 -5.60309 -0.80132
H 1.41553 -5.38651 -0.25455
C -0.30615 -5.16440 1.89471
H -0.65609 -4.59855 2.75779
H 0.59530 -5.71505 2.17011
H -1.07857 -5.87581 1.59683

-45
Ni 0.00000 0.00000 0.00000
N 0.08018 0.05832 -2.12998
H -0.57170 -0.59557 -2.54958
H -0.15672 0.96967 -2.51092
C 1.46005 -0.30506 -2.53294
H 1.66877 -0.03368 -3.57037
H 1.55264 -1.38926 -2.44330
C 2.44575 0.37352 -1.59948
H 3.47027 0.10857 -1.87243
H 2.35687 1.46072 -1.68149
N 2.12154 -0.01100 -0.20715
H 2.67053 0.54429 0.44076
H 2.39407 -0.97828 -0.05385
N -0.00000 -0.00000 2.12496
H -0.93176 -0.27970 2.41849
H 0.64902 -0.63813 2.57330
C 0.25451 1.38651 2.57718
H 1.32511 1.58549 2.47670

H -0.00661 1.53194 3.62837
C -0.54179 2.33528 1.70017
H -1.60795 2.12209 1.79924
H -0.37613 3.37037 2.00763
N -0.17262 2.11132 0.28138
H 0.67358 2.63025 0.06600
H -0.89763 2.49647 -0.31528
O -2.10826 -0.02950 0.13333
C -2.94080 -0.66366 -0.54757
H -2.61279 -1.40704 -1.28634
N -4.24834 -0.53049 -0.46111
C -4.87259 0.41093 0.46510
H -4.10321 0.92594 1.03352
H -5.53283 -0.13051 1.14550
H -5.46420 1.13691 -0.09624
C -5.15223 -1.31344 -1.30196
H -4.57947 -1.97566 -1.95093
H -5.75615 -0.64480 -1.91824
H -5.81554 -1.91216 -0.67490
O 0.12084 -2.09948 -0.19114
C -0.48005 -2.97411 0.46747
H -1.24201 -2.69443 1.20689
N -0.28612 -4.27198 0.35578
C 0.69013 -4.83321 -0.57469
H 1.15152 -4.03169 -1.14486
H 0.18886 -5.52599 -1.25331
H 1.45545 -5.37764 -0.01770
C -1.02904 -5.22713 1.17604
H -1.72109 -4.69809 1.83098
H -0.33550 -5.80930 1.78573
H -1.59209 -5.90708 0.53399

0°
Ni 0.00000 0.00000 0.00000
N 0.05248 -0.00148 -2.13108
H -0.62145 -0.65728 -2.51269
H -0.15667 0.89640 -2.55769
C 1.42130 -0.41870 -2.52489
H 1.62233 -0.21526 -3.57922
H 1.49354 -1.49666 -2.36865
C 2.42996 0.29783 -1.64565
H 3.44595 -0.00044 -1.91630
H 2.35749 1.37960 -1.79274
N 2.11936 0.00329 -0.22910
H 2.68414 0.58754 0.37838
H 2.37498 -0.95819 -0.01960
N 0.00000 0.00000 2.11009
H -0.91988 -0.33282 2.38412
H 0.67153 -0.63116 2.53372
C 0.20108 1.38149 2.59333
H 1.26960 1.60880 2.53969

H -0.10487 1.50722 3.63494
C -0.57971 2.32917 1.69985
H -1.64648 2.10295 1.76401
H -0.43621 3.36323 2.02217
N -0.16093 2.12389 0.29060
H 0.73276 2.58352 0.13907
H -0.81347 2.59931 -0.32451
O -2.10505 -0.21518 0.15663
C -3.02437 0.28776 -0.52210
H -2.80245 0.90352 -1.40461
N -4.31148 0.15067 -0.27808
C -4.80718 -0.62867 0.85337
H -3.96806 -1.03446 1.41204
H -5.43669 -1.44260 0.48792
H -5.40586 0.01254 1.50337
C -5.32155 0.77269 -1.13369
H -4.83989 1.32697 -1.93895
H -5.93365 1.45789 -0.54437
H -5.96487 0.00361 -1.56516
O 0.23353 -2.09055 -0.03631
C -0.64864 -2.97465 0.02222
H -1.70733 -2.69735 0.07374
N -0.41361 -4.27074 0.03113
C 0.93891 -4.81818 -0.02297
H 1.65916 -4.00598 -0.06381
H 1.04329 -5.44812 -0.90873
H 1.12211 -5.42626 0.86519
C -1.50496 -5.23964 0.10233
H -2.46234 -4.72015 0.13428
H -1.39992 -5.85126 1.00053
H -1.48049 -5.89036 -0.77390

45°
Ni 0.00000 0.00000 0.00000
N 0.11923 0.07166 -2.12192
H -0.56457 -0.52537 -2.57452
H -0.08137 1.00828 -2.46035
C 1.49243 -0.31472 -2.51609
H 1.71707 -0.04708 -3.55138
H 1.57448 -1.40087 -2.42849
C 2.47815 0.35336 -1.57313
H 3.50125 0.06650 -1.82851
H 2.41055 1.44072 -1.67125
N 2.12902 -0.00371 -0.17923
H 2.66812 0.56563 0.46484
H 2.39743 -0.96736 0.00219
N 0.00000 0.00000 2.12043
H -0.88697 -0.34730 2.47157
H 0.70696 -0.61318 2.51260
C 0.20028 1.39313 2.57760
H 1.26381 1.63082 2.48754

H -0.07465 1.52856 3.62648
C -0.61815 2.31477 1.69069
H -1.67830 2.06495 1.77393
H -0.49619 3.35489 2.00176
N -0.22020 2.10673 0.27743
H 0.60965 2.65628 0.07503
H -0.95146 2.46142 -0.33052
O -2.09975 -0.02270 -0.14824
C -2.94843 -0.78394 0.35943
H -2.63959 -1.63087 0.98745
N -4.25242 -0.67028 0.20812
C -4.84887 0.39507 -0.59434
H -4.06254 0.99338 -1.04614
H -5.47434 1.02554 0.04107
H -5.47194 -0.04445 -1.37563
C -5.17888 -1.60090 0.84998
H -4.62500 -2.34315 1.42461
H -5.77868 -2.10840 0.09207
H -5.84533 -1.05582 1.52113
O 0.15913 -2.10627 0.05621
C -0.39745 -2.98359 -0.63503
H -1.16029 -2.71415 -1.37836
N -0.15469 -4.27614 -0.55804
C 0.82239 -4.82603 0.37891
H 1.23223 -4.02555 0.98862
H 1.62490 -5.31786 -0.17476
H 0.33464 -5.56290 1.01986
C -0.84825 -5.23548 -1.41570
H -1.54336 -4.71449 -2.07396
H -1.40171 -5.94847 -0.80170
H -0.12294 -5.78038 -2.02285

90°

Ni 0.00000 0.00000 0.00000
N 0.09972 0.07366 -2.12525
H -0.56231 -0.54916 -2.57570
H -0.15498 1.00386 -2.44417
C 1.48382 -0.23513 -2.54861
H 1.68694 0.09012 -3.57175
H 1.61490 -1.31987 -2.51882
C 2.45511 0.42269 -1.58329
H 3.48486 0.18216 -1.85906
H 2.35079 1.51020 -1.63252
N 2.12896 -0.00651 -0.20441
H 2.64847 0.55662 0.46117
H 2.44165 -0.96270 -0.05986
N 0.00000 0.00000 2.11285
H -0.90673 -0.33284 2.42605
H 0.68275 -0.62960 2.52078
C 0.21254 1.38565 2.58350
H 1.28102 1.60869 2.51349

H -0.07841 1.51836 3.62846
C -0.57860 2.32458 1.69013
H -1.64277 2.08919 1.75609
H -0.44372 3.36116 2.00755
N -0.16467 2.11774 0.28040
H 0.67460 2.65796 0.09247
H -0.88409 2.48813 -0.33230
O -2.09467 0.08284 -0.06769
C -2.93647 -0.81761 0.13442
H -2.61444 -1.83190 0.39945
N -4.24201 -0.65649 0.05963
C -4.84785 0.62830 -0.27980
H -4.06707 1.35712 -0.47854
H -5.46982 0.97050 0.54999
H -5.47561 0.51182 -1.16538
C -5.16149 -1.76143 0.32138
H -4.60061 -2.66287 0.56721
H -5.77463 -1.95056 -0.56183
H -5.81537 -1.50899 1.15838
O 0.00474 -2.12091 0.13123
C 0.03212 -3.08384 -0.66106
H -0.03835 -2.93018 -1.74640
N 0.13800 -4.35014 -0.31116
C 0.24440 -4.76094 1.08644
H 0.19001 -3.88535 1.72747
H 1.19410 -5.27594 1.24509
H -0.57065 -5.44621 1.32747
C 0.16752 -5.41813 -1.30944
H 0.08565 -4.99658 -2.31111
H -0.66458 -6.10449 -1.14202
H 1.10481 -5.97208 -1.23041

135°

Ni 0.00000 0.00000 0.00000
N 0.06304 0.05564 -2.12380
H -0.56897 -0.62436 -2.53262
H -0.25926 0.95858 -2.45859
C 1.44979 -0.18512 -2.58091
H 1.62180 0.18405 -3.59474
H 1.61871 -1.26457 -2.59982
C 2.41813 0.47062 -1.61079
H 3.45000 0.27129 -1.91049
H 2.27978 1.55504 -1.62334
N 2.12956 -0.01060 -0.23951
H 2.63712 0.55380 0.43444
H 2.49028 -0.95361 -0.12550
N 0.00000 0.00000 2.10979
H -0.92387 -0.30948 2.39647
H 0.65593 -0.64590 2.53571
C 0.23555 1.37961 2.58687
H 1.30868 1.58248 2.52498

H -0.06022 1.51397 3.63030
C -0.53246 2.33644 1.69238
H -1.60114 2.12246 1.75439
H -0.37668 3.36933 2.01211
N -0.11818 2.12393 0.28330
H 0.72834 2.65329 0.09741
H -0.83097 2.50500 -0.33053
O -2.09380 0.15106 -0.02903
C -2.95474 -0.75427 -0.05957
H -2.65258 -1.80686 -0.02347
N -4.25467 -0.55114 -0.13198
C -4.83083 0.78939 -0.18932
H -4.03378 1.52763 -0.19241
H -5.47675 0.94834 0.67670
H -5.42843 0.89038 -1.09746
C -5.19777 -1.66668 -0.15432
H -4.65742 -2.61171 -0.10459
H -5.78442 -1.63824 -1.07459
H -5.87489 -1.59708 0.69926
O -0.16590 -2.11820 0.10630
C 0.34081 -3.13160 -0.41387
H 1.04589 -3.04913 -1.25202
N 0.10569 -4.37427 -0.04205
C -0.79429 -4.69434 1.06174
H -1.14820 -3.77463 1.52017
H -0.26050 -5.29116 1.80377
H -1.64159 -5.27453 0.68952
C 0.73565 -5.50636 -0.72024
H 1.37895 -5.14968 -1.52433
H -0.03154 -6.15885 -1.14134
H 1.33566 -6.07661 -0.00872

180°
Ni 0.00000 0.00000 0.00000
N 0.03762 0.00682 -2.12159
H -0.62713 -0.66722 -2.48589
H -0.23508 0.90423 -2.51094
C 1.40665 -0.33876 -2.56202
H 1.58555 -0.07536 -3.60721
H 1.52363 -1.42197 -2.47395
C 2.40580 0.36599 -1.66115
H 3.42911 0.12816 -1.96148
H 2.28439 1.44912 -1.74813
N 2.13461 -0.01267 -0.25288
H 2.62792 0.61766 0.37143
H 2.53135 -0.92909 -0.06952
N -0.00000 -0.00000 2.11089
H -0.93820 -0.28408 2.37818
H 0.63132 -0.65342 2.56173
C 0.25801 1.37782 2.58428
H 1.33467 1.56145 2.52358
H -0.03713 1.51884 3.62704
C -0.49328 2.34689 1.68885
H -1.56533 2.15374 1.75581
H -0.31576 3.37706 2.00592
N -0.08927 2.12335 0.27844
H 0.76111 2.64304 0.08326
H -0.80181 2.50710 -0.33394
O -2.09327 0.17799 0.03917
C -2.95599 -0.67685 -0.25540
H -2.65501 -1.69280 -0.53382
N -4.25579 -0.46066 -0.26026
C -4.83042 0.83587 0.08756
H -4.03233 1.54266 0.29683
H -5.46877 0.73047 0.96729
H -5.43528 1.20090 -0.74484
C -5.20041 -1.51824 -0.61173
H -4.66104 -2.43409 -0.85250
H -5.79187 -1.21469 -1.47766
H -5.87307 -1.70882 0.22686
O -0.24023 -2.11474 -0.02827
C 0.49665 -3.11987 -0.05652
H 1.57326 -3.03377 -0.25781
N 0.09650 -4.35994 0.14036
C -1.29705 -4.67988 0.43392
H -1.82223 -3.77505 0.72962
H -1.33156 -5.40302 1.24988
H -1.77598 -5.11787 -0.44527
C 1.02166 -5.48794 0.03583
H 2.02181 -5.12948 -0.20665
H 0.68790 -6.16795 -0.75031
H 1.05519 -6.02808 0.98357

Listings S8. Cartezian coordinates of the starting reagents, product and intermediate for the ^{3}**R**$_1$ → ^{3}**P**$_1$ reaction. All geometries are optimized at the ωB97X-D4/ma-def2-TZVP level with the C-PCM dmf solvation model.

ethylenediamine
N -0.42923754848412 -2.39957180734164 -1.78268938770550
H 0.43849104917729 -2.69591366669415 -1.34993561426794
C -1.53113835575902 -2.51707225158867 -0.82200341166020

H -0.29932648406416 -1.42497540770741 -2.03538611151217
H -1.40621738497678 -1.87129458875101 0.05827668577898
H -1.56212627746102 -3.55138415658273 -0.46256201082954
C -2.85794992645038 -2.18213623576558 -1.48904728511194
H -2.82601536114237 -1.14850314797648 -1.85028375174761
N -3.95961671771579 -2.29737950783530 -0.52761623351130
H -2.98410579535305 -2.82947112146692 -2.36798099948368
H -4.08847122354826 -3.27131916804631 -0.27187217660367
H -4.82745597422233 -2.00341894024379 -0.96179970334544

dimethylformamide
O -2.53029850953118 3.27889670795931 -1.13794652118753
C -3.43722014358626 4.10539827982818 -1.23164930778323
H -3.77549796491091 4.47849394628681 -2.21087803781583
N -4.11012905424775 4.64757066213280 -0.20875392960550
C -3.82321126510611 4.28902874743442 1.16881829486519
H -2.96618767644575 3.61985388913520 1.19476205794665
H -4.68772015678708 3.78991265897131 1.61639774875290
H -3.60034486492459 5.19027499684341 1.74634243398706
C -5.18257183097024 5.60168095324273 -0.43114078605656
H -5.29725445528852 5.78444018406882 -1.49986738062277
H -4.95558445140827 6.54710210587019 0.06950817152437
H -6.12418562679324 5.21157186822670 -0.03413474400473

$^{3}R_{1}$
Ni 0.26588883818939 0.12728873005357 0.00188676988798
N 0.10936327613474 0.08070536466289 -2.10369221760339
H -0.53087182434948 -0.63907415801628 -2.42114297435698
H -0.23791452589874 0.96055910342676 -2.47312930129080
C 1.46467719613201 -0.17944025016555 -2.62794453369002
H 1.54175195211656 0.05010024486778 -3.69422734817462
H 1.67605654572911 -1.24228879628460 -2.49095365742223
C 2.46511044036057 0.64645701940959 -1.83805095241773
H 3.47895472851807 0.46801764961514 -2.20728859752804
H 2.24354409658337 1.71040080884527 -1.95927731458758
N 2.32842948632639 0.31576806016524 -0.40710107874205
H 2.81798495057874 0.99769049214706 0.16188708715314
H 2.75646355371239 -0.58522385138363 -0.21656557526845
N 0.47544453105325 0.20815945317614 2.09728203146094
H -0.36213283512291 -0.18129818822138 2.51919978741689
H 1.25778181584233 -0.33329291408011 2.44787079914682
C 0.61639612410668 1.62621921354461 2.47840283554985
H 1.64802013642744 1.92666649708673 2.27645096372508
H 0.41919757002454 1.78812995139990 3.54171838232493
C -0.33301325890975 2.45955705855832 1.63462210755253
H -1.36365039886510 2.14918780933034 1.82269357661813
H -0.24227308533683 3.51743633562438 1.89626559513062
N -0.04101835575099 2.21493810517668 0.20852324423667
H 0.79441902972950 2.72568581281563 -0.06139550878673
H -0.79030748764475 2.57803334230890 -0.37097486069744
O -1.79497512867968 -0.24386043833389 0.33029067149427

C -2.71442259969522 0.18261791359477 -0.39006732357002
H -2.51150139290720 0.85342248185847 -1.23334659986782
N -3.99329511677033 -0.11180672075643 -0.22477126797300
C -4.44799906381668 -0.99308865644535 0.84084197583533
H -3.59320235273690 -1.33088596674069 1.42145258095483
H -4.96147989042231 -1.85458879962203 0.40711141155169
H -5.14381297930254 -0.45428785524070 1.48849684440460
C -5.01285541241086 0.43109636001617 -1.11097443494063
H -4.54540258430492 1.06615581269650 -1.86311817132570
H -5.72843009072083 1.02200197375493 -0.53432723881115
H -5.54374634664039 -0.38485841335505 -1.60723047990457
O 0.56453709658484 -1.97541187693772 -0.05490520768806
C -0.14194987548895 -2.75710587030729 0.60645856448577
H -0.95468113725919 -2.38552183337513 1.23865583980951
N 0.00222527017956 -4.07307848026602 0.61383716201491
C 1.02561992040593 -4.74212868060248 -0.17517359426065
H 1.56664607270069 -4.00664810417160 -0.76526474384732
H 0.55454138789632 -5.47125977808898 -0.83881022865216
H 1.71993017845138 -5.26388001559211 0.48834947865988
C -0.85773293368496 -4.91668101144911 1.43074474957632
H -1.57477698825656 -4.29636152500534 1.96813103292064
H -0.25447590372740 -5.47565688643119 2.15036001110888
H -1.39719262908028 -5.62327652326302 0.79538970838693

$^{3}I_{1}$

Ni 1.33713161028104 1.26472327368787 -0.11835847780544
N 0.72193072874046 0.77706634242264 -2.06766044181684
H 0.08492089559058 -0.02343768348838 -2.06883665643650
H 0.24565568789723 1.55204149748711 -2.51787300482555
C 1.93649807638620 0.43052400210890 -2.82734947630495
H 1.76257652215430 0.43921750552620 -3.90727795115957
H 2.22861827463668 -0.58307330113778 -2.54054117108236
C 3.04888550985087 1.40066816371633 -2.46786652180345
H 3.96291414573377 1.15346004016572 -3.01549305106243
H 2.75481665449999 2.41746372137711 -2.74256994096391
N 3.25029943177214 1.36683015192021 -1.00704981818236
H 3.82244613061292 2.14952359801985 -0.70974681670422
H 3.75142261491346 0.52324912818555 -0.74555928067399
N 2.00017129786946 1.79798483629620 1.81361967709013
H 1.30047996361553 1.50288636555780 2.48764545010649
H 2.87008389374960 1.35098120477838 2.08102961667552
C 2.14066712116067 3.26576640577436 1.84940417437803
H 3.08446597888977 3.52486872511058 1.36204461266066
H 2.17210108538327 3.65151603293581 2.87222080753433
C 0.98381969459295 3.88948825287893 1.08768477751205
H 0.03877485935644 3.61556003020221 1.56276536457797
H 1.06792486868860 4.97978887943161 1.09837990380736
N 0.97231082542422 3.34759776847599 -0.28491243906039
H 1.69806497429616 3.79599456322220 -0.83585368642806
H 0.09444603848241 3.56731485402391 -0.74371989258387
O -0.57496251260159 0.97462037972652 0.75005687171006

C -1.66072940014956 1.18078095453348 0.18033195042937
H -1.70193277610874 1.57169906533467 -0.84309026234847
N -2.84287673185214 0.95704106482831 0.72890147613846
C -2.96687585615591 0.44945595853630 2.08783754143449
H -2.01516223294493 0.03073028060533 2.40703057471177
H -3.73324851968547 -0.32659689932583 2.10668812391337
H -3.25752317764181 1.25635133546881 2.76627145048431
C -4.07525787342037 1.28013078448367 0.02501406793133
H -3.84177724549840 1.64605834850621 -0.97471323900413
H -4.62457865112784 2.05122446695357 0.57100207015228
H -4.69778048552734 0.38647211861579 -0.05368924085487
O 1.72281450441425 -0.80632137243675 0.22377441269908
C 1.17467060767829 -1.42264128039771 1.15496497878604
H 0.53495908573480 -0.90424072842252 1.87582892747536
N 1.28892936304867 -2.72583120391593 1.36040510652040
C 2.09062407661862 -3.57748283974732 0.49462108695265
H 2.60147184067671 -2.96487750534905 -0.24465229082003
H 1.44541956289110 -4.30135774698705 -0.01056343302321
H 2.82630936689736 -4.11750746822564 1.09504935351749
C 0.57459268955343 -3.37847128774626 2.44770673157831
H 0.00904537403654 -2.63768536067790 3.01254223716375
H 1.28389140403060 -3.87580458384984 3.11351753952781
H -0.11500187557828 -4.12329852651509 2.04223313143523
N -0.52135081631017 -2.01604150867484 -1.87419388052944
H 0.41127018931580 -2.20067548711579 -1.52155407267321
C -1.51925313486255 -2.31780157923155 -0.84335507131389
H -0.65066027738493 -2.63562058791390 -2.66777679362101
H -1.33876243166419 -1.67195079321894 0.02016417147002
H -1.46039434187451 -3.35717361683545 -0.48780671021128
C -2.92041248385658 -2.05434825678468 -1.37402351995569
H -3.01601467233788 -0.99387574138931 -1.62477532804677
N -3.92055188652185 -2.38714656373081 -0.35616181689049
H -3.05743086980569 -2.61909278177124 -2.30848753534590
H -3.93703704566304 -3.39106723079575 -0.20758259501685
H -4.84585165090118 -2.13565516521293 -0.68564577182497

$^{3}TI_{1}$

Ni 0.85957850640252 1.43850748997119 -0.38706590278936
N 0.34898138191626 1.11754015150478 -2.37360343727013
H -0.27170695042880 0.31240764546938 -2.32435064322821
H -0.13336846161232 1.89257388833066 -2.81339566457459
C 1.55616331074544 0.73916344532431 -3.13876738593313
H 1.46749958761304 1.02010488254262 -4.19053856567929
H 1.65339869526876 -0.34791191413791 -3.08904311920437
C 2.78382459305262 1.38810975976611 -2.52165328470082
H 3.69112580507697 1.03691872926052 -3.02125616258487
H 2.72946544076310 2.47412749180372 -2.63421587437830
N 2.79041084489237 1.08826067085077 -1.07910914186501
H 3.50019255698858 1.63522965999056 -0.60415448749383
H 3.00958392416822 0.11103706447256 -0.90163744518573
N 1.42679037805306 1.40860784392553 1.60001151414046

H 0.62745453696811 1.16151218283448 2.17447687082823
H 2.14062464031812 0.70126097886003 1.74451698954145
C 1.90340494432364 2.76457699872886 1.92939802999887
H 2.92311093931154 2.86568383188182 1.54858529034870
H 1.92540568610043 2.94168197350157 3.00791216875878
C 0.99833060638267 3.77757609534867 1.24567946710204
H -0.02332182565802 3.66853698916471 1.61669701770775
H 1.33457374369026 4.79637929796095 1.45431539001280
N 0.97963396147150 3.49337745426296 -0.20663075748467
H 1.82358059976811 3.85531542553913 -0.64068215742292
H 0.20045141477853 3.96870573766043 -0.64989301470820
O -1.08559714972960 1.34827975227532 0.32442438160891
C -2.14118521276947 1.56790202276237 -0.30040515184571
H -2.13337793893097 1.80374227428578 -1.36999215689206
N -3.34321866223767 1.54066926817587 0.24466585360076
C -3.54101562044643 1.27867037742709 1.66377799909046
H -2.59016597249664 1.01654073104235 2.12092343407464
H -4.24775554471529 0.45478039975077 1.78355906108117
H -3.94998595086191 2.17025820744053 2.14570280718699
C -4.53774358806527 1.79057036465914 -0.55076062482421
H -4.25642468350612 1.98238126978110 -1.58590055013837
H -5.07127989555219 2.65734274907689 -0.15357374952186
H -5.19301107039600 0.91726125564181 -0.51047461203730
O 2.46588848497808 -1.31650125431340 0.82197790830730
C 1.95701063020721 -2.09547262882227 1.63500152309614
H 1.58693838680208 -1.74000721540579 2.60843988009572
N 1.78807856865717 -3.40547824014623 1.45439031062216
C 2.21299386484029 -4.07676255093620 0.23703564005906
H 2.75794853460323 -3.37563737929487 -0.39081077820672
H 1.34097955481445 -4.45230218850118 -0.30615739390206
H 2.86078104365973 -4.91884067518067 0.49307365216677
C 1.12676190443317 -4.22481249442896 2.45693963512039
H 0.85591092833813 -3.60820408820485 3.31418070871071
H 1.79454661994079 -5.02513266997695 2.78628398905047
H 0.22084938478580 -4.66975047207089 2.03569123947387
N 0.04938017220053 -1.66066997013267 -1.26572309640478
H 0.96786447523815 -1.63144663606890 -0.83516322067846
C -0.97210359287067 -2.04214486074464 -0.28761691114498
H 0.10128760677471 -2.35530255522847 -2.00365697225221
H -0.92057867472746 -1.34128144882252 0.55147373010917
H -0.82552407262953 -3.05505832495745 0.11579391222785
C -2.35635201786358 -1.96416438699165 -0.92682156916578
H -2.51655357053690 -0.95367260207430 -1.31494540023125
N -3.46523549742969 -2.30846448857078 -0.03477378159156
H -2.38916241721151 -2.63884070275117 -1.78969764244377
H -3.44277019198790 -1.71689433104126 0.78933687491739
H -3.35336769566339 -3.26084028247144 0.29820537674555

$^{3}I_2$

Ni 0.43605228957073 0.98434816713819 -0.39386265208864
N 0.03823516270980 1.07123322966100 -2.48010649258032

H -0.80972899136278 0.58046249321971 -2.74284845050824
H -0.08500243711538 2.03391587633950 -2.77899297382472
C 1.20066187828921 0.48457582449073 -3.17486025983233
H 1.21814680396613 0.74635605489778 -4.23652625390201
H 1.12524547875884 -0.60322148008364 -3.10214716720822
C 2.47702517586736 0.95674788498475 -2.49938535235081
H 3.34845973740314 0.52308262010251 -2.99812448133635
H 2.55208609845826 2.04462585112876 -2.58019091925375
N 2.42598869114375 0.60925426676614 -1.06718441357120
H 3.12587103443693 1.13458341017226 -0.55465440289638
H 2.65832962665275 -0.36971779204437 -0.92983596244328
N 0.93937611506078 1.01318974467797 1.65628355069629
H 0.09136338168280 0.87302764571397 2.19580899636971
H 1.57752827783900 0.25556069552839 1.88434251262074
C 1.53656547206281 2.31980281624103 1.97685872903719
H 2.58110582935110 2.30108854058564 1.65307897585700
H 1.52334839934260 2.52895859008366 3.05058044350427
C 0.79021812616811 3.40680685929251 1.22230672537023
H -0.25595667742906 3.42211565396962 1.53655014219590
H 1.22592878604588 4.38640475112716 1.43953954448396
N 0.82489974304741 3.09186406529791 -0.21881889994236
H 1.74070509539638 3.32225255784922 -0.59241439049418
H 0.16588940038816 3.67738003450799 -0.72037354265611
O -1.57502474660277 1.37864560837502 0.19470452460021
C -2.36357390870654 2.11562582667484 -0.41808754470809
H -2.03601135366863 2.71700884683018 -1.27513981894758
N -3.64666502298400 2.25165341259039 -0.11925069992983
C -4.25702122847872 1.52075720723208 0.98149946592935
H -3.53866917697792 0.81248367529664 1.38669970075910
H -5.13498770553201 0.98390607109342 0.61515153184728
H -4.56835197973980 2.21855626474477 1.76328220659896
C -4.50117757688905 3.17031527381977 -0.85693606234956
H -3.92682912490917 3.65301109444942 -1.64751277832916
H -4.89476182582736 3.93430196195723 -0.18180981864243
H -5.33706159413723 2.62444686925251 -1.30073998400718
O 2.61386547200920 -1.48926574489787 1.37851846183347
C 2.96832190929208 -2.46087741682658 2.05679585839267
H 3.44376145836778 -2.32826038088705 3.03892067809604
N 2.83181790016372 -3.73527995454015 1.69607561771435
C 2.21537933364405 -4.09394921525228 0.42768411488770
H 2.35535940464664 -3.28606341345078 -0.28842109134255
H 1.14462918347149 -4.28090474509619 0.55463282706654
H 2.69101180233911 -4.99941849154039 0.04795727725646
C 3.20284707283900 -4.81959266679019 2.59218645127367
H 3.64236027218278 -4.40869344197019 3.50111635332155
H 3.93064714174037 -5.47343697968871 2.10570885051889
H 2.32039541680706 -5.40808246061500 2.85786073984237
N 0.10902849434400 -1.12888843351849 -0.30900265621992
H 0.87075703624373 -1.41898662470323 0.30234019739609
C -1.16046270711016 -1.66811491423021 0.20917716032537
H 0.30734182048049 -1.56828291976418 -1.20360436279801

H -1.47129400134750 -1.06793688767977 1.06606844287751
H -1.02768829729969 -2.70271032118516 0.55033396955299
C -2.24101602993360 -1.63426251975789 -0.85964682234572
H -2.41547166995293 -0.60138275147919 -1.16596355843290
N -3.49219980915189 -2.17718313390421 -0.32541268513152
H -1.88028719103511 -2.18163328186072 -1.74400044696617
H -3.39656902245771 -3.17555192003700 -0.16952446597155
H -4.23473824356444 -2.06665085428917 -1.00668863921476

$^{3}I_{3}$

Ni 0.42665169557001 0.99801175374765 -0.30707151668578
N -0.14182528267556 1.00380549472531 -2.32679480876625
H -0.98676593359771 0.42681553786948 -2.39057458252279
H -0.36056870721633 1.93110070004796 -2.67455502969775
C 0.96054724435909 0.42244230748626 -3.10970813241634
H 0.88072901171117 0.66152581290817 -4.17419672652730
H 0.91057558957751 -0.66571789152625 -3.01425683289307
C 2.28681089424354 0.92431875337118 -2.56192984630505
H 3.12083256224697 0.48945441013627 -3.12019319286866
H 2.33904910739707 2.01069980678735 -2.67252931484601
N 2.36154070299743 0.61048643864394 -1.12089469975975
H 3.09342668863825 1.15790940944601 -0.68093961591376
H 2.62570697046050 -0.36172287354243 -0.99429291786615
N 1.07661159353007 1.09889668468984 1.71624549322630
H 0.25277637205265 0.98699501798919 2.29935722773695
H 1.72723630426983 0.37125833446184 1.99092631662029
C 1.67653984880870 2.42624776883137 1.94534635253384
H 2.69471522825625 2.40747336619266 1.54682571686598
H 1.73609138492128 2.67313671702469 3.00926503840075
C 0.85914377681487 3.47254706230415 1.20862496919641
H -0.16250538699423 3.48217034294501 1.59490668020555
H 1.29129452179949 4.46557694488730 1.36234916954200
N 0.79948883697431 3.10763895901336 -0.21910644363215
H 1.67824780509626 3.34886545141287 -0.66724006905243
H 0.08723854129567 3.65416331230025 -0.69121648309740
O -1.52722548585415 1.39346470161346 0.45596890021758
C -2.41598174965232 1.97300337248162 -0.18805365184740
H -2.19689859904845 2.48196109129899 -1.13406969371698
N -3.68705708975127 2.03852918831786 0.17902217896851
C -4.16550931830758 1.41219337026585 1.40250502059267
H -3.34535448259487 0.88487600138301 1.88323514183610
H -4.96488852898196 0.70738653047916 1.16173977869771
H -4.55817725577813 2.17601648014991 2.07842108922632
C -4.66933436718484 2.75333871024392 -0.62266550783586
H -4.19014835256644 3.16806392637572 -1.50911613253641
H -5.10722650483645 3.56562496014814 -0.03731874017419
H -5.46402712167312 2.06918629463660 -0.92992520063237
N 0.06157812598468 -1.12975516509931 -0.14845663711392
H 0.66345094557470 -1.37972437393973 0.63030361997180
C -1.27625259951659 -1.71677751191141 0.10368957002182
H 0.46895223963189 -1.60786962440380 -0.94732027820424

H -1.85494257690875 -1.01481963055190 0.70680056988300
H -1.16969759212097 -2.64294550567442 0.67726007790776
C -2.02953011148547 -2.05541257821559 -1.17380528451397
H -1.37891122494441 -2.64676198176119 -1.82690985292636
N -2.46461067982495 -0.86732704345382 -1.91878261424815
H -2.87299339705529 -2.70022674607620 -0.89519430012723
H -3.19068718175817 -0.38946666007377 -1.39467387156132
H -2.89828846188367 -1.15850342838721 -2.78853893336205

^{3}TI$_{2}$

Ni 1.17932344500874 -0.32491903537534 -0.33856399670443
N 1.40393467539060 -0.36350020493382 -2.40634437271563
H 0.71085522173578 -1.02281641409848 -2.75068833306430
H 1.20416499269602 0.53332569092800 -2.83730039918647
C 2.79060134513655 -0.76979911036788 -2.69063816837102
H 3.08118353351222 -0.55404934026963 -3.72230320499273
H 2.86938676334824 -1.84972312714969 -2.54216901151414
C 3.71471422032688 -0.04781422136094 -1.72311513833448
H 4.75271276680751 -0.35020675866752 -1.88358619603001
H 3.64699883157861 1.03065437112658 -1.88783731636962
N 3.27201744358387 -0.32192254584865 -0.33846117334091
H 3.67683482796959 0.35693595460464 0.29755499846933
H 3.61883705543198 -1.22841367579189 -0.03928217248723
N 0.82579539353943 -0.11565134093067 1.70878261361274
H -0.17097139711039 -0.26528866170643 1.84704361636375
H 1.32020243204864 -0.78111536509713 2.29259327942122
C 1.17923760872153 1.26499390766403 2.08440456252830
H 2.26894222008176 1.34898707201391 2.07671072410037
H 0.82759745497171 1.52040140065382 3.08819461906350
C 0.57382168140369 2.21706444795304 1.06575041996707
H -0.51275158615353 2.19690778271254 1.14464609825067
H 0.90572176202845 3.23961626785640 1.26100052272394
N 0.93906164001638 1.77479890925612 -0.29650350920508
H 1.81864897040779 2.19906650350542 -0.57400649972737
H 0.24905794562188 2.09712166998290 -0.96674923267473
O -1.91069797112911 -0.01952774141917 0.56289456330044
C -2.77000406371552 0.85428344713970 0.40920730694496
H -3.18548223147754 1.07604158729040 -0.58462699152885
N -3.28294116931843 1.61482204780538 1.37683700240856
C -2.87598659830332 1.47026034954705 2.76525950427027
H -2.22155537491924 0.60732675731220 2.86427631621905
H -3.76047084219190 1.32904634955664 3.39124110581947
H -2.34801193761254 2.36828455732145 3.09840497413650
C -4.27679853684217 2.63638558081137 1.08740274635701
H -4.49661369791274 2.64316692232158 0.01973033452062
H -3.89996526331988 3.61971596259750 1.38119398863700
H -5.19654163516181 2.43091322688730 1.64132053719723
N 1.02600910228231 -2.42462858467215 -0.21243682833963
H 1.60088372262328 -2.67350124360326 0.58745546009813
C -0.31798011622108 -3.01909988207094 -0.02473487687947
H 1.46748769023611 -2.87811287452733 -1.00791064183643

H -0.85376478324972 -2.42202927145409 0.71654522377003
H -0.23039105573337 -4.03786537445949 0.36485335353883
C -1.10386766090770 -3.06595275069043 -1.32406576970573
H -0.56413903024070 -3.68872984578140 -2.04656444983837
N -1.27816997588982 -1.73011283817230 -1.89726229183407
H -2.05558503924699 -3.57170180654082 -1.11127567883068
H -1.73074552306391 -1.12571033117491 -1.21610080706655
H -1.88059725678818 -1.77792842068367 -2.71077681114114

$^3I_4$

Ni 0.17676454326893 0.93754575387278 -0.14703142825417
N -0.47008589836843 1.19374864703353 -2.17183589378653
H -1.22152620312504 0.56889610839257 -2.44166800087218
H -0.84444700471953 2.13349793269421 -2.26639182144786
C 0.69008505515682 0.99007067556060 -3.05633577841118
H 0.53456739595854 1.42121989314086 -4.04971406609120
H 0.83408290841260 -0.08719394556561 -3.17833649762336
C 1.92647844438669 1.59427642300374 -2.41409871986561
H 2.79869063010805 1.43174908339749 -3.05418110701926
H 1.79352324086255 2.67302317997312 -2.29835266503205
N 2.10523831639603 1.01005573601868 -1.07199809378186
H 2.79236867960916 1.54211035774509 -0.54914110601486
H 2.47729240319961 0.06880814237904 -1.15452124593016
N 1.00288023404228 0.86646236910244 1.82999830430641
H 0.32587595433338 0.50611008566278 2.49486755451148
H 1.80850622019441 0.25291901130607 1.88586637128789
C 1.38187988375238 2.24296871496762 2.20239066668114
H 2.33364836420631 2.47400781383698 1.71684114239417
H 1.52052902319294 2.35360533720768 3.28175424805328
C 0.31205342677049 3.20078188571837 1.70612228367553
H -0.64401512430785 2.96183095931083 2.17921261493234
H 0.57042349996377 4.22852588726767 1.97794701480156
N 0.15594233476372 3.03154719362969 0.25177949257564
H 0.88749997986153 3.53914707129060 -0.23401366696165
H -0.73283093254769 3.40874386199335 -0.06642381927590
O -2.52569391322223 3.28737425763440 -1.14428140053742
C -3.44226296786698 4.11155238865984 -1.23478268535060
H -3.78285345907641 4.47722898156371 -2.21396323715545
N -4.10928145654378 4.64495687305013 -0.21183231648978
C -3.81689927669746 4.29072428228363 1.16725378980233
H -2.96050306018274 3.62180437016058 1.20023640291758
H -4.68219315154806 3.79511776849048 1.61607995240567
H -3.59402045700378 5.19496681279735 1.73934934512979
C -5.18704763213396 5.59717031414631 -0.42825967622870
H -5.30959580817531 5.77836762606220 -1.49610595799933
H -4.95703619434652 6.54211634779992 0.07086498998325
H -6.12282016655765 5.20284568347331 -0.02310116355344
N 0.02664618401075 -1.19517879948322 -0.26077960722815
H 0.85063107055768 -1.66200443621600 0.10237841229974
C -1.16481690858441 -1.58739965418711 0.51621181070466
H -0.07471393183697 -1.50503752556033 -1.22233417207148

H -0.90629199035025 -1.53543943692985 1.57707640988181
H -1.48057048911076 -2.61151278534260 0.29659811888551
C -2.29278212650093 -0.61385354198094 0.21722557512182
H -2.54604404959064 -0.66069917527204 -0.84508150122698
N -1.84033780512499 0.75369726483208 0.52350629079869
H -3.18721193272520 -0.88962143282069 0.78341597284135
H -1.94136180886480 0.93554361805546 1.51641526369833
H -2.40093404389615 1.44877201984296 0.03839360051919

$^3P_1$

Ni 0.00025347306572 -0.00393346607394 -0.00207178459046
N 0.04981406707459 -0.00604877340199 2.13623849705514
H -0.55316748594505 -0.71905328796620 2.53226459147781
H 0.98440945315412 -0.20059158643825 2.48191334237683
N -0.07868587958505 2.11508321370831 0.29780083648707
H 0.55611798967312 2.60501787828736 -0.32331232354947
H -1.00406776429671 2.48507490534204 0.10485011371978
N 2.13597821709204 0.01572920032542 -0.15932959258777
H 2.39877154110028 0.30407567463135 -1.09675902628200
H 2.59077745798798 0.66519660500990 0.47303867197801
C -0.37750038453501 1.33175266431370 2.58700900799109
H -0.12894025391142 1.51197719103764 3.63683935502209
H -1.46430982046939 1.39021610138882 2.48453162237213
C 0.27730528401793 2.38049437940410 1.70498648598202
H -0.02235532852570 3.38317294795474 2.02278576511865
H 1.36421540680523 2.31180369499266 1.79845793560501
C 2.61707980027709 -1.35533501961142 0.09205818987124
H 2.59181854081961 -1.52936998689060 1.17122202834599
H 3.64717316161539 -1.49943873239397 -0.24633851340980
N 0.31339863608677 -2.11738903342757 -0.13628614540638
H 0.19389223202472 -2.52816728327946 0.78485819213199
H -0.34151099043231 -2.59532506544005 -0.74549158485802
C 1.69414554223871 -2.33883301332431 -0.60505830219722
H 1.71670446917109 -2.16284147438990 -1.68397989808685
H 2.03082485227160 -3.36399725629652 -0.42591055949726
N -0.28697067114110 0.19624797712668 -2.11184366022718
H 0.27977081296906 0.93789074058214 -2.50891046284542
H -0.02561937910352 -0.65389464763710 -2.60163609277744
N -2.12936608103877 -0.22379307917521 -0.03368912051996
H -2.43727544486862 -0.99854129822433 0.54391007997040
H -2.58918326524331 0.60305317915393 0.33416805372429
C -1.71859539156811 0.47358413077262 -2.33315760092077
H -2.00859803978204 0.33409407906068 -3.37857692188968
H -1.90432464500248 1.51812968643823 -2.06950771391175
C -2.53879977966167 -0.43597047169882 -1.43514238250361
H -3.60664814207183 -0.25255142493682 -1.58389267957638
H -2.34031219026295 -1.47986934892390 -1.69169840359210
